# SCIGA: Software for large-scale, single-cell immunoglobulin repertoire analysis

**DOI:** 10.1093/gigascience/giab050

**Published:** 2021-09-28

**Authors:** Haocheng Ye, Lin Cheng, Bin Ju, Gang Xu, Yang Liu, Shuye Zhang, Lifei Wang, Zheng Zhang

**Affiliations:** Institute for Hepatology, National Clinical Research Center for Infectious Disease, Shenzhen Third People's Hospital, The Second Affiliated Hospital, School of Medicine, Southern University of Science and Technology, Shenzhen, Guangdong 518112, China; CAS Key Laboratory of Pathogenic Microbiology and Immunology, Institute of Microbiology, Chinese Academy of Sciences (CAS), Beijing 100101, China; Institute for Hepatology, National Clinical Research Center for Infectious Disease, Shenzhen Third People's Hospital, The Second Affiliated Hospital, School of Medicine, Southern University of Science and Technology, Shenzhen, Guangdong 518112, China; Institute for Hepatology, National Clinical Research Center for Infectious Disease, Shenzhen Third People's Hospital, The Second Affiliated Hospital, School of Medicine, Southern University of Science and Technology, Shenzhen, Guangdong 518112, China; Institute for Hepatology, National Clinical Research Center for Infectious Disease, Shenzhen Third People's Hospital, The Second Affiliated Hospital, School of Medicine, Southern University of Science and Technology, Shenzhen, Guangdong 518112, China; Institute for Hepatology, National Clinical Research Center for Infectious Disease, Shenzhen Third People's Hospital, The Second Affiliated Hospital, School of Medicine, Southern University of Science and Technology, Shenzhen, Guangdong 518112, China; Shanghai Public Health Clinical Center, Fudan University, Shanghai, 201508, China; Department of Radiology, National Clinical Research Center for Infectious Disease, Shenzhen, Third People's Hospital, The Second Affiliated Hospital, School of Medicine, Southern University of Science and Technology, Shenzhen, Guangdong 518112, China; Institute for Hepatology, National Clinical Research Center for Infectious Disease, Shenzhen Third People's Hospital, The Second Affiliated Hospital, School of Medicine, Southern University of Science and Technology, Shenzhen, Guangdong 518112, China

**Keywords:** software, single-cell, immunoglobulin repertoires, COVID-19, antibody

## Abstract

**Background:**

B-cell immunoglobulin repertoires with paired heavy and light chain can be determined by means of 10X single-cell V(D)J sequencing. Precise and quick analysis of 10X single-cell immunoglobulin repertoires remains a challenge owing to the high diversity of immunoglobulin repertoires and a lack of specialized software that can analyze such diverse data.

**Findings:**

In this study, specialized software for 10X single-cell immunoglobulin repertoire analysis was developed. SCIGA (Single-Cell Immunoglobulin Repertoire Analysis) is an easy-to-use pipeline that performs read trimming, immunoglobulin sequence assembly and annotation, heavy and light chain pairing, statistical analysis, visualization, and multiple sample integration analysis, which is all achieved by using a 1-line command. Then SCIGA was used to profile the single-cell immunoglobulin repertoires of 9 patients with coronavirus disease 2019 (COVID-19). Four neutralizing antibodies against severe acute respiratory syndrome coronavirus 2 (SARS-CoV-2) were identified from these repertoires.

**Conclusions:**

SCIGA provides a complete and quick analysis for 10X single-cell V(D)J sequencing datasets. It can help researchers to interpret B-cell immunoglobulin repertoires with paired heavy and light chain.

## Background

The diversity of B-cell immunoglobulin is an important characteristic of the adaptive immune system. It is developed through the rearrangement of variable (V), diversity (D), and joining (J) gene segments, which is referred to as V(D)J; the pairing of heavy and light chains; and somatic hypermutation (SHM) [1]. Exposure to infections and environmental factors shapes the repertoire of B-cell immunoglobulins [2–4] and leads to clonal expansion of immune cells, allowing them to change into different types of cells to respond to a specific antigen. Understanding these immunoglobulin repertoires can help researchers to discover antibodies, monitor vaccination responses, and infer B-cell trafficking patterns [5, 6].

10X single-cell V(D)J sequencing is a powerful tool for investigating paired heavy and light chain repertoires of B-cell immunoglobulins [7]. It has been used in the identification of neutralizing antibodies against severe acute respiratory syndrome coronavirus 2 (SARS-CoV-2) [8], the virus that causes coronavirus disease 2019 (COVID-19) [9]. However, accurately analyzing 10X single-cell immunoglobulin repertoires remains a challenge owing to the high diversity of immunoglobulin repertoires and the lack of specialized software that can analyze such diverse data.

Here, we developed SCIGA (Single-Cell Immunoglobulin Repertoire Analysis), a software package for quickly analyzing the data of 10X single-cell immunoglobulin repertoires. SCIGA performs read trimming, immunoglobulin sequence assembly and annotation, and heavy and light chain pairing by means of a 1-line command. It also computes the statistics of repertoires, including gene usage frequency, SHM rate, length of complementarity-determining region 3 (CDR3), and clonality, and further implements visualization. We profiled the immunoglobulin repertoires of peripheral blood mononuclear cells (PBMCs) from 9 patients with COVID-19 using SCIGA. Finally, we identified 4 neutralizing antibodies against SARS-CoV-2 from these repertoires.

## Methods

**Figure 1:**
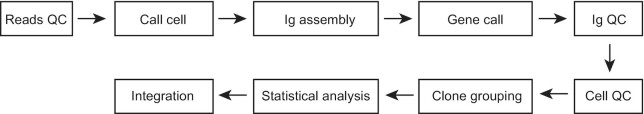
The workflow of SCIGA. The workflow includes quality control of reads, call cell, immunoglobulin (Ig) sequence assembly, V(D)JC gene call, quality control (QC) of Ig sequence, cell QC, group clonal lineage, statistical analysis and visualization, and multiple sample integration analysis.

SCIGA is a software package for the analysis of 10X single-cell immunoglobulin repertoires. It integrates several tools and algorithms into a single workflow. The input data can be raw reads or the output of Cell Ranger (RRID:SCR_017344) [10]. The details of the SCIGA algorithm can be found in the Supplementary Methods and Materials. Briefly, the workflow, which is summarized in Fig. 1, is as follows. (i) Quality control of reads: trim the reads of low quality using Trimmomatic (RRID:SCR_011848) [11]. (ii) Call cell: the 10X system generates a large amount of Gel Beads-in-Emulsion (GEMs) that contain no cell. We need to identify the cell-containing GEMs before further analysis. SCIGA considers the GEMs containing cell(s) when the read number of the GEMs exceeds a threshold (see Supplementary Methods and Materials). (iii) Immunoglobulin sequence assembly: the immunoglobulin sequences for each cell were assembled using SSAKE (RRID:SCR_010753) [12], which is a reliable *de novo* assembler for short reads. (iv) Gene call: to detect the usage of the V(D)J gene and C gene (isotype), SCIGA aligns the assembled immunoglobulin sequences against the V-, D-, and J- gene reference database using IgBLAST (RRID:SCR_002873) [13] and against C-gene reference database using BLAST (RRID:SCR_004870) [14]. The V(D)JC reference databases for humans, mice, and rats were downloaded from the international ImMunoGeneTics information system (IMGT) [15] and embedded in the SCIGA software. (v) Quality control of the immunoglobulin sequence: only the immunoglobulins that are complete, in the correct reading frame, and have no stop codon are retained. (vi) Quality control of the cells: after immunoglobulin sequence assembly and filtering, some cells have multiple heavy or light chains, whereas some cells have only 1 chain. SCIGA reports the heavy and light chain with the highest number of unique molecular identifiers (UMIs) for each cell. A certainty score is calculated for each reported chain (see Supplementary Methods and Materials). The chains with a certainty score less than a given threshold are discarded. Next, the cells without paired heavy and light chains are filtered out. (vii) Clonal lineage grouping: clonal lineage is defined as the cells that have identical V_H_, J_H_, V_L_, and J_L_ genes and identical H-CDR3 length and exceed a given similarity threshold of H-CDR3 nucleotide sequences. (viii) Statistical analysis and visualization: SCIGA calculates a list of statistics, including gene usage frequency, SHM rate, CDR3 length, Simpson index, Shannon entropy, and others. SCIGA subsequently generates figures to show the features of the repertoires. (ix) Multiple sample integration analysis: after analyzing each sample, SCIGA consolidates all of the outputs into one. It identifies the shared immunoglobulins that are potential public antibodies suitable for use against a specific pathogen. Shared immunoglobulins are defined as immunoglobulins from different samples that can be clustered into the same clonal lineage. Clustering is performed as described in Step 7 with cells of all samples.

## Findings

### Comparing SCIGA to existing software

**Figure 2:**
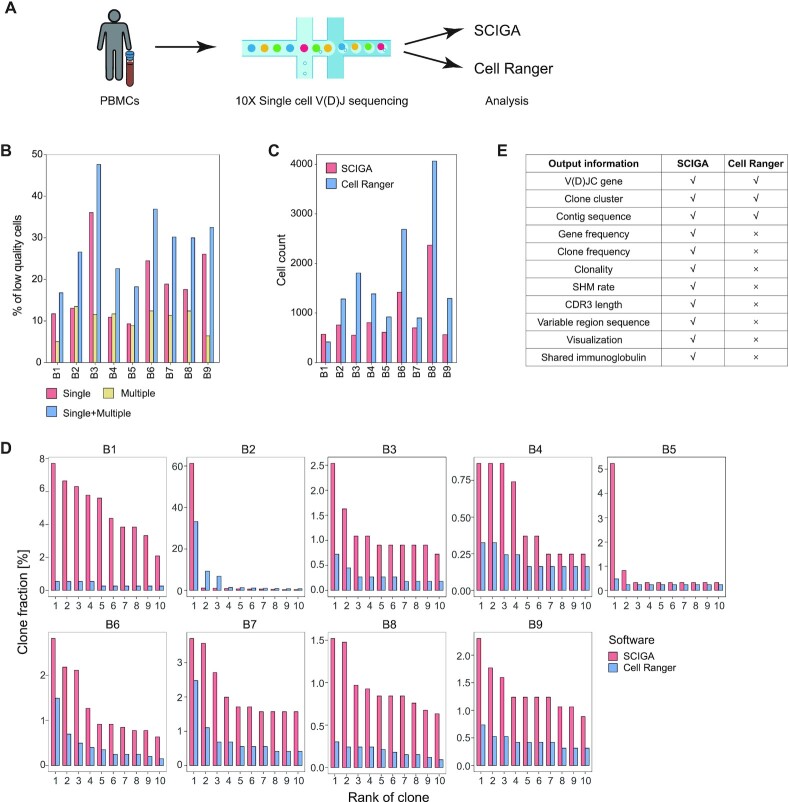
Comparison of SCIGA and Cell Ranger. (A) Flow chart of the experiment. (B) The percentage of low-quality cells in the output of Cell Ranger. Single denotes the cells containing single chain. Multiple denotes the cells containing multiple heavy or light chains. (C) The count of B cells in the output of SCIGA and Cell Ranger. (D) The fraction of the top 10 largest clones analyzed using SCIGA and Cell Ranger. (E) The output information of SCIGA and Cell Ranger.

At the time of this study, Cell Ranger is the only existing software for processing raw data generated by 10X single-cell V(D)J sequencing. A test dataset was therefore built to compare SCIGA to Cell Ranger. The PBMCs from 9 patients with COVID-19 (B1–B9) were collected and 10X single-cell V(D)J sequencing was performed (Fig. 2A and Supplementary Table S1). The raw data were analyzed using SCIGA and Cell Ranger (v3.1.0) with the default parameters. The comparison mainly focused on the following aspects: (i) Cell quality control: for Cell Ranger, the final results still included low-quality cells that had either multiple heavy or light chains or only 1 chain. In our test dataset, the mean percentage of low-quality cells was 29% (range, 16.8–47.6% per sample, Fig. 2B). SCIGA implements the cell quality control process (Step 6 described in Methods) and only outputs the high-quality cells. The cell count was generally less in the output of SCIGA compared to Cell Ranger owing to the strict quality control process (Fig. 2C). (ii) Detecting B cell clonal lineage: Cell Ranger clusters B cells into a clonal lineage when cells have identical nucleotide sequences of CDR3. However, it will break up the clonotypes that are clonally related in fact when the SHM falls within the CDR3 region. SCIGA uses a popular clonal grouping method (Step 7 of Methods), which considers the SHM and allows mismatch in the CDR3 region. Therefore, SCIGA could detect a larger clonal lineage than Cell Ranger (Fig. 2D). (iii) Output information: the output of Cell Ranger is quite limited, and some important information, such as SHM rate, is not included. SCIGA outputs the necessary information, including gene frequency, clone frequency, clonality, SHM rate, CDR3 length, the immunoglobulin variable region sequence, and others (Fig. 2E). Moreover, SCIGA is able to implement visualization to display the features of the repertoires. (iv) Detecting shared immunoglobulin: this is a specific function in SCIGA, and it could detect the shared immunoglobulin across samples.

### Determining the features of immunoglobulin repertoires of COVID-19 using SCIGA

**Figure 3:**
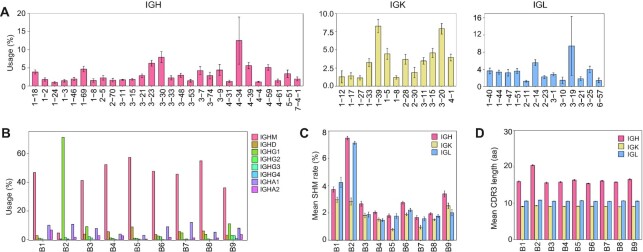
The features of the single-cell immunoglobulin repertoires of 9 patients with COVID-19. (A) The mean usage frequency of V-genes in the repertoires. Only the genes with frequency >1% are shown. (B) The usage frequency of isotypes in the repertoire of each patient. Colors denote the isotypes. (C) The mean SHM rate of V-genes. (D) The mean CDR3 length in the repertoire of each patient. The error bars represent the standard error.

We performed a trial study to show the usage and performance of SCIGA. The 10X V(D)J sequencing data of the 9 patients with COVID-19 were analyzed, and features of the immunoglobulin repertoires were determined. A total of 8,358 B cells were detected (range, 571–2,371 cells per sample, Fig. 2C). We focused on the genes used in ≥1% of B cells for the V-gene usage (Fig. 3A and Supplementary Fig. S1). The top 3 gene families were *IGHV4-34* (12.51%), *IGHV3-30* (7.95%), and *IGHV3-23* (6.30%) for the heavy chain and *IGLV3-19* (9.46%), *IGKV1-39* (8.26%), and *IGKV3-20* (7.90%) for the light chain. *IGHV4-34* and *IGLV3-19* had elevated usage frequency in the repertoire of Patient B2 and reached 63.98% and 64.39%, respectively. IGHM had the highest usage in the repertoires of most patients, except for Patient B2, who showed the highest usage of IGHG1 (Fig. 3B). The SHM levels were low in the repertoires of most patients (<2%, Fig. 3C and Supplementary Fig. S2). However, Patient B2 showed a high SHM level in the IGH chain (7.48%) and IGL chain (7.13%). Moreover, Patient B2 had an elevated CDR3 length for its immunoglobulin repertoire (Fig. 3D and Fig. S3).

### Clonal lineage analysis using SCIGA

**Figure 4:**
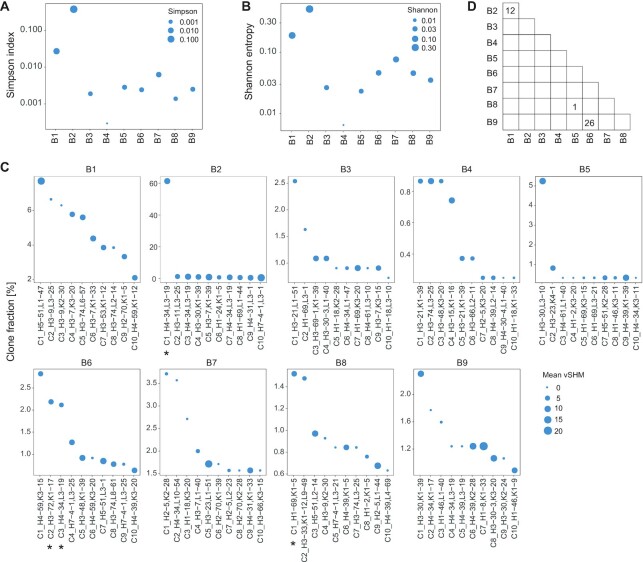
The B-cell clonal expansion of 9 patients with COVID-19. (A) The Simpson index. (B) The Shannon entropy denotes the clonality of the repertoire of each patient. (C) The top 10 largest clones in the repertoire of each patient. The x-axis captures the clone ID and used V-genes. The initial of the gene names denotes the chain: H: IGH; K: IGK; L: IGL. *Screened antibody candidate. (D) Number of immunoglobulins shared between patients. Blank indicates zero.

Clonal lineages were grouped using SCIGA with the default parameters. We used the Simpson index and Shannon entropy to determine the clonality of the immunoglobulin repertoires (Fig. 4A and B). Both indices showed that the repertoires of Patient B2 underwent clonal expansion. The top 10 largest clones of each patient were reviewed (Fig. 4C and Fig. S4). The frequency of the largest clone for most patients was <8%. However, the largest clone in Patient B2 reached a frequency of 61.21%. This strongly expanded clone used the *IGHV4-34* and *IGLV3-19* genes, with an 8.82% mean SHM rate and 23-amino acid H-CDR3 length. We determined the most used V-genes in the top 10 largest clones of all patients. It was observed that *IGHV4-34* (9 clones) was the most used gene in the heavy chain, *IGKV1-39* (9 clones) and *IGKV3-20* (9 clones) were the most used genes in the light chain, and *IGHV4-34: IGLV3-19* (5 clones) was the most used gene pair (Fig. S5A–C). Next, the shared immunoglobulin sequences across patients were determined using SCIGA. There were 12 immunoglobulins shared between Patients B1 and B2, 1 immunoglobulin was shared between Patients B5 and B8, and 26 immunoglobulins were shared between Patients B6 and B9 (Fig. 4D and Supplementary Table S2).

### Identification of neutralizing monoclonal antibodies

**Figure 5:**
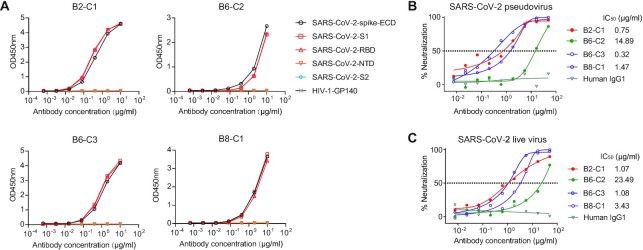
Characteristics of the spike-specific monoclonal antibodies. (A) The binding profile of selected monoclonal antibodies to the extracellular domain and subdomains of the SARS-CoV-2 spike by ELISA. HIV-1-GP140 is the negative control. (B, C) Neutralization activity of selected monoclonal antibodies against the pseudovirus (B) and live SARS-CoV-2 (C). The dashed line indicates a 50% reduction in viral infectivity. Human IgG1 is the negative control. Results presented here are representative of 2 independent experiments.

It was hypothesized that IgGs with higher clonal expansion may be SARS-CoV-2–specific antibodies in the patients with COVID-19. Thus, monoclonal antibodies (mAbs) were screened for the following criteria: IgG antibodies in the clone with fraction ≥1% and cell number ≥20 (see Supplementary Methods and Materials). Four mAbs met the criteria and were expressed: B2-C1, B6-C2, B6-C3, and B8-C1 (Fig. 4C and Supplementary Table S3). Remarkably, enzyme-linked immunosorbent assay (ELISA) revealed that all 4 mAbs were SARS-CoV-2 RBD (receptor binding domain)-specific antibodies, which bound to the extracellular domain (ECD), the S1 subunit, and the RBD of the SARS-CoV-2 spike (Fig. 5A). They did not bind to the N-terminal domain (NTD) and the S2 subunit. The monoclonal antibodies could neutralize SARS-CoV-2 by blocking the attachment of RBD to the receptor (angiotensin-converting enzyme 2 [ACE2]) on host cells. B2-C1 and B6-C3 exhibited potent neutralizing activity (half-maximal inhibitory concentrations [IC_50_] = 0.75 and 0.32 μg/mL, respectively) against SARS-CoV-2 pseudovirus, whereas B8-C1 (1.47 μg/mL) and B6-C2 (14.89 μg/mL) were moderate and weak neutralizing antibodies (Fig. 5B). Similar results were found for the neutralization of the 4 mAbs against SARS-CoV-2 live virus (Fig. 5C).

## Discussion

In this study, we developed the SCIGA software for 10X single-cell immunoglobulin repertoire analysis. SCIGA is easy to use and allows researchers to quickly perform advanced analysis on 10X V(D)J sequencing datasets. Cell Ranger has previously been used for 10X single-cell immunoglobulin repertoire analysis. However, this software includes low-quality cells in the output and disregards the effect of SHM when defining clonal lineage. In addition, some important information about repertoires, including, e.g., the level of SHM, is not included in the output of Cell Ranger. SCIGA performs the quality control process for cells and defines the clonal lineage including the effect of SHM. Larger clones can be detected by using SCIGA. Moreover, SCIGA generates the needed statistical outputs and implements visualization. It is therefore a more efficacious tool for researchers.

In this study, SCIGA was used to analyze the single-cell immunoglobulin repertoires of the PBMCs of patients with COVID-19. Large-scale clone expansion was not observed in most patients. In Patient B2, however, B cells expanded, which was indicated by a large size of clonal lineage with the IgG isotype. This indicates that Patient B2 likely generated neutralizing antibodies against SARS-CoV-2.

Finally, we tried to identify the SARS-CoV-2–responding antibodies. In previous work, immunoglobulins with an SHM rate <2% were excluded in screens for neutralizing antibodies [8]. However, some studies have shown that several potent neutralizing antibodies against SARS-CoV-2 have low SHM rates [16–18]. Therefore, we included the antibodies with low SHM levels in our work. Four neutralizing antibodies with different potency were identified using our criteria. This demonstrates that SCIGA is useful for 10X single-cell immunoglobulin repertoire analysis.

## Data Availability

The datasets supporting the results of this article are available in the NCBI repository and can be accessed with PRJNA682839. All additional supporting data and materials are available in the *GigaScience* GigaDB database [19].

## Availability of Supporting Source Code and Requirements

Project name: SCIGA

Project home page: https://github.com/sciensic/SCIGA

Operating system(s): Linux

Programming language: Perl

Other requirements: IgBlast 1.15.0 or higher, Blast 2.9.0 or higher, R (optional), ggplot2 (optional)

License: GNU GPL-3.0 License

RRID:SCR_021002, Biotools ID: sciga

## Additional Files

**Figure S1:**The usage frequency of V-genes in the repertoire of each patient. Only the genes with frequency >1% are shown.

**Figure S2:** Distribution of the SHM rate in the repertoire of each patient. Color denotes the chain, with IGH shown in red, IGK in yellow, and IGL in blue.

**Figure S3:** Distribution of the CDR3 length in the repertoire of each patient. Color denotes the chain, with IGH shown in red, IGK in yellow, and IGL in blue.

**Figure S4:** Fraction of all clones in the repertoire of each patient. The x-axis captures the clone rank and y-axis captures the clone fraction. *Selected antibody candidate.

**Figure S5:** Number of the used V-genes of the top 10 largest clones of all patients. **(A)** The count of the used V-gene for the heavy chain. **(B)** The count of the used V-gene for the light chain. **(C)** The count of the used V-gene pair. The initials of the gene names denote the chain: H: IGH; K: IGK; L: IGL.

**Figure S6:** Two different examples show how to chose the threshold: (**A**) having and (**B**) not having a large difference in the read counts between cells containing GEMs and background.

**Table S1:** Information on the 9 patients with COVID-19.

**Table S2:** Information on the shared immunoglobulins. Columns 7–11 denote the number of shared immunoglobulin sequences.

**Table S3:** Information on the 4 antibody candidates.

giab050_GIGA-D-20-00341_Original_Submission

giab050_GIGA-D-20-00341_Revision_1

giab050_GIGA-D-20-00341_Revision_2

giab050_GIGA-D-20-00341_Revision_3

giab050_Response_to_Reviewer_Comments_Original_Submission

giab050_Response_to_Reviewer_Comments_Revision_1

giab050_Response_to_Reviewer_Comments_Revision_2

giab050_Reviewer_1_Report_Original_Submission Rachael Bashford-Rogers -- 1/13/2021 Reviewed

giab050_Reviewer_1_Report_Revision_1 Rachael Bashford-Rogers -- 4/6/2021 Reviewed

giab050_Reviewer_2_Report_Original_Submission Yariv Wine -- 2/20/2021 Reviewed

giab050_Supplemental_Files

## Abbreviations

ACE2: angiotensin-converting enzyme 2; BLAST: Basic Local Alignment Search Tool; CDR3: complementarity determining region 3; COVID-19: coronavirus disease 2019; ECD: extracellular domain; ELISA: enzyme-linked immunosorbent assay; GEMs: Gel Beads-in-emulsion; IC50: half-maximal inhibitory concentration; Ig: immunoglobulin; mAb: monoclonal antibody; NTD: N-terminal domain; PBMC: peripheral blood mononuclear cell; RBD: receptor binding domain; SARS-CoV-2: severe acute respiratory syndrome coronavirus 2; SHM: somatic hypermutation; UMI: unique molecular identifier.

## Ethics, Consent, and Permissions

This study was conducted according to the ethical principles of the Declaration of Helsinki. Ethical approval was obtained from the Research Ethics Committee of Shenzhen Third People’s Hospital (2020-207). All participants provided written informed consent for sample collection and subsequent analyses. Raw data has been approved for release by the Ministry of Science and Technology (MOST) of People's Republic of China, with the reference number 2021BAT3238.

## Competing Interests

The authors declare that they have no competing interests.

## Funding

This study was supported by the National Science Fund for Distinguished Young Scholars (82025022), the Sanming Project for Medicine of Shenzhen (SZSM201612053), the National Key Plan for Scientific Research and Development of China (2020YFC0848800, 2020YFC0844200), the National Science and Technology Major Project of the Infectious Diseases (2018ZX10301404 to Z.Z. and S.Z.), the Science and Technology Innovation Committee of Shenzhen Municipality (JSGG20200207155251653, 2020A1111350032, JCYJ20190809115617365, JSGG20200807171401008, JCYJ20200109144201725, KQTD20200909113758004, JSGG20200225151410198), the National Natural Science Foundation of China (82002140), and the Natural Science Foundation of Guangdong Province of China (2019A1515011197).

## Authors' Contributions

Z.Z. and L.W. designed this study and wrote the manuscript. H.Y. performed this study and wrote the manuscript. L.C. performed the antibody neutralization test and wrote the manuscript. B.J. performed the ELISA test. G.X. performed the 10X single-cell V(D)J sequencing. Y.L. revised the manuscript. S.Z. contributed to the discussion .
