# Supplementary material for: SCIGA: Software for large-scale, single-cell immunoglobulin repertoire analysis
Source: Gigascience. 2021 Sep 28;10(9):giab050. doi: 10.1093/gigascience/giab050 (PMC8478610; doi:10.1093/gigascience/giab050)
Supplement: giab050_GIGA-D-20-00341_Original_Submission [file giab050_giga-d-20-00341_original_submission.pdf]

GigaScience

SCIGA: A software for large-scale, single-cell immunoglobulin repertories analysis

--Manuscript Draft--

**Manuscript Number:**	GIGA-D-20-00341
**Full Title:**	SCIGA: A software for large-scale, single-cell immunoglobulin repertories analysis
**Article Type:**	Technical Note
**Funding Information:**	Foundation for Distinguished Young Talents in Higher Education of Guangdong (82025022) — Dr. Zheng Zhang
**Abstract:**	10X single-cell V(D)J sequencing enables the determining of B cell immunoglobulin repertoires with paired heavy- and light- chain. Precisely and quickly analyzing 10X single-cell immunoglobulin repertoires remains a challenge, due to the high diversity of immunoglobulin repertoires and the lack of specialized software that can analyze such diverse data. In this study, the SCIGA (https://github.com/sciensic/SCIGA), a specialized software for 10X single-cell immunoglobulin repertoires analysis, was developed. SCIGA is an easy-to-use pipeline that performs reads trimming, immunoglobulin sequence assembling and annotating, heavy- and light- chain pairing, statistical analysis, visualization and integrating multiple samples, all by using one line command. SCIGA was then used to profile the single-cell immunoglobulin repertoires of nine coronavirus disease 2019 (COVID-19) patients. From these repertoires, four neutralizing antibodies against severe acute respiratory syndrome coronavirus 2 (SARS-CoV-2) were identified.
**Corresponding Author:**	Zheng Zhang<br>Southern University of Science and Technology<br>Shenzhen, CHINA
**Corresponding Author Secondary Information:**	
**Corresponding Author's Institution:**	Southern University of Science and Technology
**Corresponding Author's Secondary Institution:**	
**First Author:**	Zheng Zhang
**First Author Secondary Information:**	
**Order of Authors:**	Zheng Zhang<br>Haocheng Ye<br>Lin Cheng<br>Bin Ju<br>Gang Xu<br>Yang Liu<br>Lifei Wang
**Order of Authors Secondary Information:**	
**Additional Information:**	
**Question**	**Response**
Are you submitting this manuscript to a special series or article collection?	No
**Experimental design and statistics**	Yes

Powered by Editorial Manager® and ProduXion Manager® from Aries Systems Corporation

Full details of the experimental design and statistical methods used should be given in the Methods section, as detailed in our Minimum Standards Reporting Checklist. Information essential to interpreting the data presented should be made available in the figure legends.<br><br>Have you included all the information requested in your manuscript?	
**Resources**<br><br>A description of all resources used, including antibodies, cell lines, animals and software tools, with enough information to allow them to be uniquely identified, should be included in the Methods section. Authors are strongly encouraged to cite Research Resource Identifiers (RRIDs) for antibodies, model organisms and tools, where possible.<br><br>Have you included the information requested as detailed in our Minimum Standards Reporting Checklist?	Yes
**Availability of data and materials**<br><br>All datasets and code on which the conclusions of the paper rely must be either included in your submission or deposited in publicly available repositories (where available and ethically appropriate), referencing such data using a unique identifier in the references and in the “Availability of Data and Materials” section of your manuscript.<br><br>Have you have met the above requirement as detailed in our Minimum Standards Reporting Checklist?	Yes

Manuscript Click here to access/download;Manuscript;manuscript.pdf

SCIGA: A software for large-scale, single-cell immunoglobulin repertoires analysis

Haocheng Ye[1,3#], Lin Cheng[1#], Bin Ju[1], Gang Xu[1], Yang Liu[1], Lifei Wang[2*], Zheng Zhang[1*]

[1]Institute for Hepatology, National Clinical Research Center for Infectious Disease, Shenzhen Third People's Hospital, The Second Affiliated Hospital, School of Medicine, Southern University of Science and Technology, Shenzhen, Guangdong 518112, China.

[2]Department of Radiology, National Clinical Research Center for Infectious Disease, Shenzhen Third People's Hospital, The Second Affiliated Hospital, School of Medicine, Southern University of Science and Technology, Shenzhen, Guangdong 518112, China.

[3]CAS Key Laboratory of Pathogenic Microbiology and Immunology, Institute of Microbiology, Chinese Academy of Sciences (CAS), Beijing, 100101, China

[#]These authors contributed equally.

***Correspondence:**

Zheng Zhang. Institute of Hepatology, Shenzhen 3rd People's Hospital, Shenzhen, Guangdong Province 518100, China; Email: zhangzheng1975@aliyun.com.

Lifei Wang. Department of Radiology, Shenzhen 3rd People's Hospital, Shenzhen, Guangdong Province 518100, China; Email: wanglf007n@163.com.

Abstract

10X single-cell V(D)J sequencing enables the determining of B cell immunoglobulin repertoires with paired heavy- and light- chain. Precisely and quickly analyzing 10X single-cell immunoglobulin repertoires remains a challenge, due to the high diversity of immunoglobulin repertoires and the lack of specialized software that can analyze such diverse data. In this study, the SCIGA (https://github.com/sciensic/SCIGA), a specialized software for 10X single-cell immunoglobulin repertoires analysis, was developed. SCIGA is an easy-to-use pipeline that performs reads trimming, immunoglobulin sequence assembling and annotating, heavy- and light- chain pairing, statistical analysis, visualization and integrating multiple samples, all by using one line command. SCIGA was then used to profile the single-cell immunoglobulin repertoires of nine coronavirus disease 2019 (COVID-19) patients. From these repertoires, four neutralizing antibodies against severe acute respiratory syndrome coronavirus 2 (SARS-CoV-2) were identified.

Key words: Software; Single-cell; Immunoglobulin repertoires; COVID-19; Antibody

Introduction

B cell immunoglobulin diversity is an important characteristic of the adaptive immune system and developed through the rearrangement of variable V,

(diversity D), and the joining of J gene segments, the pairing of heavy- and light- chains, and somatic hypermutation (SHM) [1]. Exposure to infections and environmental factors potentially shape the B cell immunoglobulin repertoires [2-4], and can lead to clonal expansion of the immune cells, allowing them to change into different types in order to respond to a specific antigen. Understanding these immunoglobulin repertoires can help researchers to discover antibodies, monitor vaccination responses and infer B cell trafficking patterns [5, 6].

10X single-cell V(D)J sequencing is a powerful tool for investigating paired heavy- and light- chain repertoires of B cell immunoglobulin [7]. It has been used in the identification of neutralizing antibodies against severe acute respiratory syndrome coronavirus 2 (SARS-CoV-2) [8], the virus causing coronavirus disease 2019 (COVID-19) [9]. However, accurately analyzing 10X single-cell immunoglobulin repertoires remains a challenge, due to the high diversity of immunoglobulin repertoires and the lack of specialized software that can analyze such diverse data.

Here, we developed the SCIGA (Single-cell Immunoglobulin repertoires analysis), a software for quickly analyzing the data of 10X single-cell immunoglobulin repertoires. SCIGA performs reads trimming, immunoglobulin sequence assembly and annotation, heavy- and light- chain pairing by one line command. It also computes statistics of repertoires such as gene usage frequency, SHM rate, length of complementarity determining region 3 (CDR3)

and diversity and implements visualization. Using SCIGA, we then profiled the immunoglobulin repertoires of peripheral blood mononuclear cells (PBMCs) from nine COVID-19 patients and identified four neutralizing antibodies against SARS-Cov-2 from these repertoires.

Methods

SCIGA is a software for 10X single-cell immunoglobulin repertoires analysis. It integrates several tools and algorithms into a single workflow. The input data can be raw reads or output of Cellranger [10]. The details of the SCIGA algorithm can be found in the Supplementary Methods and Materials. Briefly, the workflow, summarized in Fig. 1, is as follows: 1) Reads quality control. Trim the reads of low-quality by Trimmomatic [11]; 2) Call cell. Since the 10X system generates a large of Gel Beads-in-emulsion (GEMs) containing no cell, SCIGA provides two methods to distinguish the GEMs containing B cell from all of GEMs based on reads number of GEMs; 3) Immunoglobulin sequence assembly. The immunoglobulin sequences for each cell were assembled by using SSAKE [12], which is a reliable de novo assembler for short reads; 4) Call gene. To detect the usage of the V(D)J gene and C gene, SCIGA aligns the assembled immunoglobulin sequence against the V-, D-, J- genes reference database by IgBLAST [13] and against C- gene reference database by BLAST [14]. The V(D)JC reference databases for humans, mice and rats have been downloaded from IMGT [15] and embedded in SCIGA; 5) Quality control of the

immunoglobulin sequence. Only the immunoglobulins which are complete, productive, in correct reading frame and have no stop codon are screened; 6) Quality control of the cells. After immunoglobulin sequence assembling and filtering, some cells have multiple heavy- or light- chains, while the other cells have only one chain. For each cell SCIGA reports the heavy- and light- chain with the highest number of unique molecular identifiers (UMIs) and a certainty score is calculated for each reported chain [7]. The chains with a certainty score less than a specified threshold are discarded, then the cells without pairing heavy- and light- chains are filtered out; 7) Clonal lineage grouping. Clonal lineage is defined as the cells which have identical V_H, J_H, V_L and J_L genes, identical H-CDR3 length, and over a similarity threshold of H-CDR3 nucleotide sequences [7]; 8) Statistical analysis and visualization. SCIGA automatically calculates a list of statistics including gene usage frequency, SHM rate, CDR3 length, Simpson index, Shannon entropy, etc. SCIGA then generates figures of the profile of repertoires. 9) Multiple samples integrating. After analyzing each sample, SCIGA consolidates all of outputs and identifies shared immunoglobulin sequences, which are potential public antibodies against a specific pathogen. Shared immunoglobulins are defined as immunoglobulins from different samples that can be clustered into the same clonal lineage. Clustering is performed as step 7 with cells from all samples.

Results

Comparing SCIGA to existing software

At time of publication, Cellranger is the only existing software for processing raw data generated by 10X single-cell V(D)J sequencing. To compare SCIGA to Cellranger, PBMCs from nine COVID-19 patients (B1 to B9) were collected and analyzed (Fig. 2A and Table S1). The raw data were respectively processed by SCIGA and Cellranger (v3.1.0) with default parameter. The comparison mainly focused on the following aspects: 1) Cell quality control. For Cellranger, the final results still included considerably low-quality cells, which either had multiple heavy- and light- chains, or had only one chain. In our test data set, the percentage of the low-quality cells reached an average of 29% (16.8% to 47.6% per sample, Fig. 2B). For SCIGA, it will implement the cell quality control process (step 6 of methods) and only output the passed high-quality cells. Due to the strict quality control process, cell count was generally less in SCIGA compared to Cellranger (Fig. 2C). 2) Detecting B cell clonal lineage. Cellranger clusters B cells into a clonal lineage when cells share identical nucleotide sequences of CDR3. However, it will break up clonotypes that are in fact clonally related when SHM fall within the CDR3. SCIGA uses a popular clonal definition (step 7 of methods) and it could detect larger clonal lineage than Cellranger (Fig. 2D). 3) Information of output. The output of Cellranger is quite limited and some important information, such as SHM rate, are not included. SCIGA computes all necessary statistics including gene frequency, clone

frequency, clone diversity, SHM rate, CDR3 length and the immunoglobulin variable region sequence (Fig. 2E). Moreover, SCIGA has visualization capabilities to show the global profile of repertoires. 4) Detecting shared immunoglobulin. It is a specific function in SCIGA and it could detect the shared immunoglobulin across samples.

Profiling immunoglobulin repertoires of COVID-19 by SCIGA

To show the usage and performance of SCIGA, an example study was launched here. Based on the results generated by SCIGA, we profiled the immunoglobulin repertoires of the nine COVID-19 patients. A total of 8,358 B cells were detected (571 to 2,371 cells per sample, Fig. 2C). For V genes, we focused on genes used in at least 1% of cells (Fig. 3A and Fig. S1). The top three gene families were IGHV4-34 (12.51%), IGHV3-30 (7.95%) and IGHV3-23 (6.30%) for heavy chain and IGLV3-19 (9.46%), IGKV1-39 (8.26%) and IGKV3-20 (7.90%) for light chain. Among them, IGHV4-34 and IGLV3-19 have pretty high usage in patient B2, which reach 63.98% and 64.39%, respectively. For C gene (isotype), IGHM had the highest frequency in most of patients except patient B2, who preferred to use IGHG1 (Fig. 3B). Most patients had low mean SHM rates (<2%, Fig. 3C and Fig. S2). However, patient B2 had a high SHM rate for IGH chain (7.48%) and IGL chain (7.13%). All patients had similar mean CDR3 length of IGH, IGK and IGL chain except for the longer CDR3 length of IGH chain in patient B2 (Fig. 3D and Fig. S3).

Clonal lineage analysis by SCIGA

Clonal lineages were defined by SCIGA with default parameter. We used Simpson index and Shannon entropy computed by SCIGA to determine the clonality of immunoglobulin repertoires (Fig. 4A and 4B). Both indexes showed that patient B1 and patient B2 had high clonality, indicating that they may have large clones. The top 10 largest clones of each patient (Fig. 4C and Fig. S4) were reviewed. Some patients such as patient B2 and patient B5 had one dominant clone, while other patients such as patient B1 had even clone frequency. The largest clone (61.21%) in patient B2 belonged to the IGHV4-34 and IGLV3-19 family, with an 8.82% mean SHM rate and 23-amino H-CDR3 length. The top 10 largest clones for all of patient preferred to use IGHV4-34 (9 clones) as heavy chain, IGKV1-39 (9 clones) and IGKV3-20 (9 clones) as light chain and IGHV4-34: IGLV3-19 (5 clones) as pairing (Fig. S5A and S5B and S5C). We next determined the shared immunoglobulin sequences across patients by the integration function of SCIGA. There are 12, 1 and 26 immunoglobulins were respectively sharing between patient B1 and patient B2, patient B5 and patient B8 as well as patient B6 and patient B9 (Fig. 4D and Table S2).

Identification of neutralizing monoclonal antibodies

It was hypothesized that IgGs with higher clonal expansion may be SARS-CoV-2-specific antibodies in the COVID-19 patients. Thus, monoclonal antibodies (mAbs) were screened for following criteria: IgG clone with fraction >= 1% and cell number >= 20. We selected and expressed four typical mAbs: B2-C1, B6-

C2, B6-C3, and B8-C1. Remarkably, by ELISA, all four mAbs were SARS-CoV-2 RBD (receptor binding domain) specific antibodies, which bound to the extracellular domain (ECD), S1 subunit, and RBD of the SARS-CoV-2 spike, not to N-terminal domain (NTD) and S2 subunit (Fig. 5A). Monoclonal antibodies could neutralize SARS-CoV-2 by blocking the attachment of RBD to the receptor (angiotensin-converting enzyme 2, ACE2) on host cells. B2-C1 and B6-C3 exhibited potent neutralizing activity [half-maximal inhibitory concentration (IC_{50}) = 0.75 μg/ml and 0.32 μg/ml, respectively] against SARS-CoV-2 pseudovirus, whereas B8-C1 (1.47 μg/ml) and B6-C2 (14.89 μg/ml) were moderate and weak neutralizing antibodies (Fig. 5B). Similar results were found in the neutralization of the four mAbs against SARS-CoV-2 live virus (Fig. 5C).

Discussion

In this study, we developed the SCIGA pipeline for 10X-based single-cell immunoglobulin repertoires analysis. It is an easy-to-use software and allows researchers to quickly perform advanced analysis on 10X V(D)J sequencing datasets. Although Cellranger has also previously been used for 10X single-cell immunoglobulin repertoires analysis, it is not as effective, including low-quality cells and disregarding the effect of SHM when defining clonal lineage. Also, some important information of immunoglobulin repertoires, such as SHM rate, is not included in the results of Cellranger. SCIGA performs a cell quality control process and defines clonal lineage including the effect of SHM. Larger clones

can be detected by SCIGA. Moreover, SCIGA provides a number of needed statistical parameters and is a more efficacious tool for researchers.

SCIGA was used to analyze the single-cell immunoglobulin repertoires of COVID-19 patients. Large scale clone expansion was not observed in most patients. In patient B2, however, B cells expanded, indicated by a large clonal lineage within the IgG isotype. This indicates that patient B2 potentially generated neutralizing antibodies against SARS-CoV-2. Additionally, IGHV4-34 was found to be the most frequent heavy chain among patients, which may be associated with SARS-CoV-2 infection.

Finally, SARS-CoV-2 responding antibodies were defined using several criteria. Previously, immunoglobulin with an SHM rate lower than 2% were excluded [8]. However, since several potent neutralizing antibodies against SARS-CoV-2 have low SHM rates [16-18], we included this in our definition. Our data also support the definition that the larger clone size is not always accompanied with higher SHM rate (Figure 4C). Totally, four neutralizing antibodies with different potency were identified. This demonstrates that SCIGA is useful for 10X single-cell immunoglobulin repertoires analysis.

List of abbreviations

ACE2: angiotensin-converting enzyme 2

CDR3: complementarity determining region 3

COVID-19: coronavirus disease 2019

ECD: extracellular domain

GEMs: Gel Beads-in-emulsion

IC50: half-maximal inhibitory concentration

NTD: N-terminal domain

PBMCs: peripheral blood mononuclear cells

SARS-CoV-2: severe acute respiratory syndrome coronavirus 2

SHM: somatic hypermutation

Availability of supporting source code and requirements

Project name: SCIGA

Project home page: https://github.com/sciensic/SCIGA

Operating system(s): Linux

Programming language: Perl

Other requirements: IgBlast 1.15.0 or higher, Blast 2.9.0 or higher, R (optional), ggplot2 (optional)

License: GNU GPL-3.0 License

Availability of supporting data

The data set(s) supporting the results of this article is(are) available in the [National Center for Biotechnology Information] repository (https://www.ncbi.nlm.nih.gov/), [PRJNA682839].

Ethics, consent and permissions

This study was conducted according to the ethical principles of the Declaration of Helsinki. Ethical approval was obtained from the Research Ethics Committee of Shenzhen Third People's Hospital (2020-207). All participants provided written informed consent for sample collection and subsequent analyses.

Authors' contributions

Z.Z. designed this study and wrote the manuscript. H.Y. performed this study and wrote the manuscript. L.C. performed the antibody neutralization test and wrote the manuscript. B.J. performed the ELISA test. G.X. performed the 10X single-cell V(D)J sequencing. Y.L. revised the manuscript.

Competing interests

The authors declare that they have no competing interests.

Funding

This study was supported by the National Science Fund for Distinguished Young Scholars (82025022), the Sanming Project for Medicine of Shenzhen (SZSM201612053), the National Key Plan for Scientific Research and Development of China (2020YFC0848800, 2020YFC0844200), the National Science and Technology Major Project of the Infectious Diseases (2018ZX10301404 to ZZ), the Science and Technology Innovation Committee

of Shenzhen Municipality (202002073000002, 2020A1111350032, JCYJ20190809115617365), the National Natural Science Foundation of China (82002140) and the Natural Science Foundation of Guangdong Province of China (2019A1515011197).

Acknowledgements

Not applicable

References

1. V, G., et al., - *Bioinformatic and Statistical Analysis of Adaptive Immune Repertoires.* - Trends Immunol. 2015 Nov;36(11):738-749. doi: 10.1016/j.it.2015.09.006. Epub 2015, (- 1471-4981 (Electronic)): p. - 738-749.
2. FA, T., et al., - *Biased IGH VDJ gene repertoire and clonal expansions in B cells of chronically.* - Blood. 2018 Feb 1;131(5):546-557. doi: 10.1182/blood-2017-09-805762. Epub 2017 Dec, (- 1528-0020 (Electronic)): p. - 546-557.
3. SCA, N., et al., - *Shaping of infant B cell receptor repertoires by environmental factors and.* - Sci Transl Med. 2019 Feb 27;11(481):eaat2004. doi: 10.1126/scitranslmed.aat2004., (- 1946-6242 (Electronic)): p. T - ppublish.
4. A, N., et al., - *Fierce Selection and Interference in B-Cell Repertoire Response to Chronic HIV-1.* - Mol Biol Evol. 2019 Oct 1;36(10):2184-2194. doi: 10.1093/molbev/msz143., (- 1537-1719 (Electronic)): p. - 2184-2194.
5. H, R., - *Immunosequencing: applications of immune repertoire deep sequencing.* - Curr Opin Immunol. 2013 Oct;25(5):646-52. doi: 10.1016/j.coi.2013.09.017. Epub 2013, (- 1879-0372 (Electronic)): p. - 646-52.
6. JN, S., et al., - *B cells populating the multiple sclerosis brain mature in the draining cervical.* - Sci Transl Med. 2014 Aug 6;6(248):248ra107. doi: 10.1126/scitranslmed.3008879., (- 1946-6242 (Electronic)): p. - 248ra107.
7. LD, G., et al., - *Massively parallel single-cell B-cell receptor sequencing enables rapid discovery of.* - Commun Biol. 2019 Aug 9;2:304. doi: 10.1038/s42003-019-0551-y. eCollection 2019., (- 2399-3642 (Electronic)): p. - 304.
8. Y, C., et al., - *Potent Neutralizing Antibodies against SARS-CoV-2 Identified by High-Throughput.* - Cell. 2020 Jul 9;182(1):73-84.e16. doi: 10.1016/j.cell.2020.05.025. Epub 2020 May, (- 1097-4172 (Electronic)): p. - 73-84.e16.
9. D, W., et al., - *The SARS-CoV-2 outbreak: What we know.* - Int J Infect Dis. 2020 May;94:44-48. doi: 10.1016/j.ijid.2020.03.004. Epub 2020 Mar, (- 1878-3511 (Electronic)):

p. - 44-48.
10. group, X.g.; Available from: https://support.10xgenomics.com/single-cell-gene-expression/software/downloads/latest?
11. AM, B., L. M, and U. B, - *Trimmomatic: a flexible trimmer for Illumina sequence data.* - Bioinformatics. 2014 Aug 1;30(15):2114-20. doi: 10.1093/bioinformatics/btu170. Epub, (- 1367-4811 (Electronic)): p. - 2114-20.
12. RL, W., et al., - *Assembling millions of short DNA sequences using SSAKE.* - Bioinformatics. 2007 Feb 15;23(4):500-1. doi: 10.1093/bioinformatics/btl629. Epub, (- 1367-4811 (Electronic)): p. - 500-1.
13. J, Y., et al., - *IgBLAST: an immunoglobulin variable domain sequence analysis tool.* - Nucleic Acids Res. 2013 Jul;41(Web Server issue):W34-40. doi: 10.1093/nar/gkt382., (- 1362-4962 (Electronic)): p. - W34-40.
14. C, C., et al., - *BLAST+: architecture and applications.* - BMC Bioinformatics. 2009 Dec 15;10:421. doi: 10.1186/1471-2105-10-421., (- 1471-2105 (Electronic)): p. - 421.
15. MP, L., et al., - *IMGT®, the international ImMunoGeneTics information system® 25 years on.* - Nucleic Acids Res. 2015 Jan;43(Database issue):D413-22. doi: 10.1093/nar/gku1056., (- 1362-4962 (Electronic)): p. - D413-22.
16. C, K., et al., - *Longitudinal Isolation of Potent Near-Germline SARS-CoV-2-Neutralizing Antibodies.* - Cell. 2020 Aug 20;182(4):843-854.e12. doi: 10.1016/j.cell.2020.06.044. Epub 2020 Jul, (- 1097-4172 (Electronic)): p. - 843-854.e12.
17. B, J., et al., - *Human neutralizing antibodies elicited by SARS-CoV-2 infection.* - Nature. 2020 Aug;584(7819):115-119. doi: 10.1038/s41586-020-2380-z. Epub 2020 May, (- 1476-4687 (Electronic)): p. - 115-119.
18. TF, R., et al., - *Isolation of potent SARS-CoV-2 neutralizing antibodies and protection from disease.* - Science. 2020 Aug 21;369(6506):956-963. doi: 10.1126/science.abc7520. Epub 2020 Jun, (- 1095-9203 (Electronic)): p. - 956-963.
19. I, L., et al., - *BraCeR: B-cell-receptor reconstruction and clonality inference from single-cell.* - Nat Methods. 2018 Aug;15(8):563-565. doi: 10.1038/s41592-018-0082-3., (- 1548-7105 (Electronic)): p. - 563-565.
20. N, C. and W. DR, - *Analyzing Immunoglobulin Repertoires.* - Front Immunol. 2018 Mar 14;9:462. doi: 10.3389/fimmu.2018.00462. eCollection 2018., (- 1664-3224 (Print)): p. - 462.
21. L, Z., et al., - *Lineage tracking reveals dynamic relationships of T cells in colorectal cancer.* - Nature. 2018 Dec;564(7735):268-272. doi: 10.1038/s41586-018-0694-x. Epub 2018 Oct, (- 1476-4687 (Electronic)): p. - 268-272.

Figure legend

Fig. 1 SCIGA workflow. The workflow includes reads quality control, calling cell from the background, immunoglobulin (Ig) sequence assembly, calling V(D)JC gene of Ig, Ig sequence quality control, cell quality control, clonal lineage grouping, statistical analysis and visualization, multiple sample integration.

Fig. 2 Comparison of SCIGA and Cellranger. (A) Flowchart of the experiment. **(B)** The percentage of low-quality cells processed by Cellranger. Single: the cells containing single chain. Multiple: the cells containing multiple heavy- or light- chains. **(C)** B Cell count processed by SCIGA and Cellranger. **(D)** The top 10 largest clone analyzed by SCIGA and Cellranger. **(E)** Output SCIGA and Cellranger.

Fig. 3 Profile of single-cell immunoglobulin repertoires of nine COVID-19 patients. (A) From left to right, the average frequency of use of the V genes of IGH, IGK and IGL. Only the genes with a frequency > 1% are shown. **(B)** The frequency of isotypes in patients. Color denotes the isotype. **(C)** Mean SHM rate of V genes **(D)** Mean CDR3 length in each patient, with IGH shown in red, IGK in yellow and IGL in blue. The error bars represent the standard error.

Fig. 4 B cells clone expansion in nine COVID-19 patients. (A) Simpson

index denoting clonality. **(B)** Shannon entropy denoting clonality. **(C)** The top 10 largest clones in each patient. The X axis captures the clone ID and V genes of heavy- and light- chains. The initial of the gene name denotes the chain, H: IGH, K: IGK, L: IGL **(D)** Number of immunoglobulins between patients. Blank means 0.

Fig. 5 Characteristics of spike specific monoclonal antibodies. (A) The binding profile of selected monoclonal antibodies to the extracellular domain and subdomains of the SARS-CoV-2 spike by ELISA. HIV-1-GP140 is the negative control. **(B-C)** Neutralization activity of selected monoclonal antibodies against the pseudovirus (**B**) and live SARS-CoV-2 (**C**). The dashed line indicates a 50% reduction in viral infectivity. Human IgG1 is the negative control. Results presented here are representative of two independent experiments.

Fig. S1 Frequency of V genes in each patient. Only the genes with a frequency of >1% are included.

Fig. S2 Distribution of the SHM rate in each patient. Color denotes the chain, with IGH shown in red, IGK in yellow and IGL in blue.

Fig. S3 Distribution of the CDR3 length in each patient. Color denotes the chain, with IGH shown in red, IGK in yellow and IGL in blue.

Fig. S4 Fraction of all clone in each patient. X-axis is the clone rank in the form of logarithm. The Y-axis is clone fraction.

Fig. S5 Number of genes in the top 10 largest clones of all patients. (A) Gene count in the heavy chain. **(B)** Gene count in the light chain. **(C)** Gene count in the pairing heavy- and light- chain. The initial of the gene name denotes the chain, H: IGH, K: IGK, L: IGL.

Fig. S6 Two different examples of barcodes rank corresponding to their reads count. The example having **(A)** and not having **(B)** large difference of reads count between cells-containing GEMs and background.

Table S1 Information of the nine COVID-19 patients.

Table S2 Information of the shared immunoglobulins. The number in column 7 to 11 denotes the number of immunoglobulin sequences clustering to the clone.

Supplementary Methods and Materials

Note 1. Algorithm of SCIGA

Note 2. Identification of biological activity of the monoclonal antibody

Note 3. Nucleotide sequences of antibodies against SARS-CoV-2

Note 1. Full algorithm of SCIGA

Reads quality control

SCIGA trims the reads of low-quality by using Trimmomatic (embedded in SCIGA). SCIGA allows user to set up the quality control criterion, including the sliding window size for trimming reads (default is 4), setting the cutoff of average quality score in a sliding window (default is 15) and setting the cutoff of length for reads after trimming (default is 75).

Cell calling

In the 10X system, the majority (~90 – 99%) of generated Gel Beads-in-emulsion (GEMs) contain no cell and we need to detect the GEMs containing cells depend on the count of V(D)J transcripts. SCIGA trims the first 39 bases of read 1 containing the 16 nt cell barcode, 10 nt unique molecular identifier (UMI) and 13 nt switch oligo as previous study[7]. The barcode and UMI are retained for each read. Reads with identical cell barcode are considered as deriving from the same cell. SCIGA calculates the number of reads per barcode and ranks them in reverse order. The barcodes out of top 10% are discarded

since at least 90% GEMs contains no cell. Then SCIGA provides two methods for detecting “real” cells from all GEMs: 1) SCIGA constructs a curve graph of rank of barcodes corresponding to their reads count in the form of a logarithm. The point at which the gradient is minimal is set as the cutoff point and barcodes with more reads than cutoff point are considered as “real” cells. This method is suitable for samples having large difference of reads count between “real” cells and background (Fig. S6A). 2) Barcodes are considered as “real” cells when total number of reads exceed an arbitrary cutoff set by user (default is 200) (Fig. S6B).

Immunoglobulin sequence assembly

If reads for a given barcode exceeded 80,000, they were downsampled to 80,000 before assembling. Then SCIGA performs immunoglobulin assembly for each barcode separately by using SSAKE (embedded in SCIGA), which is a reliable de novo assembler for short reads. The trimmed reads 1 and reads 2 are as input for SSAKE with the parameter “-w 5 -p 1 -c 1”. The contig with length less than 300 bases or coverage less than a threshold (default is 3) is discarded.

Making a reference database

Before annotating the assembled immunoglobulin sequence, a reference database (embedded in SCIGA) was needed. We downloaded ungapped nucleotide sequences of all V-, D-, J- and C-gene segments of heavy, kappa and lambda chains of certain species (containing human, mouse, and rat)

from The International ImMunoGeneTics information system (IMGT, http://www.imgt.org). Some C-gene reference have the same gene name, we thus added a different number to the same gene name to distinguished them. The V-, D-, J- and C- sequences are separately used to build IgBLAST database and BLAST database.

V(D)JC gene calling

To detect the use of the V(D)J gene, SCIGA aligns the passed contigs against the V-, D-, J- gene reference database by IgBLAST with parameter "-evalue 0.001". To detect the isotype, SCIGA aligns the contigs against the C-gene reference database by BLAST with parameter "-evalue 0.001". SCIGA only retains the alignments that have the highest score.

Immunoglobulin sequence quality control

To obtain a complete and productive V(D)J sequence, SCIGA sets up several quality control steps based on the IgBLAST output. First, the V(D)J sequences that can not be assigned to certain V- or J- genes are discarded. Second, V(D)J sequences that can not be identified by CDR3 region are also discarded. Third, V(D)J sequences should be productive, in correct reading frame, and have no stop codon. Fourth, to ensure the intactness of the FR1 region, V(D)J nucleotide sequences should align to the first position of the V-gene. Fifth, CDR3 region is considered complete if the V(D)J amino sequence contained the first four positions of the FR4 region, as previously defined [19]. The first four positions should be the conserved motif of XGXG, WSQG

(heavy chain), FGXG (light chains) or FSDG (kappa chain).

B cell quality control

In general, a B cell has one heavy- and one light- chain. However, after immunoglobulin sequence assembly and quality control, some cells have multiple heavy- or light- chains (this may be due to the contamination of free RNA or multiple cells in a GEMs), while the other cells have only one chain (this may be due to the low sequencing depth). For each cell, SCIGA reports the heavy- and light- chain with the highest UMIs number. Then for each reported chain SCIGA calculates a certainty score, defined as the number of UMIs supporting the chain divided by the total number of UMIs for all heavy- and light-chains, respectively. The chains with a certainty score less than a threshold (default is 80%) are discarded. Finally, the cells without pairing heavy- and light- chains are filtered out.

Clonal lineage grouping

SCIGA defines cells as clonal lineage when the cells have identical V_H, J_H, V_L and J_L genes, identical H-CDR3 length, and over a similarity threshold of H-CDR3 nucleotide sequences [7]. SCIGA implements this by using a custom script: 1) Grouping the cells which have identical V_H, J_H, V_L and J_L genes and identical H-CDR3 length into a cluster. 2) For each cluster, SCIGA merges the identical H-CDR3 nucleotide sequences into unique sequence and calculates count of the unique sequence, then ranks the unique sequences by count in reverse order. 3) Performing an iteration process: the first sequence is as the

representative of the first clone. Then a given sequence is compared to the representative of each clone in turn and identity scores are calculated. If the maximum identity score is more than a similarity threshold set by user, SCIGA assigns the given sequence to the clone corresponding to the maximum identity score, or assigns it to a new clone.

Statistical analysis and visualization

SCIGA automatically computes a list of statistics. Some of them are calculated as below:

Gene usage frequency is calculated as $\frac{gene\ usage\ count}{total\ cell\ count} \times 100\%$.

SHM rate of the V(D)J gene is calculated as $\frac{mismatchs\ in\ gene}{gene\ length} \times 100\%$.

Simpson index is calculated as $\frac{\sum_{i=1}^{S} n_i(n_i-1)}{N(N-1)}$, where n_i is the number of cells of the ith clone and N is the total number of cells, and S is the total number of clones[20].

Shannon entropy is calculated as $1 - \frac{-\sum_{i=1}^{S} p_i log_2 p_i}{log_2 S}$, where p_i is the fraction of the ith clone and S is the total number of clones[21].

Moreover, SCIGA has the optional function of drawing the figures including distribution of gene usage frequency, SHM of V gene, CDR3 length and clone frequency by using R.

Multiple samples integration analysis

After analyzing of each sample, SCIGA integrates the outputs of several samples into one and tries to find the shared immunoglobulin sequences. Shared immunoglobulins are defined as immunoglobulins from different

samples that can be clustered into same clonal lineage. Clustering is performed with cells of all samples.

Basic use of SCIGA for each sample:

sciga -fq1 <mate1.fastq.gz> -fq2 <mate2.fastq.gz> -outdir <output> -species <species> -draw T

Integrating outputs of multiple samples:

sciga-merge -in <sample1, sample2, sample3…> -out <output>

Note 2. Identification of biological activity of monoclonal antibody

Enzyme-linked immunosorbent assay

The recombinant extracellular domain or other subdomains of SARS-CoV-2 S protein (spike, S1, RBD, NTD, and S2, all from Sino Biological, Beijing) was coated (2 μg/ml) into 96-well plates overnight at 4°C. The plates were blocked with the blocking buffer (PBS containing 5% skim milk and 2% bovine albumin) at RT for 1h. Five-fold serial-diluted mAbs were added into the plates and then incubated for 1 hour at 37°C. HRP-conjugated Goat anti-Human IgG (ZSGB-BIO, Beijing) secondary antibody was added into the plates and incubated at 37°C for 1 hour. The enzymatic reaction was developed with TMB substrate (Kinghawk, Beijing) and stopped by 2M H_2SO_4. The optical density was measured at 450 nm (OD 450nm) with a Varioskan™ LUX Multimode Microplate Reader (Thermo Scientific). HIV-1-GP140 (purified in our lab) was an irrelevant antigen control.

Pseudovirus-based neutralization assay

The SARS-CoV-2 pseudovirus was generated through co-transfection of 293T cells with pVAX1-S and pNL4-3.Luc.R-E-, carrying the codon optimized SARS-CoV-2 S gene (GenBank: MN988668.1) and HIV-1 backbone, respectively. Viral supernatant was collected at 48 hours post-transfection and frozen at -80°C. The serially diluted antibodies were incubated with equal volume pseudovirus at 37°C for 1 hour. The antibody-virus mixtures were subsequently added into 96-well plates which pre-seeded HEK 293T-ACE2 cells. After 48 hours, infected cells were lysed to measure the luciferase activity using Bright-Glo Luciferase (Promega, Madison, WI) according to the manufacturer's protocol. The 50% inhibitory concentration (IC_{50}) was determined by GraphPad Prism 7 using asymmetric (five parameter) model.

Focus reduction neutralization test

SARS-CoV-2 focus reduction neutralization test (FRNT) was performed in a certified Biosafety level 3 lab. Antibodies were 3-fold serially diluted and mixed with equal volume of SARS-CoV-2 live virus (containing 200 focus forming unit) in U-bottom 96-well plates. The mixtures were incubated for 60 min at 37 ^{o}C and then transferred into the 96-well plate seeded with Vero E6 cells for 1 hour at 37 ^{o}C before removed. After washing, the overlay media (MEM containing 1.6% Carboxymethylcellulose, 2% fetal bovine serum) was added and then cells were incubated at 37 ^{o}C for 24 hours. After removing the overlay media, cells were fixed with 4% paraformaldehyde solution, permeabilized with Perm/Wash buffer (BD Biosciences) containing 0.1% Triton X-100, incubated with HRP-conjugated anti-SARS-CoV-2-N IgG (isolated in our lab). The reactions were developed with KPL TrueBlue Peroxidase substrates (Seracare

Life Sciences Inc). The numbers of SARS-CoV-2 foci were calculated using an EliSpot reader (Cellular Technology Ltd).

Note 3. Nucleotide sequences of antibodies against SARS-CoV-2

Paired heavy- and light- chains of B cells are alternately shown as below:

>B2-C1_IGH

CAGGTGCAGCTACAGCAGTGGGGCGCGGGACTGTTGAAGCCTTCGGAGACCCTGT
CCCTCACCTGCGCTGTCTATGGTGTGTCGCCCACTAGTTACTATTGGAGCTGGATCC
GTCAGTCCCCCGGGAAGGGTCTGGAGTGGATAGGGGAGATCACTCATAGTGGAAGC
ACCAACTACAATCCGTCCCTCAAGAGTCGAGTCACCATGTCGCTGGACACGTCCAA
GAGCCAGTTCTCCCTGAAGTTGAGTTCTGTGACCGCCGCGGACACGGCTATATATTA
TTGTGCGAGGGGACGCAGTGAGGAGACCATGATAGTGATGGTTGTCACGGGAATTG
ATTTCTACTTTGACTCTTGGGGCCAGGGGACCCTGGTCACCGTCTCCTCA

>**B2-C1_IGL**

TCTTCTGAGCTGACTCAGGACCCTGCTGTGTCTGTGGCCTTGGGACAGACAGTCAG
GATCACATGCCAAGGAGACAACCTCAAACTCTTTTATACAAACTGGTACCAGCAGAA
GCCAGGCCAGGCCCCCGTACTTGTCATCCATGGTAAAAACAACCGGCCCTCAGGGA
TCCCAGACCGATTCTCTGGCTCCAGTTCAGCGTACACCACTTCCTTGACCATCATTG
GGGCTCAGGCGGAGGATGAGGCTGACTATTACTGTAGCTCTCGCGACAGAAGTGGT
GACCGTGTTATATTCGGCGGAGGGACCAAGGTGACTGTCCTA

>B6-C2_IGH

GAGGTGCTCCTGGTGGAGTCTGGGGGAGGCTTGGTCCGGCCTGGAGGGTCCCTAA

GACTCTCCTGTGCAGCCTCTGGATTCACCTTCACTGACCACTATTTGGACTGGGTCC
GCCAGGCTCCAGGGATGGGGCTGGAGTGGGTTGGCCGTATTAGAAATAAAGTTAAT
GGTTACACCACAGAATACGCCGCGTCTGTGAAAGGCAGATTCACCATCTCAAGAGAT
GATTCAAAGAACTCAGTTTATCTGCAAATGAATAGCCTGAGAAGCGAGGACACGGCC
GTGTATTACTGCACTAGAGTGGGAGTTGGGAGCCCTGACTACTGGGGCCAGGGAAC
CCTGGTCGCCGTCTCCTCA

>B6-C2_IGK

GACATCCAGATGACCCAGTCTCCATCCTCCCTGTCTGCATCTGTAGGAGACAGAGTC
ACCATCACTTGCCGGGCAAGTCAGGGCATTAGAGATGAGTTAGCCTGGTATCAGCAA
AAACCAGGGAAAGCCCCTAAGCGCCTGATCTATGATGCATCGAGGTTGCAAAGTGG
GATCCCATCGAGGTTCAGCGGCAGTGGATCTGGGACAGAATTCACTCTCACAATCAG
CAGTCTGCAGCCTGAAGATTTTGCAACTTATCATTGTCTACAGTATACTAGTTACCCTC
ACACTTTTGGCCAGGGGACCAAGCTGGACATCAAA

>B6-C3_IGH

CAGGTGCAGCTACAACAGTGGGGCGCAGGACTGTTGAAGCCTTCGGAGACCCTGT
CCCTCACCTGCGCTGTCTATGGTGGGTCCTTCAGTGGTTACCAGTGGAGGTGGATC
CGCCAGGCCCCAGGGAAGGGGCTGGAGTGGATTGGGGAAATCAATCATAGTGGAA
GCACCAATTACAACCCGTCCCTCAAGAGTCGAGTCACCATATCAGTAGACACGTCCA
AGAACCAGTTCTCCCTGAGGTTGAGGTCTGTGACCGCCGCGGACACGTCTGTGTAT
TTCTGTGCGAGAGGCCAAAATGGAGTAGTTCCAGCTCCTGTATTGGGGATCGGACCT
TACTACACCTACTCCTACATGGACGTCTGGGGCACAGGGACCACGGTCAGTGTCTC
CTCA

>B6-C3_IGL

TCTTCTGAGCTGACTCAGGACCCTGCTGTGTCTGTGGCCTTGGGACAGACAGTCAGGATCACATGCCAAGGAGACAGCCTCAGAAGCTATTATGCAAGTTGGTACCAGCAGAAGCCAAGACAGGCCCCTATTCTTGTCATCTATGGTAAAAACAATCGACCCTCAGGGATCCCGGACCGATTCTCTGGCTCCTACTCAGGAGCCACAGCTTCCTTAACCATCACTGGGGCTCAGGCGGAGGATGAGGCTGACTATTATTGTGACTCCCGGGACAGCAGTGGTAACCATCGAGTGTTCGGCAGAGGGACCACGGTGACCGTGCTA

>B8-C1_IGH

CAGGTGCAACTGGTGCAGTCTGGGGCTGAGGTGAAGAAGCCTGGGTCCTCGGTGAGGGTCTCCTGCCAGGCTTCTGGAGACACCTTCAGCAACTATGCTTTCAGTTGGGTGCGACAGGCCCCTGGACAAGGGCTTGAGTGGATGGGAAGGATCATCCCTATCTTTGGAACACCAAACTACGCACAGAGGTTCCAGGGGAGAGTCACGATTACCGCGGACGAGTCTACGAGGACAGCCTACATGGAATTGACCGGCCTGAGGTCTGACGACACGGCCGTGTATTACTGTGCGAGACACACTTTGGTGACTGCTATTCAGAAGTGGGGCCAGGGAACCCTGGTCACCGTCTCCTCA

>B8-C1_IGK

GACATCCAGATGACCCAGTCTCCTTCCACCCTGTCTGCGTCTGTTGGAGACAGAGTCACCATCACTTGCCGGGCCAGTCAGAGTGTTAGTGACTGGTTGGCCTGGTATCAGCAGAAACCAGGGGAGCCCCCTAAGCTCCTCATCTCTAGGGCATCTACTTTAGAGATTGGGGTCGCATCAAGGTTCAGCGGCAGTGGATCTGGGACAGAATTCACTCTCACCATCAGCAGCCTGCAGCCTGATGATTATGCAACTTATTACTGCCAACAGTATAATACTTATTCGCTCACTTTCGGCGGAGGGACCAAGGTGGAGATCAAA

Figure 1

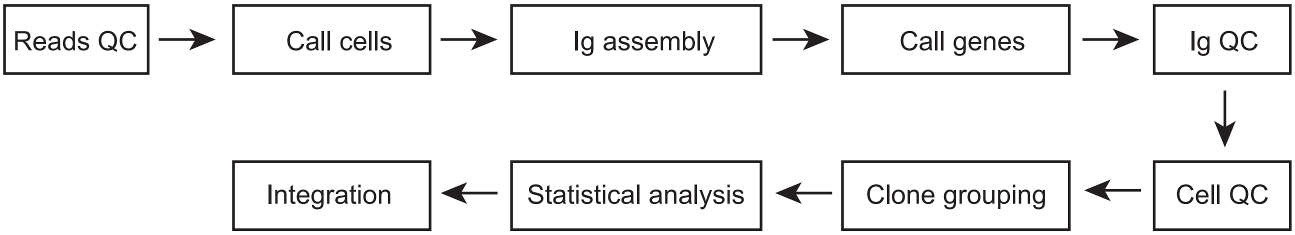

Reads QC
Call cells
Ig assembly
Call genes
Ig QC
Cell QC
Clone grouping
Statistical analysis
Integration

Figure 2

A

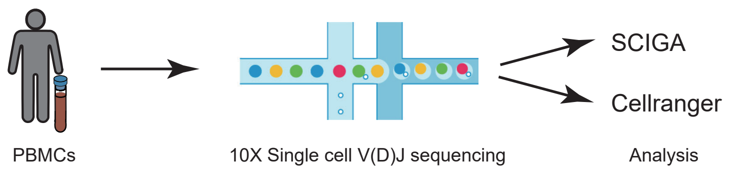

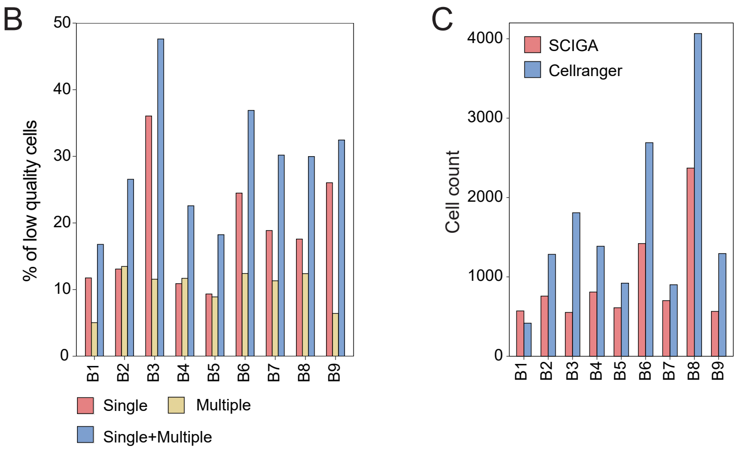

E

Output information	SCIGA	Cellranger
V(D)JC gene	√	√
Clone cluster	√	√
Contig sequence	√	√
Gene frequency	√	×
Clone frequency	√	×
Clonality	√	×
SHM rate	√	×
CDR3 length	√	×
Variable region sequence	√	×
Visualization	√	×
Shared immunoglobulin	√	×

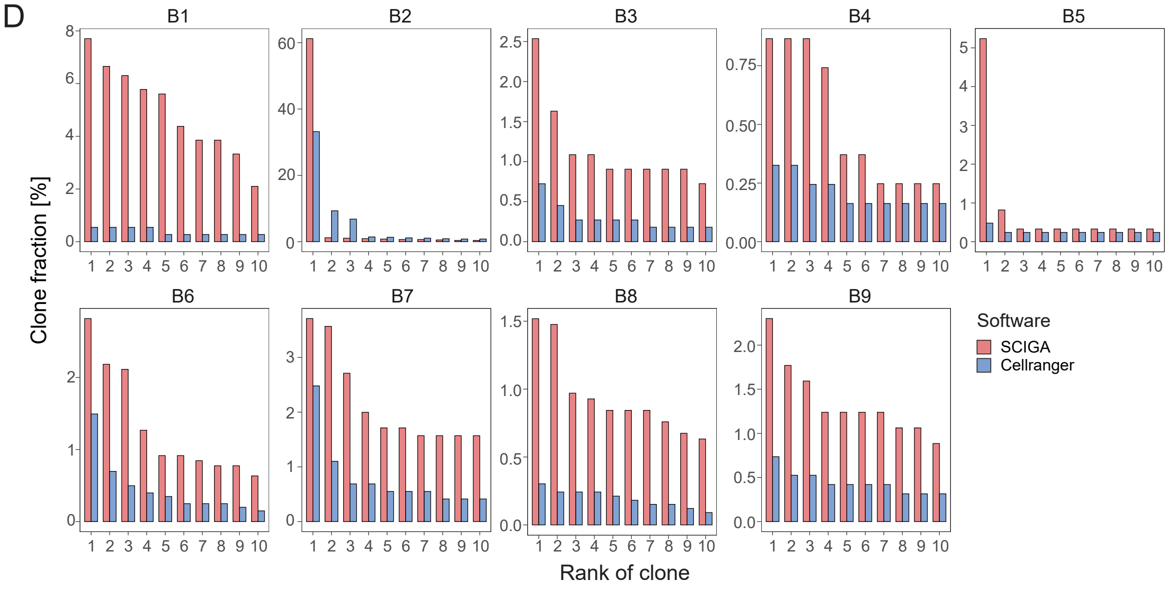

Figure 3

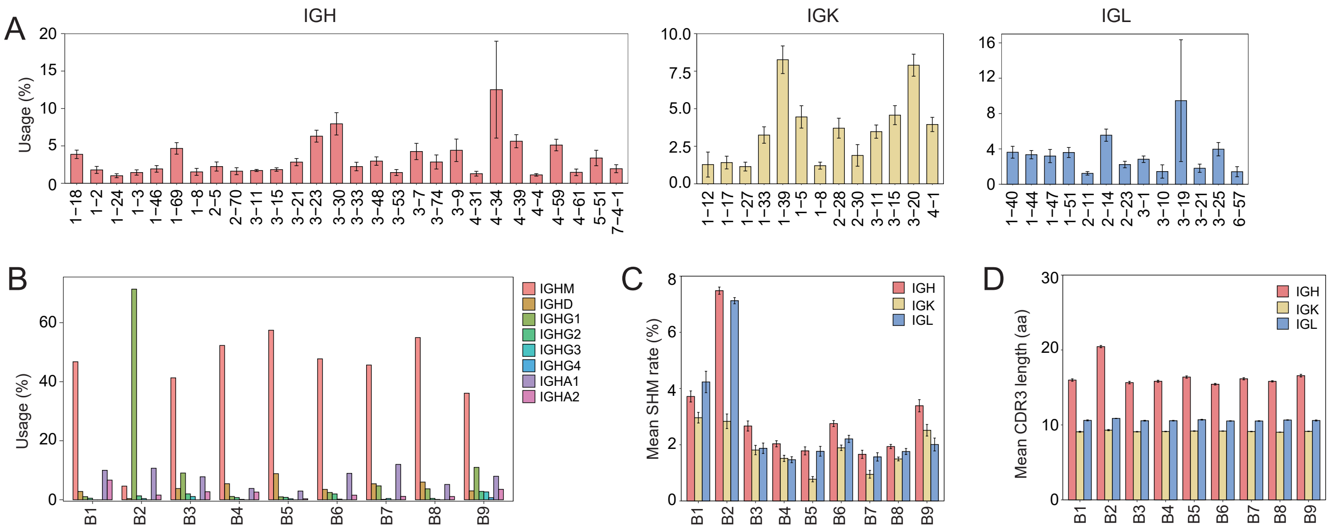
A
IGH
IGK
IGL
Usage (%)
1−18
1−2
1−24
1−3
1−46
1−69
1−8
2−5
2−70
3−11
3−15
3−21
3−23
3−30
3−33
3−48
3−53
3−7
3−74
3−9
4−31
4−34
4−39
4−4
4−59
4−61
5−51
7−4−1
1−12
1−17
1−27
1−33
1−39
1−5
1−8
2−28
2−30
3−11
3−15
3−20
4−1
1−40
1−44
1−47
1−51
2−11
2−14
2−23
3−1
3−10
3−19
3−21
3−25
6−57
B
Usage (%)
IGHM
IGHD
IGHG1
IGHG2
IGHG3
IGHG4
IGHA1
IGHA2
B1
B2
B3
B4
B5
B6
B7
B8
B9
C
Mean SHM rate (%)
IGH
IGK
IGL
D
Mean CDR3 length (aa)
IGH
IGK
IGL

Figure 4

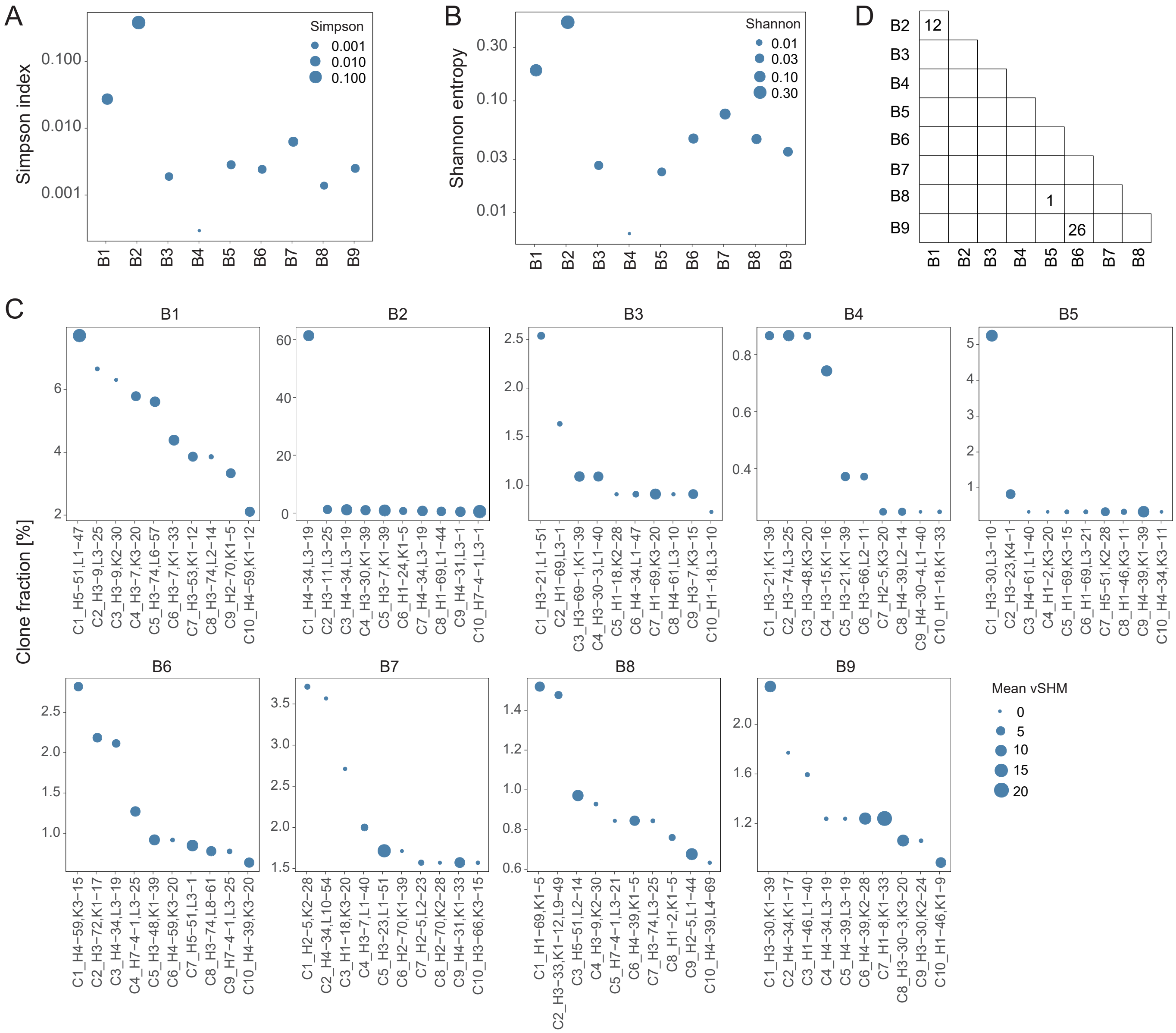

A
Simpson index
Simpson
0.001
0.010
0.100
B1 B2 B3 B4 B5 B6 B7 B8 B9
B
Shannon entropy
Shannon
0.01
0.03
0.10
0.30
D
12
1
26
C
Clone fraction [%]
B1
C1_H5−51,L1−47
C2_H3−9,L3−25
C3_H3−9,K2−30
C4_H3−7,K3−20
C5_H3−74,L6−57
C6_H3−7,K1−33
C7_H3−53,K1−12
C8_H3−74,L2−14
C9_H2−70,K1−5
C10_H4−59,K1−12
B2
C1_H4−34,L3−19
C2_H3−11,L3−25
C3_H4−34,L3−19
C4_H3−30,K1−39
C5_H3−7,K1−33
C6_H1−24,K1−5
C7_H4−34,L3−19
C8_H1−69,L1−44
C9_H4−31,L3−1
C10_H7−4−1,L3−1
B3
C1_H3−21,L1−51
C2_H1−69,L3−1
C3_H3−69−1,K1−39
C4_H3−30−3,L1−40
C5_H1−18,K2−28
C6_H4−34,L1−47
C7_H1−69,K3−20
C8_H4−61,L3−10
C9_H3−7,K3−15
C10_H1−18,L3−10
B4
C1_H3−21,K1−39
C2_H3−74,L3−25
C3_H3−48,K3−20
C4_H3−15,K1−16
C5_H3−21,K1−39
C6_H3−66,L2−11
C7_H2−5,K3−20
C8_H4−39,L2−14
C9_H4−30−4,L1−40
C10_H1−18,K1−33
B5
C1_H3−30,L3−10
C2_H3−23,K4−1
C3_H4−61,L1−40
C4_H1−2,K3−20
C5_H1−69,K3−15
C6_H1−69,L3−21
C7_H5−51,K2−28
C8_H1−46,K3−11
C9_H4−39,K1−39
C10_H4−34,K3−11
B6
C1_H4−59,K3−15
C2_H3−72,K1−17
C3_H4−34,L3−19
C4_H7−4−1,L3−25
C5_H3−48,K1−39
C6_H4−59,K3−20
C7_H5−51,L3−1
C8_H3−74,L8−61
C9_H7−4−1,L3−25
C10_H4−39,K3−20
B7
C1_H2−5,K2−28
C2_H4−34,L10−54
C3_H1−18,K3−20
C4_H3−7,L1−40
C5_H3−23,L1−51
C6_H2−70,K1−39
C7_H2−5,L2−23
C8_H2−70,K2−28
C9_H4−31,K1−33
C10_H3−66,K3−15
B8
C1_H1−69,K1−5
C2_H3−33,K1−12,L9−49
C3_H5−51,L2−14
C4_H3−9,K2−30
C5_H7−4−1,L3−21
C6_H4−39,K1−5
C7_H3−74,L3−25
C8_H1−2,K1−5
C9_H2−5,L1−44
C10_H4−39,L4−69
B9
C1_H3−30,K1−39
C2_H4−34,K1−17
C3_H1−46,L1−40
C4_H4−34,L3−19
C5_H4−39,L3−19
C6_H4−39,K2−28
C7_H1−8,K1−33
C8_H3−30−3,K3−20
C9_H3−30,K2−24
C10_H1−46,K1−9
Mean vSHM
0
5
10
15
20

Figure 5

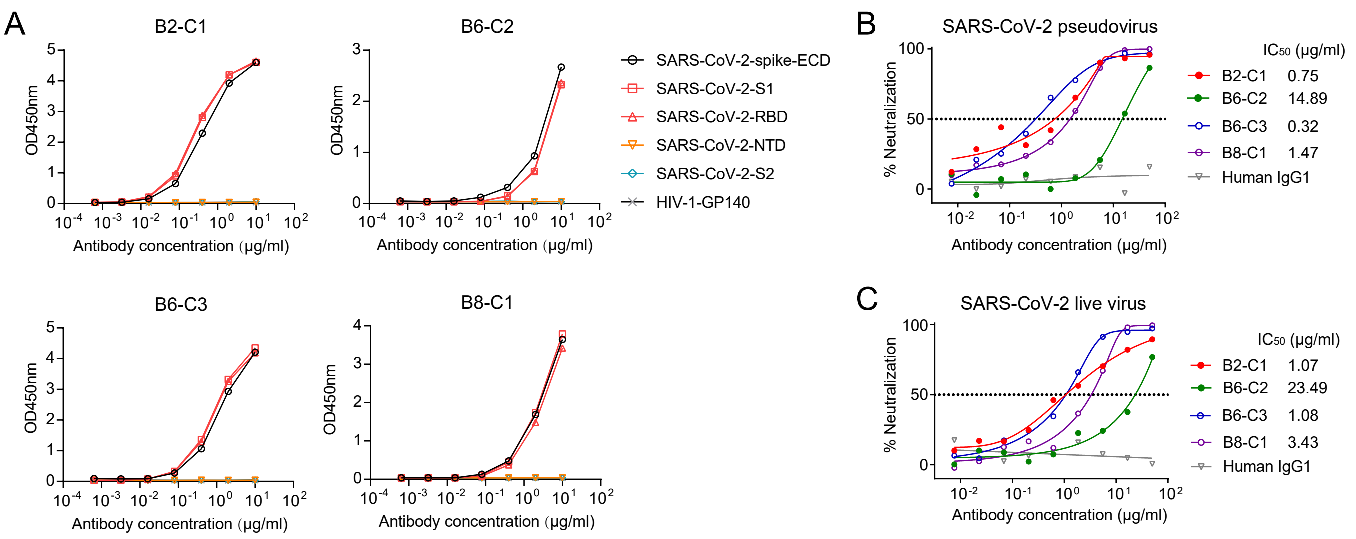
A
B2-C1
B6-C2
B6-C3
B8-C1
OD450nm
Antibody concentration (μg/ml)
SARS-CoV-2-spike-ECD
SARS-CoV-2-S1
SARS-CoV-2-RBD
SARS-CoV-2-NTD
SARS-CoV-2-S2
HIV-1-GP140
B
SARS-CoV-2 pseudovirus
% Neutralization
IC50 (μg/ml)
B2-C1 0.75
B6-C2 14.89
B6-C3 0.32
B8-C1 1.47
Human IgG1
C
SARS-CoV-2 live virus
IC50 (μg/ml)
B2-C1 1.07
B6-C2 23.49
B6-C3 1.08
B8-C1 3.43
Human IgG1

Figure S1

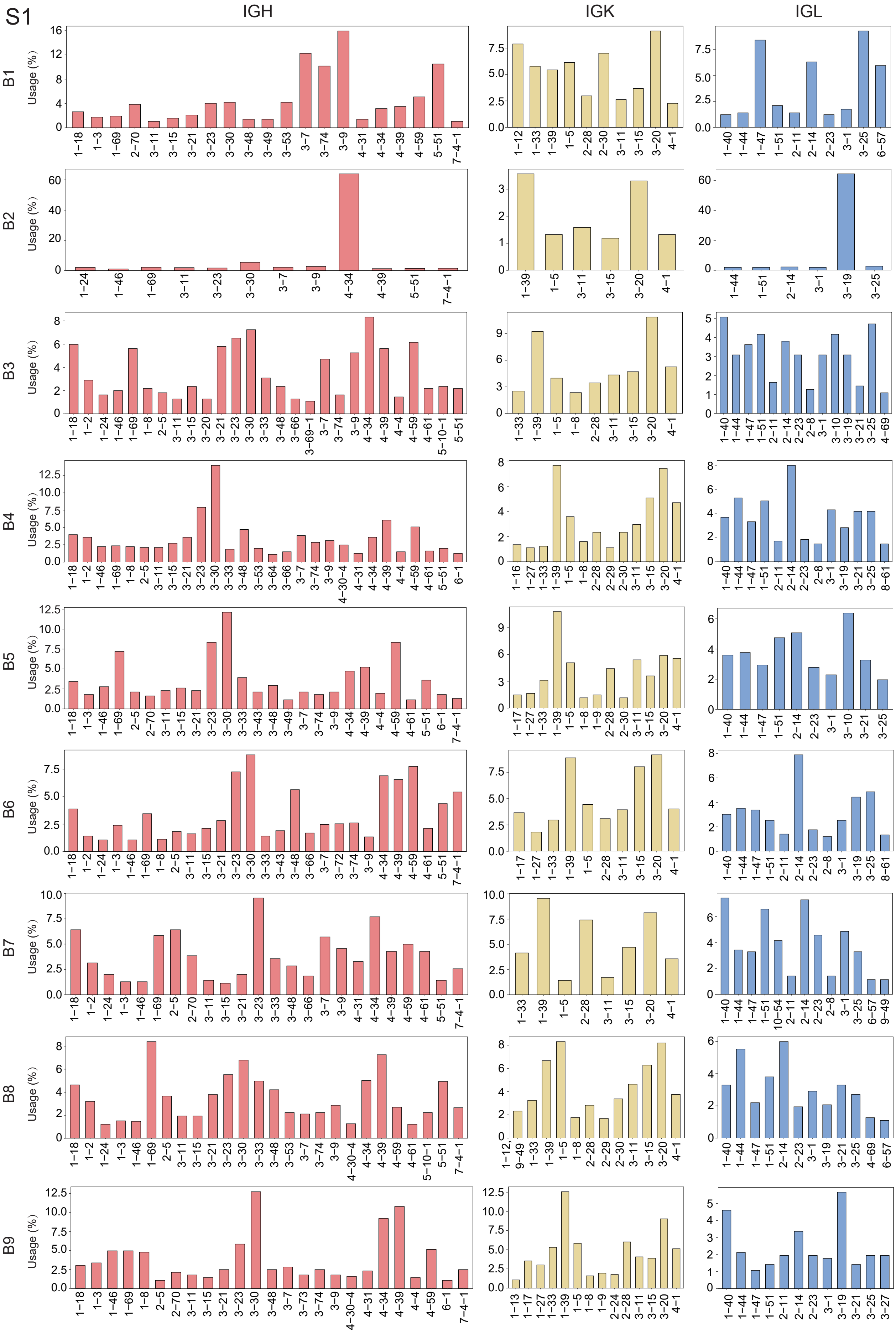

Figure S2

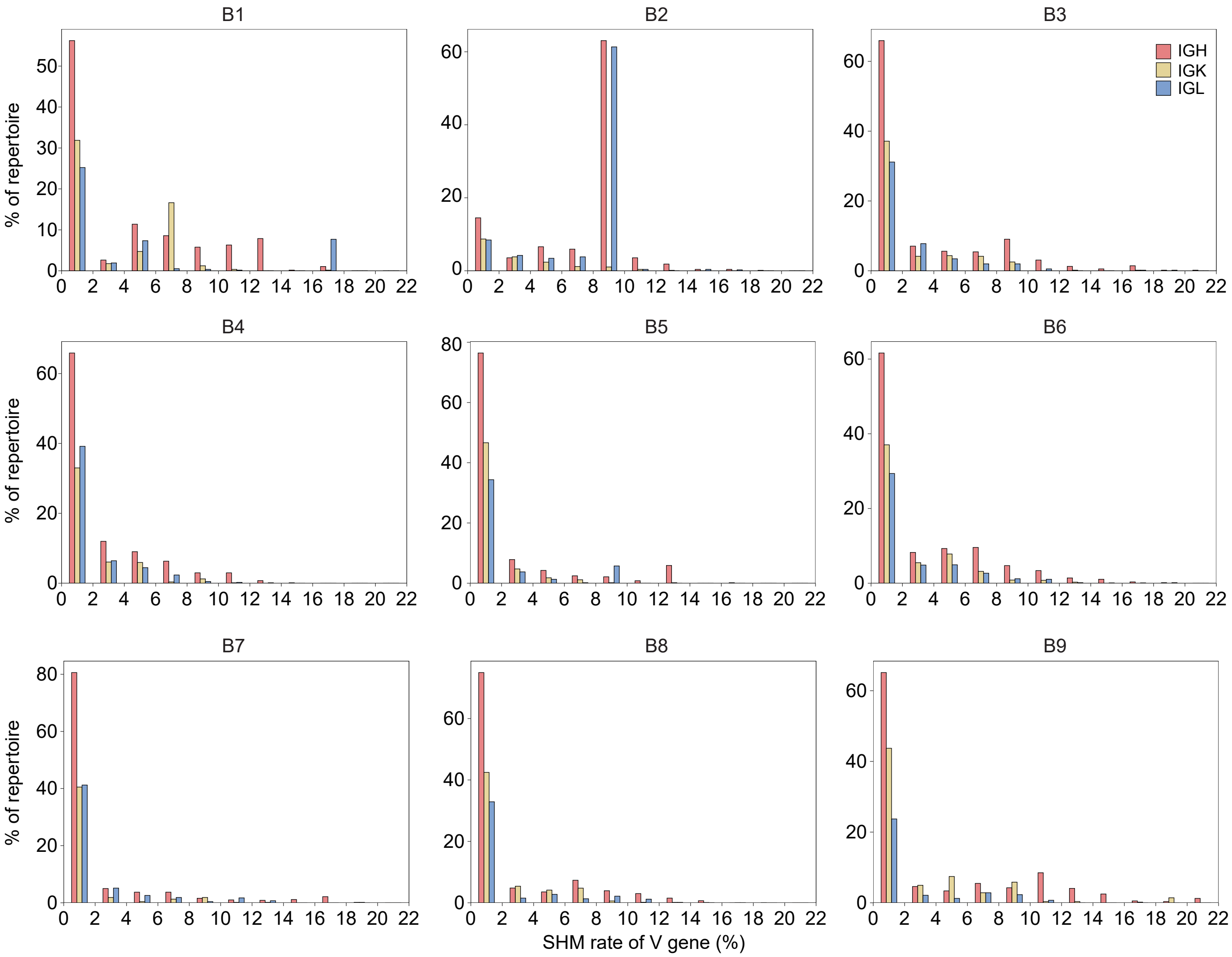
B1
B2
B3
B4
B5
B6
B7
B8
B9
IGH
IGK
IGL
% of repertoire
SHM rate of V gene (%)

Figure S3

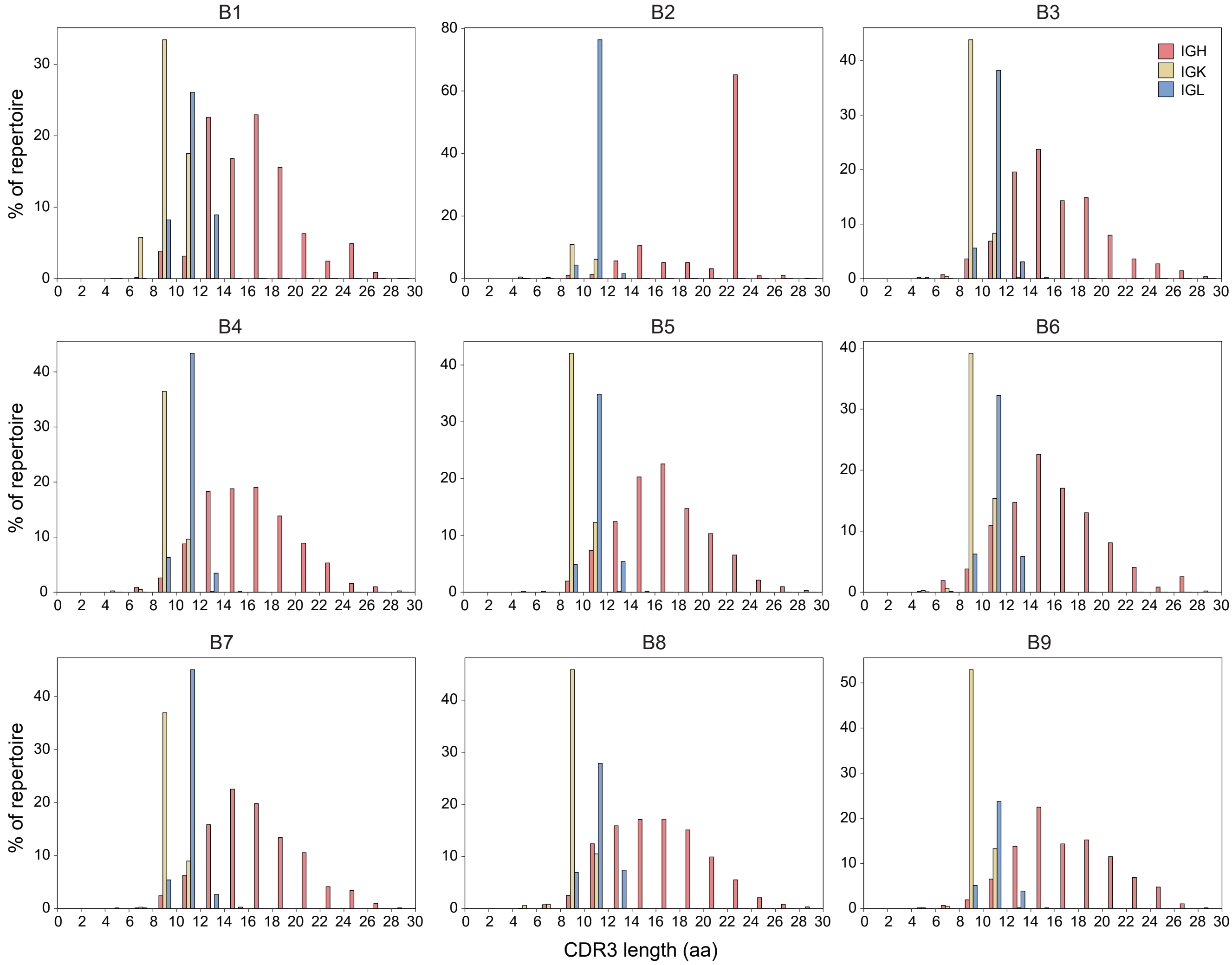

B1
B2
B3
B4
B5
B6
B7
B8
B9
IGH
IGK
IGL
% of repertoire
CDR3 length (aa)
0 2 4 6 8 10 12 14 16 18 20 22 24 26 28 30

Figure S4

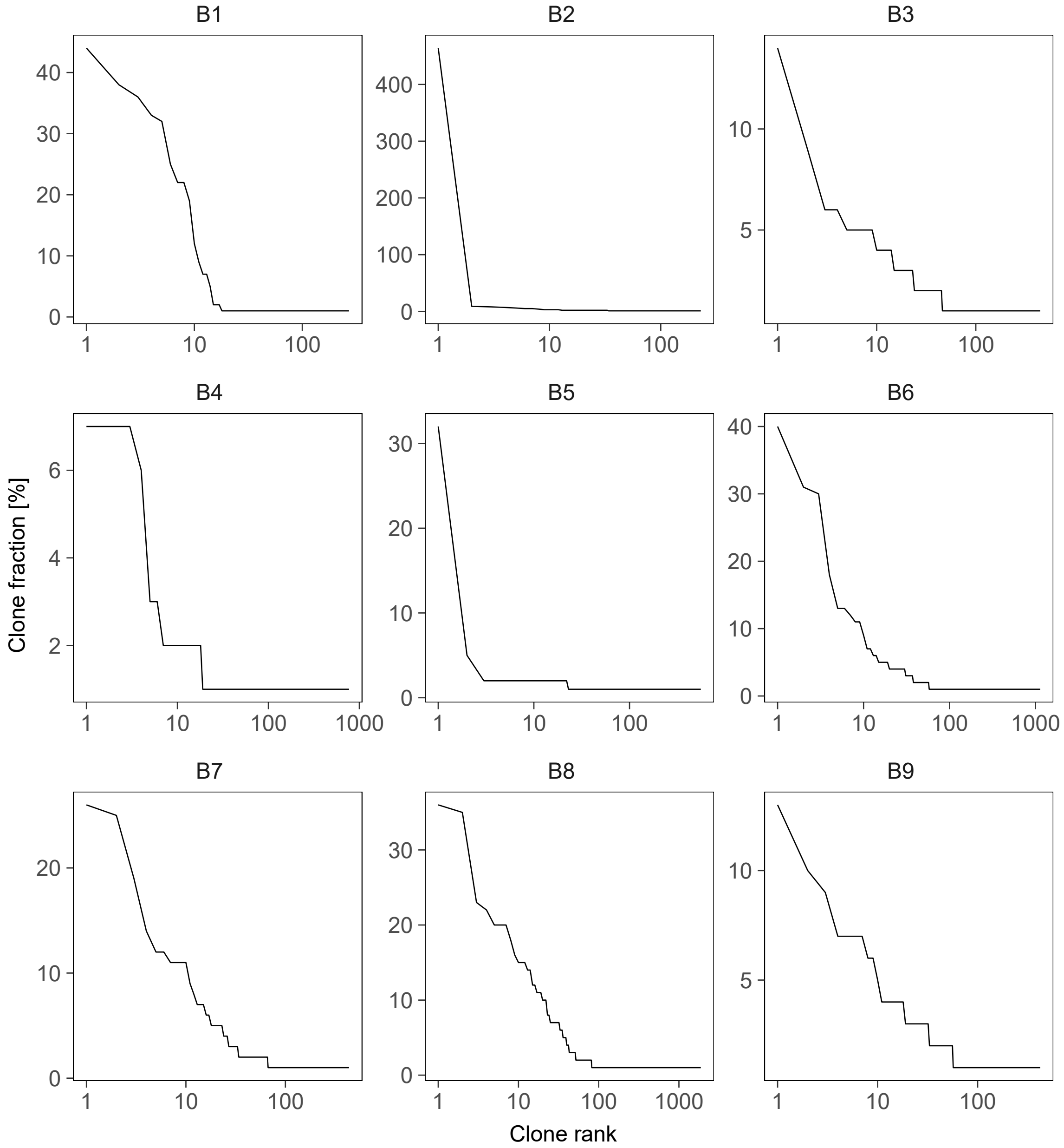

B1
B2
B3
B4
B5
B6
B7
B8
B9
Clone fraction [%]
Clone rank
0
10
20
30
40
100
200
300
400
5
2
4
6
1
1000

Figure S5

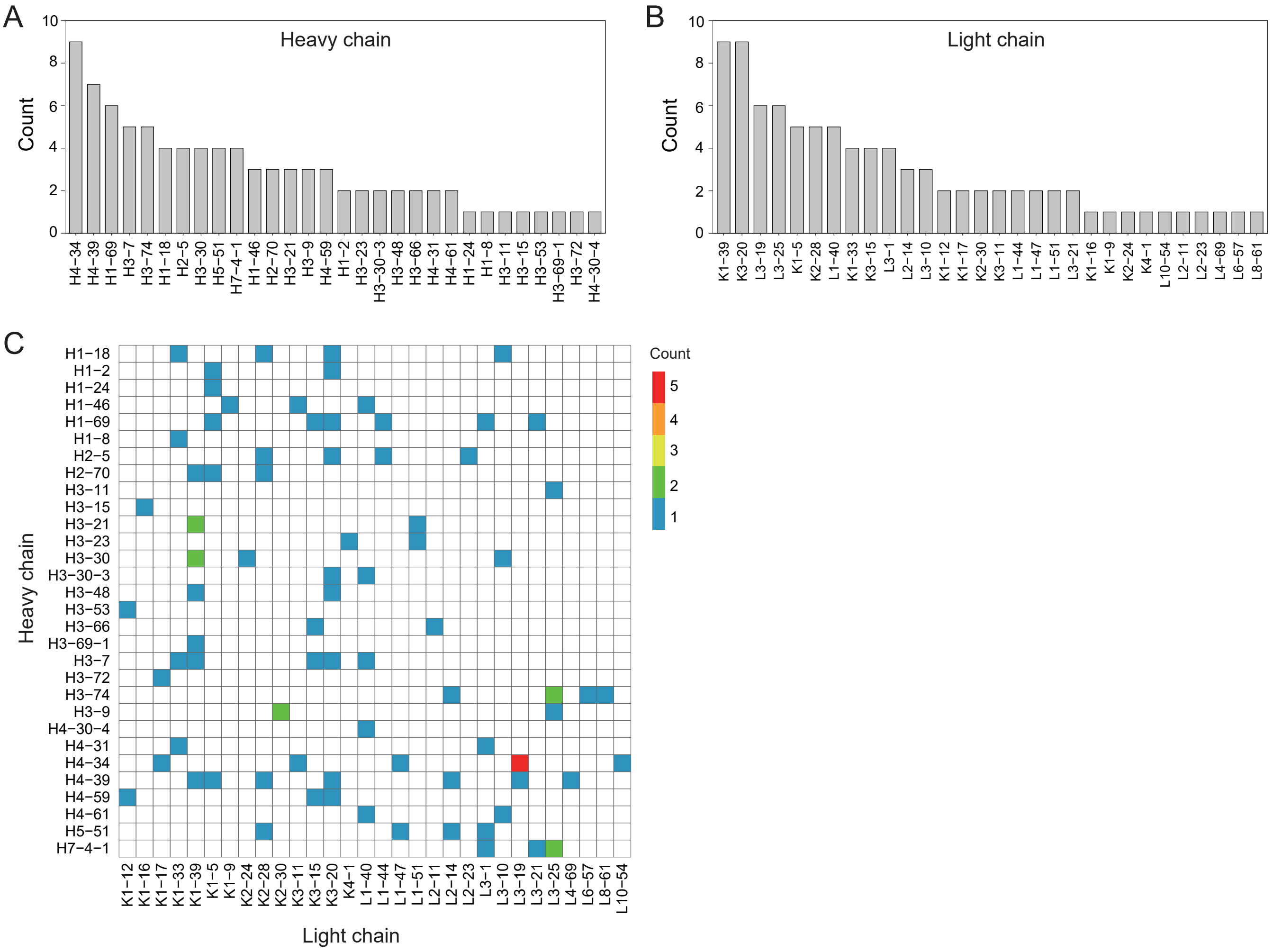
A
Heavy chain
Count
10
8
6
4
2
0
H4−34
H4−39
H1−69
H3−7
H3−74
H1−18
H2−5
H3−30
H5−51
H7−4−1
H1−46
H2−70
H3−21
H3−9
H4−59
H1−2
H3−23
H3−30−3
H3−48
H3−66
H4−31
H4−61
H1−24
H1−8
H3−11
H3−15
H3−53
H3−69−1
H3−72
H4−30−4
B
Light chain
Count
K1−39
K3−20
L3−19
L3−25
K1−5
K2−28
L1−40
K1−33
K3−15
L3−1
L2−14
L3−10
K1−12
K1−17
K2−30
K3−11
L1−44
L1−47
L1−51
L3−21
K1−16
K1−9
K2−24
K4−1
L10−54
L2−11
L2−23
L4−69
L6−57
L8−61
C
Heavy chain
Light chain
Count
5
4
3
2
1

Figure S6

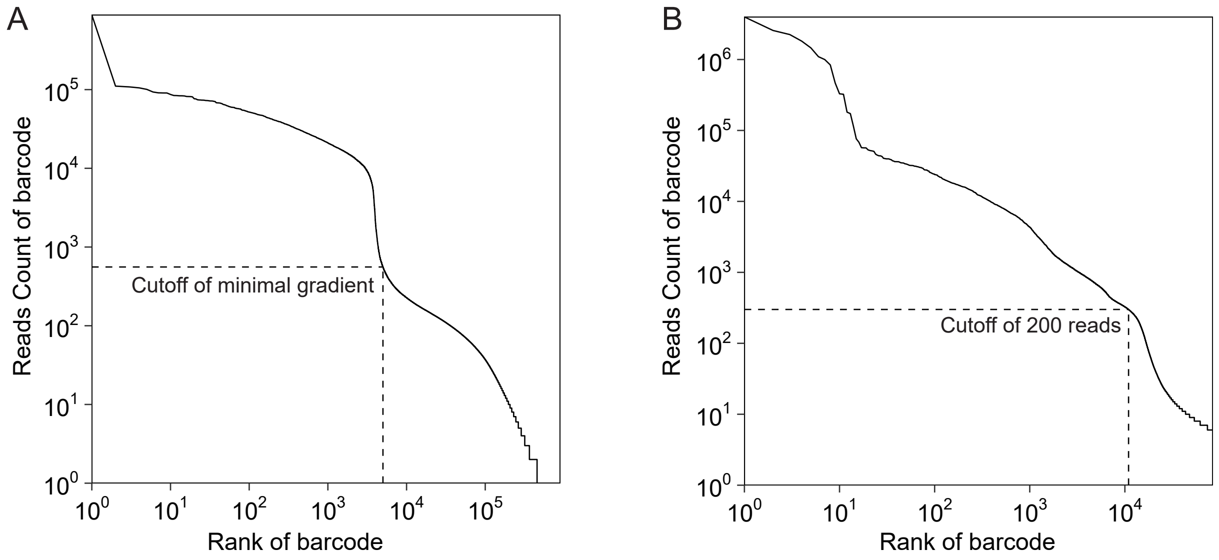
A
Reads Count of barcode
Rank of barcode
Cutoff of minimal gradient
10^0
10^1
10^2
10^3
10^4
10^5
B
Reads Count of barcode
Rank of barcode
Cutoff of 200 reads
10^6

Table S1

Patient ID	Gender	Age	Type
B1	Male	73	Severe
B2	Male	46	Severe
B3	Male	67	Severe
B4	Male	35	Mild
B5	Male	36	Mild
B6	Female	65	Severe
B7	Male	62	Severe
B8	Female	57	Severe
B9	Male	66	Severe

Table S2

CloneID	CloneSize	Heavy_Vgene	Heavy_Jgene	Light_Vgene	Light_Jgene	B1	B2	B5	B6	B8	B9
2	45	IGHV5-51	IGHJ4	IGLV1-47	IGLJ3	44	1	0	0	0	0
3	42	IGHV4-59	IGHJ4	IGKV3-15	IGKJ4	0	0	0	40	0	2
5	37	IGHV3-9	IGHJ4	IGKV2-30	IGKJ3	36	1	0	0	0	0
7	35	IGHV3-7	IGHJ4	IGKV3-20	IGKJ4	33	2	0	0	0	0
9	33	IGHV3-74	IGHJ4	IGLV6-57	IGLJ2,IGLJ3	32	1	0	0	0	0
10	33	IGHV4-34	IGHJ6	IGLV3-19	IGLJ3	0	0	0	30	0	3
11	33	IGHV3-30,IGHV3-30-5	IGHJ5	IGLV3-10	IGLJ2,IGLJ3	0	0	32	0	1	0
12	32	IGHV3-72	IGHJ4	IGKV1-17	IGKJ2	0	0	0	31	0	1
14	26	IGHV3-7	IGHJ4	IGKV1-33,IGKV1D-33	IGKJ5	25	1	0	0	0	0
16	23	IGHV3-74	IGHJ6	IGLV2-14	IGLJ2,IGLJ3	22	1	0	0	0	0
17	23	IGHV3-53	IGHJ5	IGKV1D-12	IGKJ4	22	1	0	0	0	0
23	20	IGHV2-70	IGHJ4	IGKV1-5	IGKJ2	19	1	0	0	0	0
24	19	IGHV7-4-1	IGHJ4	IGLV3-25	IGLJ2,IGLJ3	0	0	0	18	0	1
34	14	IGHV3-48	IGHJ4	IGKV1-39,IGKV1D-39	IGKJ2	0	0	0	13	0	1
36	14	IGHV4-59	IGHJ5	IGKV3-20	IGKJ1	0	0	0	13	0	1
38	13	IGHV4-59	IGHJ4	IGKV1-12,IGKV1D-12	IGKJ3	12	1	0	0	0	0
43	12	IGHV7-4-1	IGHJ4	IGLV3-25	IGLJ3	0	0	0	11	0	1
44	12	IGHV3-74	IGHJ6	IGLV8-61	IGLJ3	0	0	0	11	0	1
66	8	IGHV4-34	IGHJ4	IGLV2-11	IGLJ2,IGLJ3	7	1	0	0	0	0
68	8	IGHV1-2	IGHJ3	IGKV1-5	IGKJ1	0	0	0	7	0	1
96	6	IGHV3-49	IGHJ3	IGKV1-39,IGKV1D-39	IGKJ3	5	1	0	0	0	0
98	6	IGHV3-30-3	IGHJ4	IGKV1-5	IGKJ4	0	0	0	5	0	1
100	6	IGHV1-69	IGHJ6	IGKV1-27	IGKJ3	0	0	0	5	0	1
104	6	IGHV3-48	IGHJ1	IGKV1-27	IGKJ4	0	0	0	5	0	1
108	5	IGHV4-39	IGHJ4	IGKV1-39,IGKV1D-39	IGKJ4	0	0	0	4	0	1
110	5	IGHV1-8	IGHJ4	IGLV1-47	IGLJ3	0	0	0	3	0	2
121	5	IGHV3-74	IGHJ6	IGLV1-47	IGLJ3	0	0	0	4	0	1
122	5	IGHV3-43	IGHJ5	IGKV3-15	IGKJ4	0	0	0	4	0	1
124	5	IGHV3-43	IGHJ4	IGKV4-1	IGKJ4	0	0	0	4	0	1
126	5	IGHV3-43	IGHJ6	IGKV1-39,IGKV1D-39	IGKJ1	0	0	0	4	0	1
131	5	IGHV3-23,IGHV3-23D	IGHJ4	IGLV7-46	IGLJ3	0	0	0	4	0	1
135	4	IGHV3-13	IGHJ2	IGKV4-1	IGKJ5	0	0	0	3	0	1
153	4	IGHV4-59	IGHJ4	IGKV3-20	IGKJ1	0	0	0	3	0	1
157	4	IGHV1-3	IGHJ6	IGLV1-44	IGLJ6	0	0	0	3	0	1
176	3	IGHV3-30,IGHV3-30-3	IGHJ3	IGKV3-20	IGKJ1	0	0	0	2	0	1
207	3	IGHV4-4	IGHJ4	IGLV3-19	IGLJ2,IGLJ3	0	0	0	2	0	1
289	2	IGHV3-23,IGHV3-23D	IGHJ5	IGKV1-17	IGKJ4	0	0	0	1	0	1
340	2	IGHV3-11	IGHJ6	IGKV3-20	IGKJ1	0	0	0	1	0	1
369	2	IGHV3-30,IGHV3-33	IGHJ3	IGKV1-27	IGKJ1	1	1	0	0	0	0

Personal Cover Click here to access/download;Personal Cover;cover letter.pdf

SOUTHERN UNIVERSITY OF SCIENCE AND TECHNOLOGY

Zheng Zhang, MD & Ph.D.
Institute of Hepatology
Shenzhen 3rd People's Hospital

Nov 11, 2020
Editor,
GigaScience

Dear Editors:

I would like to submit the manuscript entitled "**SCIGA: A software for large-scale single-cell immunoglobulin repertories analysis**" to ***GigaScience*** to be considered for publication as a **technical note**. The relevant data have not been published elsewhere. No conflict of interest exits in the submission of this manuscript and manuscript is approved by all authors for publication.

10X single-cell V(D)J sequencing is a powerful tool for investigating the paired heavy- and light- chain repertories. Analyzing the 10X single-cell immunoglobulin repertories remains a challenge since the high diversity of immunoglobulin repertories and the few specialized software for analyzing those data. Here, we developed the SCIGA, a software for 10X single-cell immunoglobulin repertories analysis. It performs analysis by one line command. We also used SCIGA to analyze the single-cell immunoglobulin repertoires dataset from 9 of COVID-19 patients and identified 4 potent neutralizing antibodies against SARS-CoV-2.

SCIGA substantially allows researchers to quickly implement advanced analysis pipelines for large datasets generated by 10X single-cell V(D)J sequencing and obtain readable and graphic results. Our work on SCIGA provides more choices of software for researchers in the area of single-cell immunoglobulin repertoires, and will be of great interest to a large portion of the ***GigaScience*** readership.

Your suggestions and advice will be greatly appreciated.

Yours sincerely.

29 Bulan Road, Shenzhen, Guangdong Province 518100, China

Email: zhangzheng1975@aliyun.com

Zheng Zhang, MD & Ph.D.
Institute of Hepatology
Shenzhen 3rd People's Hospital

Sincerely,

Zheng Zhang, MD & Ph.D.

Institute for Hepatology, Shenzhen Third People's Hospital, The Second Affiliated Hospital, School of Medicine, Southern University of Science and Technology, Shenzhen, Guangdong 518112, China.

Phone: 86-755-81238983

Fax: 86-755-81238983

Email: zhangzheng1975@aliyun.com
