# Supplementary material for: SCIGA: Software for large-scale, single-cell immunoglobulin repertoire analysis
Source: Gigascience. 2021 Sep 28;10(9):giab050. doi: 10.1093/gigascience/giab050 (PMC8478610; doi:10.1093/gigascience/giab050)

GigaScience

SCIGA: A software for large-scale, single-cell immunoglobulin repertories analysis

--Manuscript Draft--

**Manuscript Number:**	GIGA-D-20-00341R1
**Full Title:**	SCIGA: A software for large-scale, single-cell immunoglobulin repertories analysis
**Article Type:**	Technical Note
**Funding Information:**	Foundation for Distinguished Young Talents in Higher Education of Guangdong (82025022) — Dr. Zheng Zhang
**Abstract:**	B cell immunoglobulin repertoires with paired heavy and light chain can be determined by the 10X single-cell V(D)J sequencing . Precise and quick analysis of 10X single-cell immunoglobulin repertoires remains a challenge due to the high diversity of immunoglobulin repertoires and a lack of specialized software that can analyze such diverse data. In this study, specialized software for 10X single-cell immunoglobulin repertoire analysis was developed. SCIGA (Single-Cell Immunoglobulin Repertoire Analysis) is an easy-to-use pipeline that performs read trimming, immunoglobulin sequence assembly and annotation, heavy and light chain pairing, statistical analysis, visualization, and multiple sample integration analysis, which is all achieved by using a one-line command. Then SCIGA was used to profile the single-cell immunoglobulin repertoires of nine coronavirus disease 2019 (COVID-19) patients. Four neutralizing antibodies against severe acute respiratory syndrome coronavirus 2 (SARS-CoV-2) were identified from these repertoires.
**Corresponding Author:**	Zheng Zhang<br>Southern University of Science and Technology<br>Shenzhen, CHINA
**Corresponding Author Secondary Information:**	
**Corresponding Author's Institution:**	Southern University of Science and Technology
**Corresponding Author's Secondary Institution:**	
**First Author:**	Zheng Zhang
**First Author Secondary Information:**	
**Order of Authors:**	Zheng Zhang
	Haocheng Ye
	Lin Cheng
	Bin Ju
	Gang Xu
	Yang Liu
	Lifei Wang
**Order of Authors Secondary Information:**	
**Response to Reviewers:**	The language of this manuscript has been revised by a language company. The software has received the RRID and Biotools ID (Line 244). We have fully addressed the points, as follows:<br><br>Reviewer #1: Ye and colleagues develop and describe a software for large scale, single-cell immunoglobulin repertoires analysis.<br><br>1) Are the methods appropriate to the aims of the study, are they well described, and are necessary controls included?

No. The sample processing, post-sequencing analysis and statistics is not fully discribed.
Response:
Thank you for the comment. The methods that describe the sample processing in Line 533-547, the post-sequencing analysis in Line 549-560, the statistics in Line 516-522.

2) Are the conclusions adequately supported by the data shown?
Partially, but there is not enough information in the manuscript, such as how the antibodies were selected.
Response:
Thank you for the comment. The method to select antibodies as follows: 1) Screen the clone with fraction ≥ 1% and cell number ≥ 20; 2) For each clone, screen the IgG immunoglobulin; 3) Trim the nucleotide sequences of immunoglobulin and retain the variable region; 4) Calculate the abundance of trimmed immunoglobulin sequences. The immunoglobulin sequence with the highest abundance in a clone is the monoclonal antibody candidate. (Line 564-569)

3) Please indicate the quality of language in the manuscript. Does it require a heavy editing for language and clarity?
Reasonable. Only a few corrections (below)
Response:
Thank you for the comment. The language of this manuscript has been revised by a language company.

4) Are you able to assess all statistics in the manuscript, including the appropriateness of statistical tests used?
No.
Response:
Thank you for the comment. The statistics in Line 516-522. But we didn't use statistical test in this study.

Major comments
* Productive BCRs are important in many situations, however, understanding the non-productive repertoire can be just as important in the context of haematological malignancies. Is this a possibility in your workflow? If not, this should be included.
Response:
Thank you for the comment. We modified the SCIGA program and retained the non-productive BCRs in the final result. A tag called "Productive" was added to denote whether the BCR is productive.

* Can the user introduce a custom reference for gene calling?
Response:
Thank you for the comment. Yes. Users can introduce a custom reference using the parameter "-index". Lesson to help users building the custom reference index in https://github.com/sciensic/SCIGA.

* Line 95: "The chains with a certainty score less than a specified threshold are discarded" can the authors explain how the threshold is calculated?
Response:
Thank you for the comment. The certainty score is defined as the number of UMIs supporting the chain divided by the total number of UMIs of all heavy or light chains (Line 493-494). We set the default threshold as 80, followed the reference 7. Users can set a custom threshold using the parameter "-certainty".

* Line 97: "then the cells without pairing heavy- and light- chains are filtered out" Given the cost of sequencing by 10X, it is often helpful to include the analysis of cells with the capture of only one chain (such as clonality scores for rare cell subsets). Is there a possibility to include these?
Response:
Thank you for the comment.
Firstly, one of the important functions of 10X V(D)J sequencing is that 10X V(D)J sequencing can produce paired heavy and light repertoires. That why we prefer to just retain the cells with the capture of paired chains in the final results. However, SCIGA retains the intermediate results, including the cells with the capture of only one chain

(in the file called “3_filtered_contig_annotation”). Users can use the intermediate results if they need to analyze the cells with only one chain.
Secondly, SCIGA groups clone using both heavy and light chains. It is hard to group a cell with only one chain into certain clonal lineage. For example, A cell has paired heavy and light chain, while B cell has only one identical heavy chain. We are not sure whether the A and B cell belong to a clonal lineage.

* Line 100: what is the threshold used for defining clones?
Response:
Thank you for the comment. We set the default threshold as 90 (Line 501), followed the reference 2. Users can set a custom threshold using the parameter “- identity”.

* In figure 2C, sample B1 has moer cells passing filtering than CellRanger. Can the authors discuss why this sample is different from the rest?
Response:
Thank you for the comment.
The cell number mainly relates to the two aspects: 1) Immunoglobulin sequence assembly. SCIGA performs assembly using SSAKE. Only the barcodes with read number over a given threshold (default as 200) are performed assembly. This will speed up the software running, but fail to capture the cells with low V(D)J transcripts abundance. For the Cell Ranger, it performs assembly on all barcodes based on the De Bruijn graph. Cell Ranger considers the barcode as cell when the barcode includes a productive, confident contig. The two assembly methods make a difference on cell number. Generally, the cell number of SCIGA is less than Cell Ranger when the sequencing depth is low. 2) Cell quality controlling. For Cell Ranger, the output still included considerably low-quality cells, which either had multiple heavy or light chains, or had only one chain. SCIGA implements the cell quality control process and only output the high-quality cells.
In our dataset, the sample B1 had the highest sequencing depth (Figure R1 of the “Figures for responding to reviewers”). It meant that, more barcodes of sample B1 over the threshold in the read number, more high-quality contigs were assembled, and more cells pass the cell quality control. This may be the reason why sample B1 has more cells passing filtering than Cell Ranger.

* Most users will be comparing the clonality between multiple samples captured at differing cell depths. What have the authors done to address the issue of cell depth in the statistical analyses (such as subsampling etc), given that cell depth have a major impact on Simpson and Shannon indices? Was this point addressed in Figure 4?
Response:
Thank you for the comment. It is a controversial point. Some researchers suggested that subsampling analysis resolves randomly generated differences in sequence depth, but also results in discarding data, which leads to loss of assay power. (reference 20). Therefore, on one hand, SCIGA program computes the Simpson and Shannon indices using all cells of a sample. On the other hand, SCIGA program produces the rarefaction curves to show the value of two indices under different cell depth. (Figure R2 of the “Figures for responding to reviewers”). The rarefaction curves will be benefit to understand how the cell depth affect the two indices.

* Can the authors describe what the differences are between the Simpson and Shannon indices to guide the reader for their anlayses? A discussion of the pros and cons of each would be useful.
Response:
Thank you for the comment. A literature has discussed the difference between the two indices. (Bo-Ra Kim, et al. Deciphering Diversity Indices for a Better Understanding of Microbial Communities. Journal of Microbiology and Biotechnology). Both the two indices have specific biases. The Shannon index places a greater weight on clone richness. The value increases when the clone number increases. Whereas, the Simpson index considers clone evenness more than clone richness in its measurement. It tends to indicate the clone dominance.

* Could the authors provide a plot or table of the clone sizes of the 4 expressed antibodies compared the rest of the BCR repertoire for each individual? How did the authors choose these antibodies? Were there only 4 clones that >1%/>20 cells, or were these selected. If the latter, please can the authors show how representative

these expressed clones are of the total expanded IgG+ B cell population?
Response:
Thank you for the comment.
The plots of the clone sizes of the 4 expressed antibodies compared the rest of the BCR repertoire for each individual are in the Figure 4C and Figure S4. * denotes the antibody.
The method to select antibodies as follows: 1) Screen the clone with fraction ≥ 1% and cell number ≥ 20; 2) For each clone, screen the IgG immunoglobulin; 3) Trim the nucleotide sequences of immunoglobulin and retain the variable region; 4) Calculate the abundance of trimmed immunoglobulin sequences. The immunoglobulin sequence with the highest abundance in a clone is the monoclonal antibody candidate. (Line 564-569)
There were only 4 IgG clones that > 1% and > 20 cells.

* In line 205, they authors suggest that IGHV4-34 is associated with SARS-COV2. This cannot be deduced from the data provided given that there are no healthy controls for comparison. (It should be noted that this association has been seen in some other papers previously. However, I belive that IGHV3-33 is also associated which is not picked up here. It would be worth discussing this in the context of the current literature of BCR repertoire from COVID patients).
Response:
Thank you for the comment. The statement may be not serious, we removed it.

* Could the authors provide the SHM and IGHG information about the 4 expressed antibodies given that this is in the discussion (lines 211-213)?
Response:
Thank you for the comment. We added a table to show the SHM and IGHG information about the 4 expressed antibodies (Table S3). For the discussion, it was just about the strategy for screening antibody candidate. The reason why we didn’t exclude the antibody with SHM < 2% was that several potent neutralizing antibodies against SARS-CoV-2 have low SHM rates. However, we didn’t pick out the antibody with SHM < 2% using our strategy. Our statement may lead to misunderstanding, we re-phrased it (Line 218-223).

Minor comments
* The language used is informal at ponits, such as line 149 "who preferred to use IGHG1", instead of "with elevated IGHG1 usage". This needs to be tightened up as the preference of the patient (mental) is unlikely related to their IGH usage! Also line 146 "pretty high usage in patient B2". Also, line 164.
Response:
Thank you for the comment. This has been corrected. (Line 153 and 155-156)

* SHM rate is not measured (line 151), rather the level of SHM. This needs to be corrected. Also line 196.
Response:
Thank you for the comment. This has been corrected. (Line 157-158 and 206)

* Line 213: the word "Totally" does not make sense here.
Response:
Thank you for the comment. This has been corrected. (Line 223)

Reviewer #2: The manuscript entitled: "SCIGA: A software for large-scale, single-cell immunoglobulin repertories analysis" describes a computational platform for the single B cell analysis of data derived from 10xgenomics experiments. The authors state that SCIGA is superior to the challenger software especially by providing additional data regarding the characteristics of the identified antibodies and improves the sequence calls. The authors describe how SCIGA enables the isolation of 4 SARS-CoV-2 specific mAbs that exhibit neutralisation capacity.
Indeed, the rise in the usage of single cell platforms and particularly, 10xgenomics calls for the development of computational platforms to facilitate the data interpolation. This need was addressed in the submitted manuscript however, I would not recommend publishing the paper in its current version.
Beyond the comments specific below: the manuscript in not coherent and I had great

difficulties reading it and at some paragraphs I completely was confused by some of the statements. The method section is lacking several key components (cloning and expression mAbs) and in general the manuscript requires a professional native English speaker, scientific editing.

Comments:
Line 79: "Since the 10x system generate a large of Gel…" - sentence not clear. All of the paragraph describing the cell call is not clear - please re-phrase paragraph.
Response:
Thank you for the comment. We have re-phrased this paragraph. (Line 79-82)

Line 94: State specifically the criteria for clone clustering.
Response:
Thank you for the comment. Clonal lineage is defined as the cells that have identical VH, JH, VL and JL genes, identical H-CDR3 length, and over a given similarity threshold (default value is 90) of H-CDR3 nucleotide sequences. The detail for clone clustering in Line 499-513.

Line 99: What is the similarity threshold ? 100%, 80% identity - on the basis of nt or AA ?
Response:
Thank you for the comment. We set the default threshold as 90 on the basis of nt, followed the reference 2. Users can set a custom threshold by using the parameter "-identity".

What is the meaning of V gene usage when analyzing B cell from PBMCs. There was no enrichment to B cell sub-types, so it would be good if the authors will clarify the biological meaning of the V gene usage for such a broad population of B cells. Furthermore, it is not clear why they used only 1% of the cells in this analysis.
Response:
Thank you for the comment.
The previous study said that the "unbiased" B-cell repertoire analysis will provide a more accurate view of the overall B-cell repertoire. (Linling He, et al. Toward a more accurate view of human B-cell repertoire by next-generation sequencing, unbiased repertoire capture and single-molecule barcoding. Scientific Report). Therefore, analyzing the V gene usage of a broad population of B cells can help knowing how the infection shapes the overall B-cell repertoire.
Under infection, the antigens will drive the specific B cells to proliferate, with the expansion of specific V gene. The elevated usage of V gene may be associated with the infection. Therefore, we only investigated the V gene with usage > 1%, followed the reference 2.

Line 149 - "who preferred to use…" - the patient did no prefer to use rather the response was characterised by …
Response:
Thank you for the comment. We have re-phrased this sentence. (Line 155-156)

In general for the section: " Profiling immunoglobulin repertoires of COVID-19 by SCIGA" - the fact that the authors did not use any sorting prior the single cell sequencing impacts the ability to generate meaningful insights regarding the repertoire of the antibodies. I am not sure that the data supports repertoire analysis. Maybe should not use the term repertoire.
Response:
Thank you for the comment. We think that plasma cells are important component of immunoglobulin repertoires. However, there is no marker that can sort the plasma cells out from PBMCs. That why we did not use any sorting prior the single cell sequencing.

Line 158-159: This is a circular logic - if both patients show high clonality - meaning that they have large clones. This sentence should be re-phrased.
Response:
Thank you for the comment. We have re-phrased this sentence. (Line 164-165)

Line 161: at line 158-159, the author state that patient B1 had large clones but here they state that patient B1 has even clone frequency.

	Response:<br>Thank you for the comment. The statement was not correct and we have removed it.<br><br>Line 164: the clone do not prefer to use rather - there was a preferential usage of XYZ V family in the clones…<br>Response:<br>Thank you for the comment. We have re-phrased this paragraph. (Line 171-174)<br><br>Line 169-170: This paragraph is not clear at all. Please re-phrase.<br>Response:<br>Thank you for the comment. We have re-phrased this paragraph. (Line 176-178)<br><br>Line 154: Clonal lineage grouping - it is not clear from the description what was the clonal lineage grouping serve ? insights regarding the repertoire ? a methodological strategy to identify monoclonal antibodies? Moreover, looking for shared clones in population of PBMC is not likely to yield a result - can the authors clarify how they found shared clones?<br>Response:<br>Thank you for the comment.<br>When encountering an antigen, the specific B cell will be activated and proliferate, forming the B cell clonal lineage. The expanded clonal lineage may be associated with the antigen. Grouping the clonal lineage can help find out the clones that response to the antigen.<br>The method to select antibodies as follows: 1) Screen the clone with fraction ≥ 1% and cell number ≥ 20; 2) For each clone, screen the IgG immunoglobulin; 3) Trim the nucleotide sequences of immunoglobulin and retain the variable region; 4) Calculate the abundance of trimmed immunoglobulin sequences. The immunoglobulin sequence with the highest abundance in a clone is the monoclonal antibody candidate. (Line 564-569)<br>Shared immunoglobulins are defined as the immunoglobulins from different samples that can be clustered into the same clonal lineage. (Line 529-531)<br><br>Line 175: it seems that the only criteria for identifying nt mAbs in the samples was frequency of the clones and cells. What about SHM? Was this taken inot account? What about isotype?<br>Response:<br>Thank you for the comment. We didn't take into account the SHM, since several potent neutralizing antibodies against SARS-CoV-2 have low SHM rates (reference 16-18). The isotype of the selected antibodies must be IGHG.<br><br>Line 195: It is hard to conclude any insights regarding the repertoire of B cell (or BCRs) based on the analysis of single cells from PBMC. I recommend to re-phrase the statement to emphasis that the data can inform us on the V gene usage/SHM etc. on the identified cells by single cell analysis.<br>Response:<br>Thank you for the comment. Maybe you suggested the second paragraph of results (Title: Profiling immunoglobulin repertoires of COVID-19 by SCIGA). We have re-phrased the statement. The title was changed to "Determining the features of immunoglobulin repertoires of COVID-19 using SCIGA".<br><br>Figure S4 - why to plot the CDR3 length of the heavy and light chain on the same graph. This is confusing.<br>Response:<br>Thank you for the comment. Maybe you suggested the Figure S3. We have modified the plot.<br><br>Methods: the authors did not describe how they - 1) sequenced the amplicons including the sequencer platform, what reagent kit was used for the sequencing, 2) the authors did not describe how they cloned and expressed and purified the monoclonal antibodies?<br>Response:<br>Thank you for the comment. 1) The method for sample processing and sequencing in Line 534-547. 2) The method for expressing and purifying the monoclonal antibodies in Line 570-578.

	General comment: I would recommend the authors to use the services of scientific editor (native speaker) as many sentences require grammar revisions.<br>Response:<br>Thank you for the comment. The manuscript has been revised by a language company.
Additional Information:	
Question	Response
Are you submitting this manuscript to a special series or article collection?	No
**Experimental design and statistics**<br><br>Full details of the experimental design and statistical methods used should be given in the Methods section, as detailed in our Minimum Standards Reporting Checklist. Information essential to interpreting the data presented should be made available in the figure legends.<br><br>Have you included all the information requested in your manuscript?	Yes
**Resources**<br><br>A description of all resources used, including antibodies, cell lines, animals and software tools, with enough information to allow them to be uniquely identified, should be included in the Methods section. Authors are strongly encouraged to cite Research Resource Identifiers (RRIDs) for antibodies, model organisms and tools, where possible.<br><br>Have you included the information requested as detailed in our Minimum Standards Reporting Checklist?	Yes
**Availability of data and materials**<br><br>All datasets and code on which the conclusions of the paper rely must be either included in your submission or deposited in publicly available repositories (where available and ethically	Yes

appropriate), referencing such data using a unique identifier in the references and in the "Availability of Data and Materials" section of your manuscript.<br><br>Have you have met the above requirement as detailed in our Minimum Standards Reporting Checklist?	

Powered by Editorial Manager® and ProduXion Manager® from Aries Systems Corporation

Manuscript Click here to access/download;Manuscript;Manuscript.pdf

SCIGA: A software for large-scale, single-cell immunoglobulin repertoires analysis

Haocheng Ye[1,3#], Lin Cheng[1#], Bin Ju[1], Gang Xu[1], Yang Liu[1], Lifei Wang[2*], Zheng Zhang[1*]

[1]Institute for Hepatology, National Clinical Research Center for Infectious Disease, Shenzhen Third People's Hospital, The Second Affiliated Hospital, School of Medicine, Southern University of Science and Technology, Shenzhen, Guangdong 518112, China.

[2]Department of Radiology, National Clinical Research Center for Infectious Disease, Shenzhen Third People's Hospital, The Second Affiliated Hospital, School of Medicine, Southern University of Science and Technology, Shenzhen, Guangdong 518112, China.

[3]CAS Key Laboratory of Pathogenic Microbiology and Immunology, Institute of Microbiology, Chinese Academy of Sciences (CAS), Beijing, 100101, China

#These authors contributed equally.

***Correspondence:**

Zheng Zhang. Institute of Hepatology, Shenzhen 3rd People's Hospital, Shenzhen, Guangdong Province 518100, China; Email: zhangzheng1975@aliyun.com.

Lifei Wang. Department of Radiology, Shenzhen 3rd People's Hospital, Shenzhen, Guangdong Province 518100, China; Email: wanglf007n@163.com.

Abstract

B cell immunoglobulin repertoires with paired heavy and light chain can be determined by the 10X single-cell V(D)J sequencing. Precise and quick analysis of 10X single-cell immunoglobulin repertoires remains a challenge due to the high diversity of immunoglobulin repertoires and a lack of specialized software that can analyze such diverse data. In this study, specialized software for 10X single-cell immunoglobulin repertoire analysis was developed. SCIGA (Single-Cell Immunoglobulin Repertoire Analysis) is an easy-to-use pipeline that performs read trimming, immunoglobulin sequence assembly and annotation, heavy and light chain pairing, statistical analysis, visualization, and multiple sample integration analysis, which is all achieved by using a one-line command. Then SCIGA was used to profile the single-cell immunoglobulin repertoires of nine coronavirus disease 2019 (COVID-19) patients. Four neutralizing antibodies against severe acute respiratory syndrome coronavirus 2 (SARS-CoV-2) were identified from these repertoires.

Key words: Software; Single-cell; Immunoglobulin repertoires; COVID-19; Antibody

Introduction

The diversity of B cell immunoglobulin is an important characteristic of the adaptive immune system. It is developed through the rearrangement of variable

V, (diversity D) and the joining of J gene segments, which is referred to as V(D)J, the pairing of heavy and light chains, and the somatic hypermutation (SHM) [1]. Exposure to infections and environmental factors shapes the repertoire of B cell immunoglobulins [2-4], and leads to clonal expansion of immune cells, allowing them to change into different types of cells to respond to a specific antigen. Understanding these immunoglobulin repertoires can help researchers to discover antibodies, monitor vaccination responses and infer B cell trafficking patterns [5, 6].

10X single-cell V(D)J sequencing is a powerful tool for investigating paired heavy and light chain repertoires of B cell immunoglobulins [7]. It has been used in the identification of neutralizing antibodies against severe acute respiratory syndrome coronavirus 2 (SARS-CoV-2) [8], the virus that causes coronavirus disease 2019 (COVID-19) [9]. However, accurately analyzing 10X single-cell immunoglobulin repertoires remains a challenge due to the high diversity of immunoglobulin repertoires and the lack of specialized software that can analyze such diverse data.

Here, we developed SCIGA (Single-Cell Immunoglobulin Repertoire Analysis), a software for quickly analyzing the data of 10X single-cell immunoglobulin repertoires. SCIGA performs read trimming, immunoglobulin sequence assembly and annotation, heavy and light chain pairing by a one-line command. It also computes the statistics of repertoires, including gene usage frequency, SHM rate, length of complementarity determining region 3 (CDR3), and

clonality, and further implements visualization. We profiled the immunoglobulin repertoires of peripheral blood mononuclear cells (PBMCs) from nine COVID-19 patients using SCIGA. Finally, we identified four neutralizing antibodies against SARS-CoV-2 from these repertoires.

Methods

SCIGA is a software for the analysis of 10X single-cell immunoglobulin repertoires. It integrates several tools and algorithms into a single workflow. The input data can be raw reads or the output of Cell Ranger [10]. The details of the SCIGA algorithm can be found in the Supplementary Methods and Materials. Briefly, the workflow, which is summarized in Fig. 1, is as follows: 1) Quality control of reads. Trim the reads of low-quality using Trimmomatic [11]; 2) Call cell. The 10X system generates a large amount of Gel Beads-in-Emulsion (GEMs) that contain no cell. We need to identify the cell-containing GEMs before further analysis. SCIGA considers the GEMs containing cell(s) when the read number of the GEMs is over a threshold (see the Supplementary Methods and Materials); 3) Immunoglobulin sequence assembly. The immunoglobulin sequences for each cell were assembled using SSAKE [12], which is a reliable de novo assembler for short reads; 4) Gene call. To detect the usage of the V(D)J gene and C gene (isotype), SCIGA aligns the assembled immunoglobulin sequences against the V-, D-, and J- gene reference database using IgBLAST [13] and against C-gene reference database using BLAST [14]. The V(D)JC

reference databases for humans, mice and rats were downloaded from the international immunogenetics information system (IMGT) [15] and embedded in the SCIGA software; 5) Quality control of the immunoglobulin sequence. Only the immunoglobulins that are complete, in the correct reading frame and have no stop codon are retained; 6) Quality control of the cells. After immunoglobulin sequence assembly and filtering, some cells have multiple heavy or light chains, whereas some cells have only one chain. SCIGA reports the heavy and light chain with the highest number of unique molecular identifiers (UMIs) for each cell. A certainty score is calculated for each reported chain (see the Supplementary Methods and Materials). The chains with a certainty score less than a given threshold are discarded. Next, the cells without paired heavy and light chains are filtered out; 7) Clonal lineage grouping. Clonal lineage is defined as the cells that have identical V_H, J_H, V_L and J_L genes, identical H-CDR3 length, and over a given similarity threshold of H-CDR3 nucleotide sequences; 8) Statistical analysis and visualization. SCIGA calculates a list of statistics, including gene usage frequency, SHM rate, CDR3 length, Simpson index, Shannon entropy, and others. SCIGA subsequently generates figures to show the features of the repertoires. 9) Multiple sample integration analysis. After analyzing each sample, SCIGA consolidates all of the outputs into one. It identifies the shared immunoglobulins that are potential public antibodies suitable for use against a specific pathogen. Shared immunoglobulins are defined as immunoglobulins from different samples that can be clustered into

the same clonal lineage. Clustering is performed as described in step 7 with cells of all samples.

Results

Comparing SCIGA to existing software

At the time of this study, Cell Ranger is the only existing software for processing raw data generated by 10X single-cell V(D)J sequencing. A test dataset was therefore built to compare SCIGA to Cell Ranger. The PBMCs from nine COVID-19 patients (B1 to B9) were collected and 10X single-cell V(D)J sequencing was performed (Fig. 2A and Table S1). The raw data were analyzed using SCIGA and Cell Ranger (v3.1.0) with the default parameters. The comparison mainly focused on the following aspects: 1) Cell quality control. For Cell Ranger, the final results still included low-quality cells that either had multiple heavy or light chains, or only one chain. In our test dataset, the percentage of the low-quality cells reached an average of 29% (16.8% to 47.6% per sample, Fig. 2B). SCIGA implements the cell quality control process (step 6 described in Methods) and only outputs the high-quality cells. The cell count was generally less in the output of SCIGA compared to Cell Ranger due to the strict quality control process (Fig. 2C); 2) Detecting B cell clonal lineage. Cell Ranger clusters B cells into a clonal lineage when cells have identical nucleotide sequences of CDR3. However, it will break up the clonotypes that are clonally related in fact when the SHM falls within the CDR3 region. SCIGA

uses a popular clonal grouping method (step 7 of Methods), which considers the SHM and allows mismatch in the CDR3 region. Therefore, SCIGA could detect a larger clonal lineage than Cell Ranger (Fig. 2D); 3) Output information. The output of Cell Ranger is quite limited and some important information, such as SHM rate, is not included. SCIGA outputs the necessary information, including gene frequency, clone frequency, clonality, SHM rate, CDR3 length, the immunoglobulin variable region sequence, and others (Fig. 2E). Moreover, SCIGA is able to implement visualization to display the features of the repertoires; 4) Detecting shared immunoglobulin. This is a specific function in SCIGA and it could detect the shared immunoglobulin across samples.

Determining the features of immunoglobulin repertoires of COVID-19 using SCIGA

We performed a trial study to show the usage and performance of SCIGA. The 10X V(D)J sequencing data of the nine COVID-19 patients were analysis and features of the immunoglobulin repertoires were determined. A total of 8,358 B cells were detected (571-2,371 cells per sample, Fig. 2C). We focused on the genes used in at least 1% of B cells for the V-gene usage (Fig. 3A and Fig. S1). The top three gene families were IGHV4-34 (12.51%), IGHV3-30 (7.95%), and IGHV3-23 (6.30%) for the heavy chain, and IGLV3-19 (9.46%), IGKV1-39 (8.26%), and IGKV3-20 (7.90%) for the light chain. IGHV4-34 and IGLV3-19 had elevated usage frequency in the repertoire of patient B2, and reached 63.98% and 64.39%, respectively. IGHM had the highest usage in the

repertoires of most patients, except for patient B2, who showed the highest usage of IGHG1 (Fig. 3B). The SHM levels were low in the repertoires of most patients (<2%, Fig. 3C and Fig. S2). However, patient B2 showed a high SHM level in the IGH chain (7.48%) and IGL chain (7.13%). Moreover, patient B2 had an elevated CDR3 length for its immunoglobulin repertoire (Fig. 3D and Fig. S3).

Clonal lineage analysis using SCIGA

Clonal lineages were grouped using SCIGA with the default parameter. We used the Simpson index and Shannon entropy to determine the clonality of the immunoglobulin repertoires (Fig. 4A and 4B). Both indices showed that the repertoires of patient B2 experienced clonal expansion. The top 10 largest clones of each patient were reviewed (Fig. 4C and Fig. S4). The frequency of the largest clone for most patients was below 8%. However, the largest clone in patient B2 reached a frequency of 61.21%. This strongly expanded clone used the IGHV4-34 and IGLV3-19 genes, with an 8.82% mean SHM rate and 23-amino acid H-CDR3 length. We determined the most used V-genes in the top 10 largest clones of all patients. It was observed that IGHV4-34 (nine clones) was the most used gene in the heavy chain, IGKV1-39 (nine clones) and IGKV3-20 (nine clones) were the most used genes in the light chain, and IGHV4-34: IGLV3-19 (five clones) was the most used gene pair (Fig. S5A and S5B and S5C). Next, the shared immunoglobulin sequences across patients were determined using SCIGA. There were 12 immunoglobulins shared

between patient B1 and B2, 1 immunoglobulin was shared between patient B5 and B8, and 26 immunoglobulins were shared between patient B6 and B9 (Fig. 4D and Table S2).

Identification of neutralizing monoclonal antibodies

It was hypothesized that IgGs with higher clonal expansion may be SARS-CoV-2-specific antibodies in the COVID-19 patients. Thus, monoclonal antibodies (mAbs) were screened for the following criteria: IgG antibodies in the clone with fraction ⩾ 1% and cell number ⩾ 20 (see the Supplementary Methods and Materials). Four mAbs met the criteria and were expressed: B2-C1, B6-C2, B6-C3, and B8-C1 (Fig. 4C and Table S3). Remarkably, enzyme-linked immunosorbent assay (ELISA) revealed that all four mAbs were SARS-CoV-2 RBD (receptor binding domain) specific antibodies, which bound to the extracellular domain (ECD), the S1 subunit, and the RBD of the SARS-CoV-2 spike (Fig. 5A). They did not bind to the N-terminal domain (NTD) and the S2 subunit. The monoclonal antibodies could neutralize SARS-CoV-2 by blocking the attachment of RBD to the receptor (angiotensin-converting enzyme 2, ACE2) on host cells. B2-C1 and B6-C3 exhibited potent neutralizing activity [half-maximal inhibitory concentrations (IC_{50}) = 0.75 μg/ml and 0.32 μg/ml, respectively] against SARS-CoV-2 pseudovirus, whereas B8-C1 (1.47 μg/ml) and B6-C2 (14.89 μg/ml) were moderate and weak neutralizing antibodies (Fig. 5B). Similar results were found for the neutralization of the four mAbs against SARS-CoV-2 live virus (Fig. 5C).

Discussion

In this study, we developed the SCIGA software for 10X single-cell immunoglobulin repertoire analysis. SCIGA is an easy-to-use software and allows researchers to quickly perform advanced analysis on 10X V(D)J sequencing datasets. Cell Ranger has previously been used for 10X single-cell immunoglobulin repertoire analysis. However, this software includes low-quality cells in the output and disregards the effect of SHM when defining clonal lineage. In addition, some important information about repertoires, including the level of SHM for example, is not included in the output of Cell Ranger. SCIGA performs the quality control process for cells and defines the clonal lineage including the effect of SHM. Larger clones can be detected by using SCIGA. Moreover, SCIGA generates the needed statistical outputs and implements visualization. It is therefore a more efficacious tool for researchers.

In this study, SCIGA was used to analyze the single-cell immunoglobulin repertoires of the PBMCs of COVID-19 patients. Large-scale clone expansion was not observed in most patients. In patient B2, however, B cells expanded, which was indicated by a large size of clonal lineage with the IgG isotype. This indicates that patient B2 likely generated neutralizing antibodies against SARS-CoV-2.

Finally, we tried to identify the SARS-CoV-2-responding antibodies. In previous work, immunoglobulins with an SHM rate lower than 2% were excluded in screens for neutralizing antibodies [8]. However, some studies have shown that

several potent neutralizing antibodies against SARS-CoV-2 have low SHM rates [16-18]. Therefore, we included the antibodies with low SHM levels in our work. Four neutralizing antibodies with different potency were identified using our criteria. This demonstrates that SCIGA is useful for 10X single-cell immunoglobulin repertoire analysis.

List of abbreviations

ACE2: angiotensin-converting enzyme 2

CDR3: complementarity determining region 3

COVID-19: coronavirus disease 2019

ECD: extracellular domain

GEMs: Gel Beads-in-emulsion

IC50: half-maximal inhibitory concentration

Ig: immunoglobulin

mAbs: monoclonal antibodies

NTD: N-terminal domain

PBMCs: peripheral blood mononuclear cells

RBD: receptor binding domain

SARS-CoV-2: severe acute respiratory syndrome coronavirus 2

SHM: somatic hypermutation

UMI: unique molecular identifier

Availability of supporting source code and requirements

Project name: SCIGA (RRID: SCR_021002, Biotools ID: sciga)

Project home page: https://github.com/sciensic/SCIGA

Operating system(s): Linux

Programming language: Perl

Other requirements: IgBlast 1.15.0 or higher, Blast 2.9.0 or higher, R (optional), ggplot2 (optional)

License: GNU GPL-3.0 License

Availability of supporting data

The data set(s) supporting the results of this article is(are) available in the [National Center for Biotechnology Information] repository (https://www.ncbi.nlm.nih.gov/), [PRJNA682839].

Ethics, consent and permissions

This study was conducted according to the ethical principles of the Declaration of Helsinki. Ethical approval was obtained from the Research Ethics Committee of Shenzhen Third People's Hospital (2020-207). All participants provided written informed consent for sample collection and subsequent analyses.

Authors' contributions

Z.Z. designed this study and wrote the manuscript. H.Y. performed this study

and wrote the manuscript. L.C. performed the antibody neutralization test and wrote the manuscript. B.J. performed the ELISA test. G.X. performed the 10X single-cell V(D)J sequencing. Y.L. revised the manuscript.

Competing interests

The authors declare that they have no competing interests.

Funding

This study was supported by the National Science Fund for Distinguished Young Scholars (82025022), the Sanming Project for Medicine of Shenzhen (SZSM201612053), the National Key Plan for Scientific Research and Development of China (2020YFC0848800, 2020YFC0844200), the National Science and Technology Major Project of the Infectious Diseases (2018ZX10301404 to ZZ), the Science and Technology Innovation Committee of Shenzhen Municipality (202002073000002, 2020A1111350032, JCYJ20190809115617365), the National Natural Science Foundation of China (82002140) and the Natural Science Foundation of Guangdong Province of China (2019A1515011197).

Acknowledgements

We thank LetPub (www.letpub.com) for its linguistic assistance during the preparation of this manuscript.

References

1. V, G., et al., - *Bioinformatic and Statistical Analysis of Adaptive Immune Repertoires.* - Trends Immunol. 2015 Nov;36(11):738-749. doi: 10.1016/j.it.2015.09.006. Epub 2015, (- 1471-4981 (Electronic)): p. - 738-749.
2. FA, T., et al., - *Biased IGH VDJ gene repertoire and clonal expansions in B cells of chronically.* - Blood. 2018 Feb 1;131(5):546-557. doi: 10.1182/blood-2017-09-805762. Epub 2017 Dec, (- 1528-0020 (Electronic)): p. - 546-557.
3. SCA, N., et al., - *Shaping of infant B cell receptor repertoires by environmental factors and.* - Sci Transl Med. 2019 Feb 27;11(481):eaat2004. doi: 10.1126/scitranslmed.aat2004., (- 1946-6242 (Electronic)): p. T - ppublish.
4. A, N., et al., - *Fierce Selection and Interference in B-Cell Repertoire Response to Chronic HIV-1.* - Mol Biol Evol. 2019 Oct 1;36(10):2184-2194. doi: 10.1093/molbev/msz143., (- 1537-1719 (Electronic)): p. - 2184-2194.
5. H, R., - *Immunosequencing: applications of immune repertoire deep sequencing.* - Curr Opin Immunol. 2013 Oct;25(5):646-52. doi: 10.1016/j.coi.2013.09.017. Epub 2013, (- 1879-0372 (Electronic)): p. - 646-52.
6. JN, S., et al., - *B cells populating the multiple sclerosis brain mature in the draining cervical.* - Sci Transl Med. 2014 Aug 6;6(248):248ra107. doi: 10.1126/scitranslmed.3008879., (- 1946-6242 (Electronic)): p. - 248ra107.
7. LD, G., et al., - *Massively parallel single-cell B-cell receptor sequencing enables rapid discovery of.* - Commun Biol. 2019 Aug 9;2:304. doi: 10.1038/s42003-019-0551-y. eCollection 2019., (- 2399-3642 (Electronic)): p. - 304.
8. Y, C., et al., - *Potent Neutralizing Antibodies against SARS-CoV-2 Identified by High-Throughput.* - Cell. 2020 Jul 9;182(1):73-84.e16. doi: 10.1016/j.cell.2020.05.025. Epub 2020 May, (- 1097-4172 (Electronic)): p. - 73-84.e16.
9. D, W., et al., - *The SARS-CoV-2 outbreak: What we know.* - Int J Infect Dis. 2020 May;94:44-48. doi: 10.1016/j.ijid.2020.03.004. Epub 2020 Mar, (- 1878-3511 (Electronic)): p. - 44-48.
10. Cell Ranger: https://support.10xgenomics.com/single-cell-gene-expression/software/downloads/latest?
11. AM, B., L. M, and U. B, - *Trimmomatic: a flexible trimmer for Illumina sequence data.* - Bioinformatics. 2014 Aug 1;30(15):2114-20. doi: 10.1093/bioinformatics/btu170. Epub, (- 1367-4811 (Electronic)): p. - 2114-20.
12. RL, W., et al., - *Assembling millions of short DNA sequences using SSAKE.* - Bioinformatics. 2007 Feb 15;23(4):500-1. doi: 10.1093/bioinformatics/btl629. Epub, (- 1367-4811 (Electronic)): p. - 500-1.
13. J, Y., et al., - *IgBLAST: an immunoglobulin variable domain sequence analysis tool.* - Nucleic Acids Res. 2013 Jul;41(Web Server issue):W34-40. doi: 10.1093/nar/gkt382., (- 1362-4962 (Electronic)): p. - W34-40.
14. C, C., et al., - *BLAST+: architecture and applications.* - BMC Bioinformatics. 2009 Dec 15;10:421. doi: 10.1186/1471-2105-10-421., (- 1471-2105 (Electronic)): p. - 421.
15. MP, L., et al., - *IMGT®, the international ImMunoGeneTics information system® 25 years on.* - Nucleic Acids Res. 2015 Jan;43(Database issue):D413-22. doi: 10.1093/nar/gku1056.,

(- 1362-4962 (Electronic)): p. - D413-22.

16. C, K., et al., - *Longitudinal Isolation of Potent Near-Germline SARS-CoV-2-Neutralizing Antibodies.* - Cell. 2020 Aug 20;182(4):843-854.e12. doi: 10.1016/j.cell.2020.06.044. Epub 2020 Jul, (- 1097-4172 (Electronic)): p. - 843-854.e12.
17. B, J., et al., - *Human neutralizing antibodies elicited by SARS-CoV-2 infection.* - Nature. 2020 Aug;584(7819):115-119. doi: 10.1038/s41586-020-2380-z. Epub 2020 May, (- 1476-4687 (Electronic)): p. - 115-119.
18. TF, R., et al., - *Isolation of potent SARS-CoV-2 neutralizing antibodies and protection from disease.* - Science. 2020 Aug 21;369(6506):956-963. doi: 10.1126/science.abc7520. Epub 2020 Jun, (- 1095-9203 (Electronic)): p. - 956-963.
19. I, L., et al., - *BraCeR: B-cell-receptor reconstruction and clonality inference from single-cell.* - Nat Methods. 2018 Aug;15(8):563-565. doi: 10.1038/s41592-018-0082-3., (- 1548-7105 (Electronic)): p. - 563-565.
20. N, C. and W. DR, - *Analyzing Immunoglobulin Repertoires.* - Front Immunol. 2018 Mar 14;9:462. doi: 10.3389/fimmu.2018.00462. eCollection 2018., (- 1664-3224 (Print)): p. - 462.
21. L, Z., et al., - *Lineage tracking reveals dynamic relationships of T cells in colorectal cancer.* - Nature. 2018 Dec;564(7735):268-272. doi: 10.1038/s41586-018-0694-x. Epub 2018 Oct, (- 1476-4687 (Electronic)): p. - 268-272.

Figure legend

Fig. 1 The workflow of SCIGA. The workflow includes quality control of reads, call cell, immunoglobulin (Ig) sequence assembly, V(D)JC gene call, quality control of Ig sequence, cell quality control, group clonal lineage, statistical analysis and visualization, and multiple sample integration analysis.

Fig. 2 Comparison of SCIGA and Cell Ranger. (A) Flowchart of the experiment. **(B)** The percentage of low-quality cells in the output of Cell Ranger. Single denotes the cells containing single chain. Multiple denotes the cells containing multiple heavy or light chains. **(C)** The count of B cells in the output of SCIGA and Cell Ranger. **(D)** The fraction of the top 10 largest clone analyzed using SCIGA and Cell Ranger. **(E)** The output information of the SCIGA and

Cell Ranger.

Fig. 3 The features of the single-cell immunoglobulin repertoires of nine COVID-19 patients. (A) The average usage frequency of V-genes in the repertoires. Only show the genes with frequency > 1%. **(B)** The usage frequency of isotypes in the repertoire of each patient. Colors denote the isotypes. **(C)** The mean SHM rate of V-genes **(D)** The mean CDR3 length in the repertoire of each patient, with IGH shown in red, IGK in yellow and IGL in blue. The error bars represent the standard error.

Fig. 4 The B cell clonal expansion of nine COVID-19 patients. (A) The Simpson index **(B)** The Shannon entropy denote the clonality of the repertoire of each patient. **(C)** The top 10 largest clones in the repertoire of each patient. The x-axis captures the clone ID and used V-genes. The initial of the gene names denote the chain, with H is IGH, K is IGK, and L is IGL. * denotes the screened antibody candidate. **(D)** Number of immunoglobulins shared between patients. Blank means zero.

Fig. 5 Characteristics of the spike specific monoclonal antibodies. (A) The binding profile of selected monoclonal antibodies to the extracellular domain and subdomains of the SARS-CoV-2 spike by ELISA. HIV-1-GP140 is the negative control. **(B-C)** Neutralization activity of selected monoclonal antibodies

against the pseudovirus (**B**) and live SARS-CoV-2 (**C**). The dashed line indicates a 50% reduction in viral infectivity. Human IgG1 is the negative control. Results presented here are the representative of two independent experiments.

Fig. S1 Frequency of V-genes in the repertoire of each patient. Only the genes with a frequency > 1% are shown.

Fig. S2 Distribution of the SHM rate in the repertoire of each patient. Color denotes the chain, with IGH shown in red, IGK in yellow and IGL in blue.

Fig. S3 Distribution of the CDR3 length in the repertoire of each patient. Color denotes the chain, with IGH shown in red, IGK in yellow and IGL in blue.

Fig. S4 Fraction of all clones in the repertoire of each patient. The x-axis captures the clone rank and y-axis captures the clone fraction. * denotes the selected antibody candidate.

Fig. S5 Number of the used V-genes of the top 10 largest clones of all patients. (A) The count of the used V-gene for the heavy chain. **(B)** The count of the used V-gene for the light chain. **(C)** The count of the used V-gene pair. The initial of the gene names denote the chain, with H is IGH, K is IGK, and L is IGL.

Fig. S6 Two different examples show how to chose the threshold. The example having **(A)** and not having **(B)** large difference in the read counts between cells-containing GEMs and background.

Table. S1 Information of the nine COVID-19 patients.

Table. S2 Information of the shared immunoglobulins. The columns 7-11 denote the number of shared immunoglobulin sequences.

Table. S3 Information of the four antibody candidates.

Supplementary Methods and Materials

Note 1. Algorithm of SCIGA

Note 2. Sample processing and sequencing

Note 3. Data analysis

Note 4. Identification of the monoclonal antibody

Note 5. Nucleotide sequences of the antibody candidates

Note 1. Algorithm of SCIGA

Quality control of reads

SCIGA trims low-quality reads using Trimmomatic which is embedded in the SCIGA software. SCIGA allows users to set up the criteria for quality control, including the size of the sliding window for trimming reads (default value is 4), the cut off value for the average quality score in a sliding window (default value is 15), and the cut off for the length of reads after trimming (default value is 75).

Cell calling

In the 10X system, the majority (~90–99%) of generated GEMs contain no cell. We need to detect the cell-containing GEMs base on the read counts. SCIGA trims the first 39 bases of read 1 containing the 16-nt cell barcode, 10-nt unique molecular identifier (UMI) and 13-nt switch oligonucleotide as described previously [7]. The barcode and UMI are retained for each read. Reads with identical cell barcodes are considered as being derived from the same cell. SCIGA calculates the read number per barcode and ranks these barcodes by read number in reverse order. The barcodes not in the top 10% are discarded, since at least 90% of GEMs contain no cell. The barcodes with read number over a given threshold are retained. SCIGA provides two methods to help choose the threshold as follows: 1) SCIGA is used to construct a curve where the rank of barcodes is used as x-axis information and the read count of the barcode serves as y-axis information. The threshold is set at the point where the gradient is minimal. This method is suitable for samples having large differences in read counts between “real” cells and background (Fig. S6A); 2) The threshold is set arbitrarily (default value is 200) (Fig. S6B).

Immunoglobulin sequence assembly

If the read number for a given barcode exceeds 80,000, it is downsampled to 80,000. SCIGA performs immunoglobulin assembly for each barcode separately using SSAKE, a reliable de novo assembler for short reads that is embedded in the SCIGA software. The trimmed reads 1 and reads 2 are used as input for SSAKE with the parameter “-w 5 -p 1 -c 1”. Contiguous sequences with length less than 300 bases or coverage less than a given threshold (default value is 3) are discarded.

Making a reference database

A reference database (embedded in SCIGA) is needed before determining the usage of the V-, (D-), J- gene of the assembled immunoglobulin sequences. The ungapped nucleotide sequences of all V-, D-, J-, and C-gene segments of heavy, kappa, and lambda chains were downloaded from the international immunogenetics information system (www.imgt.org). The reference database contained human, mouse, and rat sequences. Most C-genes had identical gene names but had different sequences. Tags were added to the gene names to distinguish them from each other. The indexes of V-, D-, J- sequences were built by IgBLAST and indexes of C- sequences were built by BLAST.

V(D)JC gene calling

The usage of the V-, (D-), J- genes are determined. For this purpose, SCIGA aligns the high-quality contiguous sequences against the V-, D-, J- gene reference database using IgBLAST with the parameter “-evalue 0.001”. To

determine the isotype usage, SCIGA aligns the contiguous sequences against the C-gene reference database using BLAST with the parameter "-evalue 0.001". SCIGA only retains the alignments with the highest score.

Quality control of immunoglobulin sequences

SCIGA sets up several quality-control steps to obtain complete V(D)J sequences, as follows: 1) The V(D)J sequences that cannot be assigned to certain V- or J- genes are discarded; 2) The V(D)J sequences that fail to identify the CDR3 region are discarded; 3) The V(D)J sequences must be in the correct reading frame and have no stop codon; 4) The V(D)J nucleotide sequences should be aligned to the first position of the V-gene to ensure the intactness of the FR1 region. 5) The V(D)J amino sequences should include the first four positions of the FR4 region, as previously defined [19], to ensure the intactness of the CDR3 region. The first four positions should be the conserved motifs of XGXG, WSQG (heavy chain), FGXG (light chains), or FSDG (kappa chain).

B cell quality control

Typically, a B cell has one heavy and one light chain. However, after immunoglobulin sequence assembly and quality control, some cells have multiple heavy or light chains (This may be due to the contamination of free RNA or multiple cells in GEMs). The other cells have only one chain which may be due to the low sequencing depth. For each cell, SCIGA reports the heavy and light chain with the highest UMIs number. For each reported chain, SCIGA calculates a certainty score, which is defined as the number of UMIs supporting

the chain divided by the total number of UMIs of all heavy or light chains [7]. The chains with a certainty score less than a given threshold (default value is 80%) are discarded. Finally, the cells without paired heavy and light chains are filtered out.

Clonal lineage grouping

SCIGA defines the cells as clonal lineage when the cells have identical V_H, J_H, V_L and J_L genes, identical H-CDR3 length, and over a given similarity threshold (default value is 90%) of H-CDR3 nucleotide sequences [7]. SCIGA implements this step by using a custom script as follows: 1) Group the cells with identical V_H, J_H, V_L and J_L genes and identical H-CDR3 length into a cluster; 2) Merge the identical H-CDR3 nucleotide sequences for each cluster into a unique representative sequence and calculate the abundance of the representative sequence. Next, rank the representative sequences by abundance in the reverse order. 3) Perform an iteration process: the first representative sequence serves as the centroid of the first clone. Next sequentially compare the given nucleotide sequence to the centroids of all existing clones and calculate the identity scores. If the maximum identity score is more than a given similarity threshold (default value is 90%), SCIGA assigns the given sequence to the clone with the maximum identity score, or assigns it to a new clone as the centroid.

Statistical analysis and visualization

SCIGA computes a list of statistics. Some of them are calculated as below:

Gene usage frequency is calculated as $\frac{gene\ usage\ count}{total\ cell\ count} \times 100\%$.

SHM rate of the V(D)J gene is calculated as $\frac{mismatchs\ in\ gene}{gene\ length} \times 100\%$.

Simpson index is calculated as $\frac{\sum_{i=1}^{S} n_i(n_i-1)}{N(N-1)}$, where n_i is the number of cells of the ith clone, N is the total number of cells, and S is the total number of clones [20].

Shannon entropy is calculated as $1 - \frac{-\sum_{i=1}^{S} p_i log_2 p_i}{log_2 S}$, where p_i is the fraction of the ith clone and S is the total number of clones [21].

For visualization of the repertoires, SCIGA generates several figures to show the distribution of the V-gene usage frequency, the SHM of V-gene, the CDR3 length, and the clone frequency by using the R programming language.

Multiple sample integration analysis

After analyzing each sample, SCIGA integrates the outputs of several samples into one and determines the shared immunoglobulin sequences. Shared immunoglobulins are defined as the immunoglobulins from different samples that can be clustered into the same clonal lineage. Clustering is performed with the cells of all samples.

Note 2. Sample processing and sequencing

PBMCs from COVID-19 convalescent patients were isolated using a Ficoll-Hypaque density gradient centrifugation protocol. The single-cell immunoglobulin (Ig) libraries were generated by using the Chromium Single Cell V(D)J Reagent Kits (10X Genomics; PN-1000006, PN-1000020, PN-

120236, PN-120262) following the manufacturer's instruction. Briefly, GEMs were generated by combining barcoded single cell 5’ gel beads, a master mix containing about 20,000 PBMCs, and partitioning oil onto chromium chip A. Reverse transcription takes place inside each GEM, which produces full-length cDNA from poly-adenylated mRNA. Next full-length cDNAs were amplified for V(D)J segment enrichment via PCR amplification with primers specific to Ig constant regions. Variable length fragments that collectively span the V(D)J segments of the enriched Ig transcripts were generated via enzymatic fragmentation for library construction. The resulting libraries that comprised standard Illumina paired-end constructs were sequenced.

Note 3. Data analysis

For the analysis of each sample, we used the SCIGA to process the paired-end reads generated by sequencing, with the default parameter. The code is following:

```
sciga -fq1 <read1.fastq.gz> -fq2 <read2.fastq.gz> -outdir <output> -species human
```

For the integration analysis of multiple samples, we used the SCIGA to process the results of multiple samples, with the default parameter. The code is following:

```
sciga-merge -in <B1, B2 … B9> -out <output>
```

For the analysis by using Cell Ranger, the code is following:

```
cellranger vdj --id=<sample_name> --fastqs=<fastq_directory>
```

--reference=<hg38_vdj> --sample=<sample_name> --denovo

Note 4. Identification of the monoclonal antibody

Screening the monoclonal antibody

Monoclonal antibodies were screened as follows: 1) Screen the clone with fraction ⩾ 1% and cell number ⩾ 20; 2) For each clone, screen the IgG immunoglobulin; 3) Trim the nucleotide sequences of immunoglobulin and retain the variable region; 4) Calculate the abundance of trimmed immunoglobulin sequences. The immunoglobulin sequence with the highest abundance in a clone is the monoclonal antibody candidate.

The expression and purification of monoclonal antibodies

The IgG heavy and light chain variable genes were synthesized and cloned into the human full-length IgG1 expression vectors (Sangon Biotech, Shanghai). Paired heavy- and light-chain expressing plasmids were co-transfected into 293 F cells, and antibodies were purified from the cell supernatants using protein A columns according to the manufacturer's instructions (National Engineering Research Center for Biotechnology, Beijing) after 5 days. The concentration of purified monoclonal antibodies was determined using a NanoDrop spectrophotometer (Thermo Scientific).

Enzyme-linked immunosorbent assay

The recombinant extracellular domain or other subdomains of SARS-CoV-2 S protein (spike, S1, RBD, NTD, and S2, all from Sino Biological, Beijing) were coated (2 μg/ml) onto 96-well plates overnight at 4°C. The plates were blocked

with the blocking buffer (phosphate buffered saline containing 5% skim milk and 2% bovine albumin) at RT for 1 h. Five-fold serial-diluted mAbs were added to the plates and subsequently incubated for 1 h at 37°C. HRP-conjugated goat anti-human IgG (ZSGB-BIO, Beijing) secondary antibody was added to the plates and incubated at 37°C for 1 h. The enzymatic reaction was developed with 3,3′,5,5′-tetramethylbenzidine (TMB) substrate (Kinghawk, Beijing) and stopped by addition of 2M H_2SO_4. The absorbance was measured at 450 nm using a Varioskan™ LUX Multimode Microplate Reader (Thermo Scientific). HIV-1-GP140 (purified in our lab) was an irrelevant antigen control.

Pseudovirus-based neutralization assay

The SARS-CoV-2 pseudovirus was generated through co-transfection of 293T cells with pVAX1-S and pNL4-3.Luc.R-E-, which carried the codon optimized SARS-CoV-2 S gene (GenBank: MN988668.1) and HIV-1 backbone, respectively. Viral supernatant was collected at 48 h post-transfection and frozen at -80°C. The serially diluted antibodies were incubated with equal volume pseudovirus at 37°C for 1 h. The antibody-virus mixtures were subsequently added onto 96-well plates which pre-seeded HEK 293T-ACE2 cells. After 48 h, infected cells were lysed to measure the luciferase activity using Bright-Glo Luciferase (Promega, Madison, WI) according to the manufacturer's protocol. The IC_{50} was determined by GraphPad Prism 7 using asymmetric (five parameters) model.

Focus reduction neutralization test

SARS-CoV-2 focus reduction neutralization test (FRNT) was performed in a certified Biosafety level 3 lab. Antibodies were 3-fold serially diluted and mixed with equal volume of SARS-CoV-2 live virus (containing 200 focus forming unit)

on U-bottom 96-well plates. The mixtures were incubated for 60 min at 37 ºC and next transferred onto the 96-well plate seeded with Vero E6 cells for 1 h at 37 ºC before removed. After washing, the overlay media (MEM containing 1.6% Carboxymethylcellulose, 2% fetal bovine serum) was added and cells were incubated at 37 ºC for 24 h. After removing the overlay media, cells were fixed with 4% paraformaldehyde solution, permeabilized with Perm/Wash buffer (BD Biosciences) containing 0.1% Triton X-100, incubated with HRP-conjugated anti-SARS-CoV-2-N IgG (isolated in our lab). The reactions were developed with KPL TrueBlue Peroxidase substrates (Seracare Life Sciences Inc). The numbers of SARS-CoV-2 foci were calculated using an EliSpot reader (Cellular Technology Ltd).

Note 5. Nucleotide sequences of the antibody candidates

Paired heavy and light chains of the antibodies are shown as follows:

>B2-C1_IGH

CAGGTGCAGCTACAGCAGTGGGGCGCGGGACTGTTGAAGCCTTCGGAGACCCTGT
CCCTCACCTGCGCTGTCTATGGTGTGTCGCCCACTAGTTACTATTGGAGCTGGATCC
GTCAGTCCCCCGGGAAGGGTCTGGAGTGGATAGGGGAGATCACTCATAGTGGAAGC
ACCAACTACAATCCGTCCCTCAAGAGTCGAGTCACCATGTCGCTGGACACGTCCAA
GAGCCAGTTCTCCCTGAAGTTGAGTTCTGTGACCGCCGCGGACACGGCTATATATTA
TTGTGCGAGGGGACGCAGTGAGGAGACCATGATAGTGATGGTTGTCACGGGAATTG
ATTTCTACTTTGACTCTTGGGGCCAGGGGACCCTGGTCACCGTCTCCTCA

>**B2-C1_IGL**

TCTTCTGAGCTGACTCAGGACCCTGCTGTGTCTGTGGCCTTGGGACAGACAGTCAGGATCACATGCCAAGGAGACAACCTCAAACTCTTTTATACAAACTGGTACCAGCAGAAGCCAGGCCAGGCCCCCGTACTTGTCATCCATGGTAAAAACAACCGGCCCTCAGGGATCCCAGACCGATTCTCTGGCTCCAGTTCAGCGTACACCACTTCCTTGACCATCATTGGGGCTCAGGCGGAGGATGAGGCTGACTATTACTGTAGCTCTCGCGACAGAAGTGGTGACCGTGTTATATTCGGCGGAGGGACCAAGGTGACTGTCCTA

>B6-C2_IGH

GAGGTGCTCCTGGTGGAGTCTGGGGGAGGCTTGGTCCGGCCTGGAGGGTCCCTAAGACTCTCCTGTGCAGCCTCTGGATTCACCTTCACTGACCACTATTTGGACTGGGTCCGCCAGGCTCCAGGGATGGGGCTGGAGTGGGTTGGCCGTATTAGAAATAAAGTTAATGGTTACACCACAGAATACGCCGCGTCTGTGAAAGGCAGATTCACCATCTCAAGAGATGATTCAAAGAACTCAGTTTATCTGCAAATGAATAGCCTGAGAAGCGAGGACACGGCCGTGTATTACTGCACTAGAGTGGGAGTTGGGAGCCCTGACTACTGGGGCCAGGGAACCCTGGTCGCCGTCTCCTCA

>B6-C2_IGK

GACATCCAGATGACCCAGTCTCCATCCTCCCTGTCTGCATCTGTAGGAGACAGAGTCACCATCACTTGCCGGGCAAGTCAGGGCATTAGAGATGAGTTAGCCTGGTATCAGCAAAAACCAGGGAAAGCCCCTAAGCGCCTGATCTATGATGCATCGAGGTTGCAAAGTGGGATCCCATCGAGGTTCAGCGGCAGTGGATCTGGGACAGAATTCACTCTCACAATCAGCAGTCTGCAGCCTGAAGATTTTGCAACTTATCATTGTCTACAGTATACTAGTTACCCTCACACTTTTGGCCAGGGGACCAAGCTGGACATCAAA

>B6-C3_IGH

CAGGTGCAGCTACAACAGTGGGGCGCAGGACTGTTGAAGCCTTCGGAGACCCTGT
CCCTCACCTGCGCTGTCTATGGTGGGTCCTTCAGTGGTTACCAGTGGAGGTGGATC
CGCCAGGCCCCAGGGAAGGGGCTGGAGTGGATTGGGGAAATCAATCATAGTGGAA
GCACCAATTACAACCCGTCCCTCAAGAGTCGAGTCACCATATCAGTAGACACGTCCA
AGAACCAGTTCTCCCTGAGGTTGAGGTCTGTGACCGCCGCGGACACGTCTGTGTAT
TTCTGTGCGAGAGGCCAAAATGGAGTAGTTCCAGCTCCTGTATTGGGGATCGGACCT
TACTACACCTACTCCTACATGGACGTCTGGGGCACAGGGACCACGGTCAGTGTCTC
CTCA

>B6-C3_IGL

TCTTCTGAGCTGACTCAGGACCCTGCTGTGTCTGTGGCCTTGGGACAGACAGTCAG
GATCACATGCCAAGGAGACAGCCTCAGAAGCTATTATGCAAGTTGGTACCAGCAGAA
GCCAAGACAGGCCCCTATTCTTGTCATCTATGGTAAAAACAATCGACCCTCAGGGATC
CCGGACCGATTCTCTGGCTCCTACTCAGGAGCCACAGCTTCCTTAACCATCACTGGG
GCTCAGGCGGAGGATGAGGCTGACTATTATTGTGACTCCCGGGACAGCAGTGGTAA
CCATCGAGTGTTCGGCAGAGGGACCACGGTGACCGTGCTA

>B8-C1_IGH

CAGGTGCAACTGGTGCAGTCTGGGGCTGAGGTGAAGAAGCCTGGGTCCTCGGTGA
GGGTCTCCTGCCAGGCTTCTGGAGACACCTTCAGCAACTATGCTTTCAGTTGGGTG
CGACAGGCCCCTGGACAAGGGCTTGAGTGGATGGGAAGGATCATCCCTATCTTTGG
AACACCAAACTACGCACAGAGGTTCCAGGGGAGAGTCACGATTACCGCGGACGAGT
CTACGAGGACAGCCTACATGGAATTGACCGGCCTGAGGTCTGACGACACGGCCGTG

TATTACTGTGCGAGACACACTTTGGTGACTGCTATTCAGAAGTGGGGCCAGGGAACC
CTGGTCACCGTCTCCTCA

>B8-C1_IGK

GACATCCAGATGACCCAGTCTCCTTCCACCCTGTCTGCGTCTGTTGGAGACAGAGT
CACCATCACTTGCCGGGCCAGTCAGAGTGTTAGTGACTGGTTGGCCTGGTATCAGC
AGAAACCAGGGGAGCCCCCTAAGCTCCTCATCTCTAGGGCATCTACTTTAGAGATTG
GGGTCGCATCAAGGTTCAGCGGCAGTGGATCTGGGACAGAATTCACTCTCACCATC
AGCAGCCTGCAGCCTGATGATTATGCAACTTATTACTGCCAACAGTATAATACTTATTC
GCTCACTTTCGGCGGAGGGACCAAGGTGGAGATCAAA

Figure 1

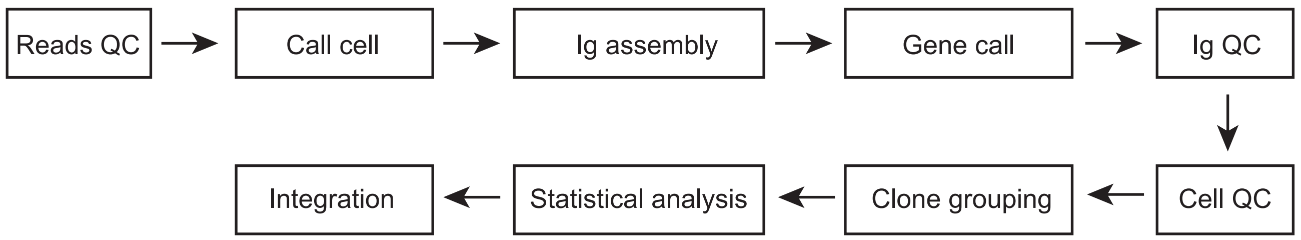

Reads QC
Call cell
Ig assembly
Gene call
Ig QC
Cell QC
Clone grouping
Statistical analysis
Integration

Figure 2

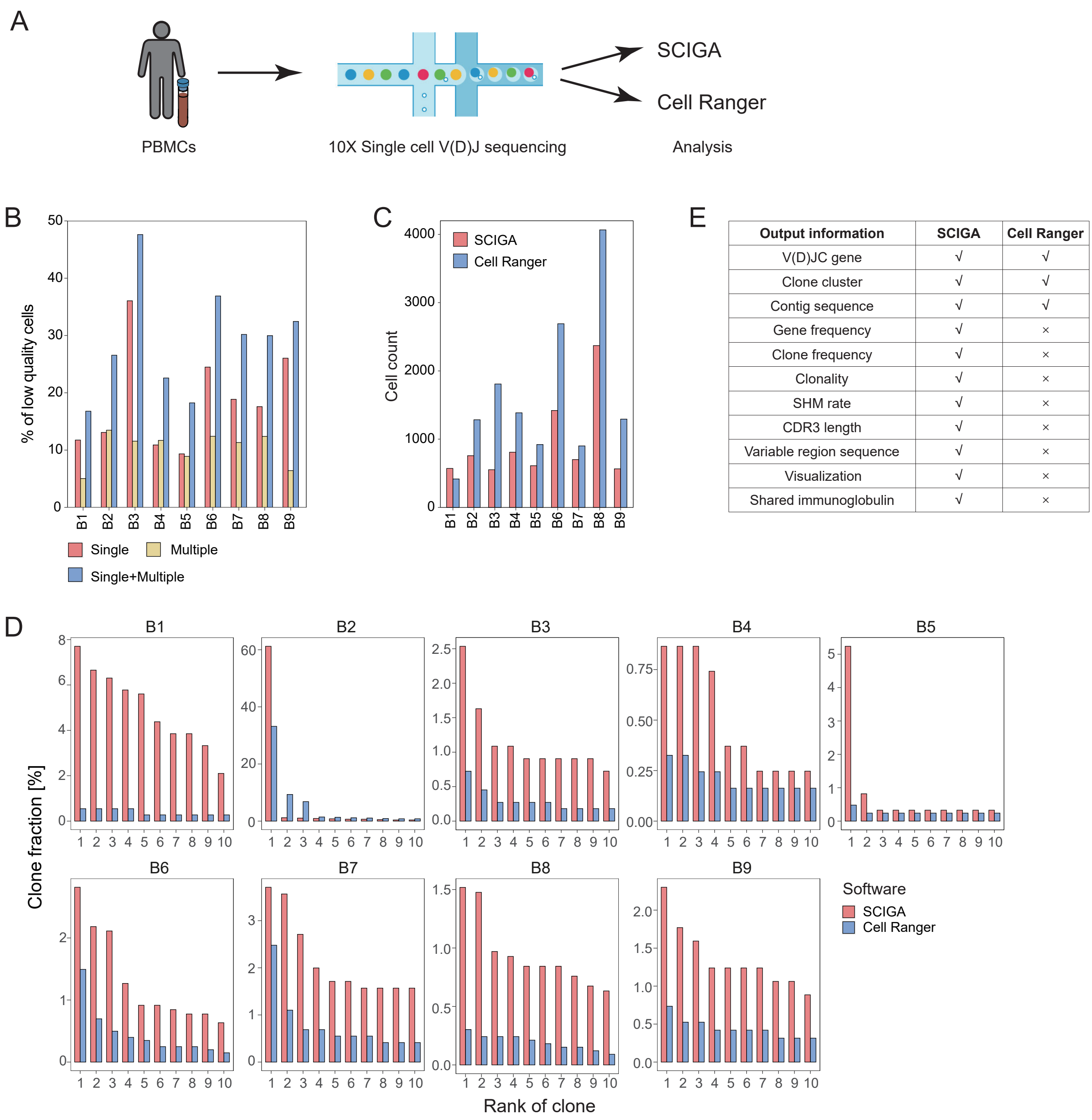

Output information	SCIGA	Cell Ranger
V(D)JC gene	√	√
Clone cluster	√	√
Contig sequence	√	√
Gene frequency	√	×
Clone frequency	√	×
Clonality	√	×
SHM rate	√	×
CDR3 length	√	×
Variable region sequence	√	×
Visualization	√	×
Shared immunoglobulin	√	×

Figure 3

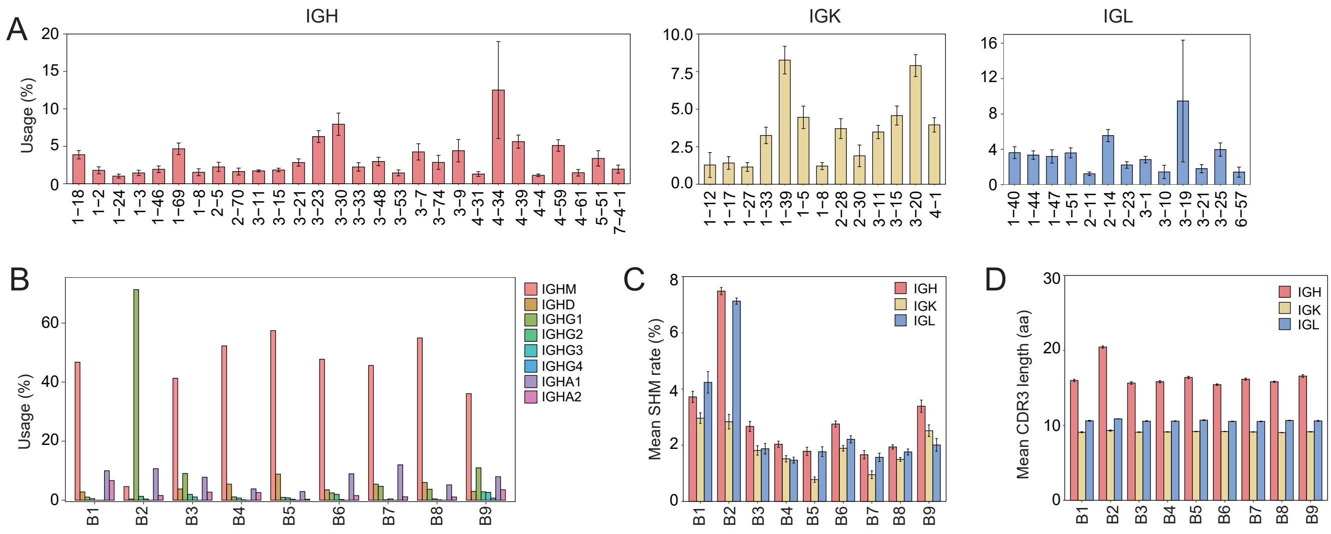
A
IGH
IGK
IGL
Usage (%)
B
Usage (%)
IGHM
IGHD
IGHG1
IGHG2
IGHG3
IGHG4
IGHA1
IGHA2
C
Mean SHM rate (%)
IGH
IGK
IGL
D
Mean CDR3 length (aa)
IGH
IGK
IGL
B1
B2
B3
B4
B5
B6
B7
B8
B9

Figure 4

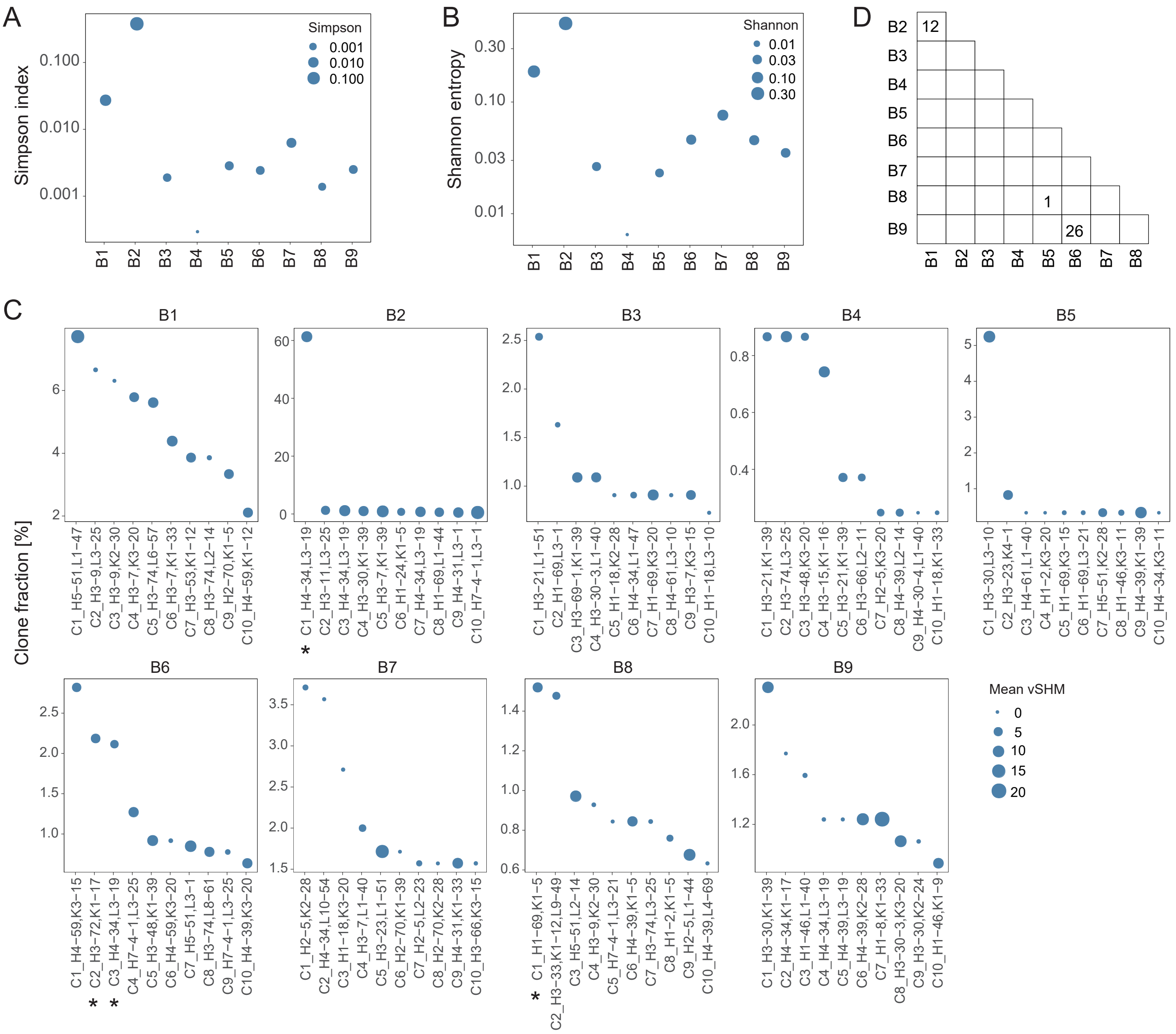
A
Simpson index
Simpson
0.001
0.010
0.100
B1 B2 B3 B4 B5 B6 B7 B8 B9
B
Shannon entropy
Shannon
0.01
0.03
0.10
0.30
D
B2 B3 B4 B5 B6 B7 B8 B9
B1 B2 B3 B4 B5 B6 B7 B8
12
1
26
C
Clone fraction [%]
B1
C1_H5−51,L1−47
C2_H3−9,L3−25
C3_H3−9,K2−30
C4_H3−7,K3−20
C5_H3−74,L6−57
C6_H3−7,K1−33
C7_H3−53,K1−12
C8_H3−74,L2−14
C9_H2−70,K1−5
C10_H4−59,K1−12
B2
C1_H4−34,L3−19
C2_H3−11,L3−25
C3_H4−34,L3−19
C4_H3−30,K1−39
C5_H3−7,K1−39
C6_H1−24,K1−5
C7_H4−34,L3−19
C8_H1−69,L1−44
C9_H4−31,L3−1
C10_H7−4−1,L3−1
B3
C1_H3−21,L1−51
C2_H1−69,L3−1
C3_H3−69−1,K1−39
C4_H3−30−3,L1−40
C5_H1−18,K2−28
C6_H4−34,L1−47
C7_H1−69,K3−20
C8_H4−61,L3−10
C9_H3−7,K3−15
C10_H1−18,L3−10
B4
C1_H3−21,K1−39
C2_H3−74,L3−25
C3_H3−48,K3−20
C4_H3−15,K1−16
C5_H3−21,K1−39
C6_H3−66,L2−11
C7_H2−5,K3−20
C8_H4−39,L2−14
C9_H4−30−4,L1−40
C10_H1−18,K1−33
B5
C1_H3−30,L3−10
C2_H3−23,K4−1
C3_H4−61,L1−40
C4_H1−2,K3−20
C5_H1−69,K3−15
C6_H1−69,L3−21
C7_H5−51,K2−28
C8_H1−46,K3−11
C9_H4−39,K1−39
C10_H4−34,K3−11
B6
C1_H4−59,K3−15
C2_H3−72,K1−17
C3_H4−34,L3−19
C4_H7−4−1,L3−25
C5_H3−48,K1−39
C6_H4−59,K3−20
C7_H5−51,L3−1
C8_H3−74,L8−61
C9_H7−4−1,L3−25
C10_H4−39,K3−20
B7
C1_H2−5,K2−28
C2_H4−34,L10−54
C3_H1−18,K3−20
C4_H3−7,L1−40
C5_H3−23,L1−51
C6_H2−70,K1−39
C7_H2−5,L2−23
C8_H2−70,K2−28
C9_H4−31,K1−33
C10_H3−66,K3−15
B8
C1_H1−69,K1−5
C2_H3−33,K1−12,L9−49
C3_H5−51,L2−14
C4_H3−9,K2−30
C5_H7−4−1,L3−21
C6_H4−39,K1−5
C7_H3−74,L3−25
C8_H1−2,K1−5
C9_H2−5,L1−44
C10_H4−39,L4−69
B9
C1_H3−30,K1−39
C2_H4−34,K1−17
C3_H1−46,L1−40
C4_H4−34,L3−19
C5_H4−39,L3−19
C6_H4−39,K2−28
C7_H1−8,K1−33
C8_H3−30−3,K3−20
C9_H3−30,K2−24
C10_H1−46,K1−9
Mean vSHM
0
5
10
15
20

Figure 5

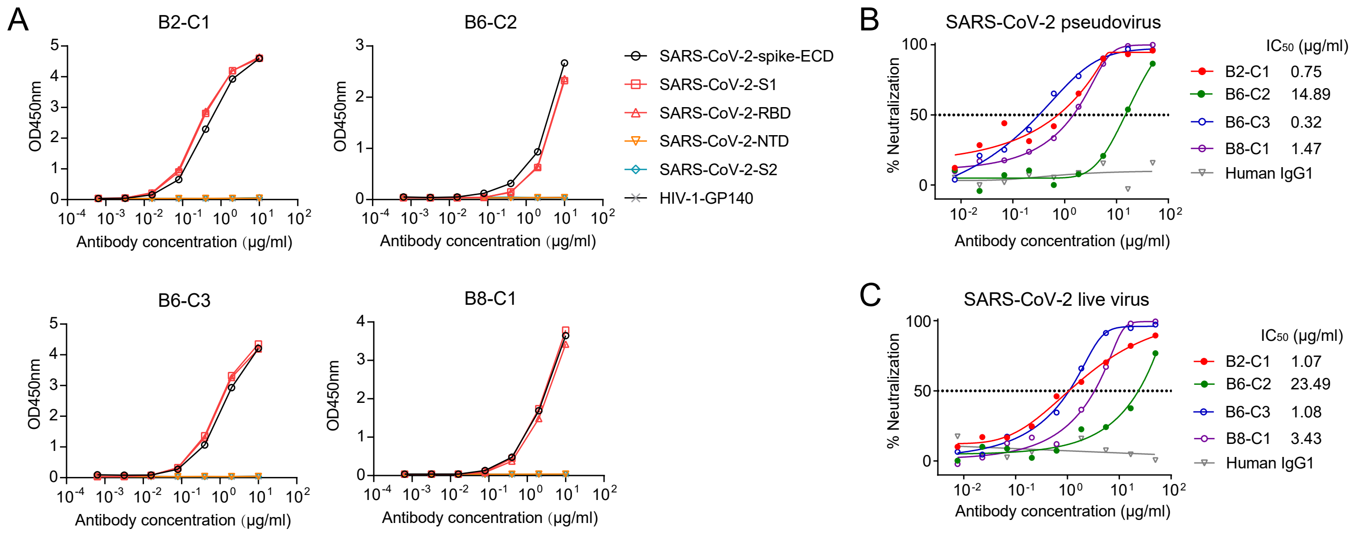

A
B2-C1
B6-C2
B6-C3
B8-C1
OD450nm
Antibody concentration (μg/ml)
SARS-CoV-2-spike-ECD
SARS-CoV-2-S1
SARS-CoV-2-RBD
SARS-CoV-2-NTD
SARS-CoV-2-S2
HIV-1-GP140
B
SARS-CoV-2 pseudovirus
% Neutralization
IC50 (μg/ml)
B2-C1 0.75
B6-C2 14.89
B6-C3 0.32
B8-C1 1.47
Human IgG1
C
SARS-CoV-2 live virus
IC50 (μg/ml)
B2-C1 1.07
B6-C2 23.49
B6-C3 1.08
B8-C1 3.43
Human IgG1

Figure S1

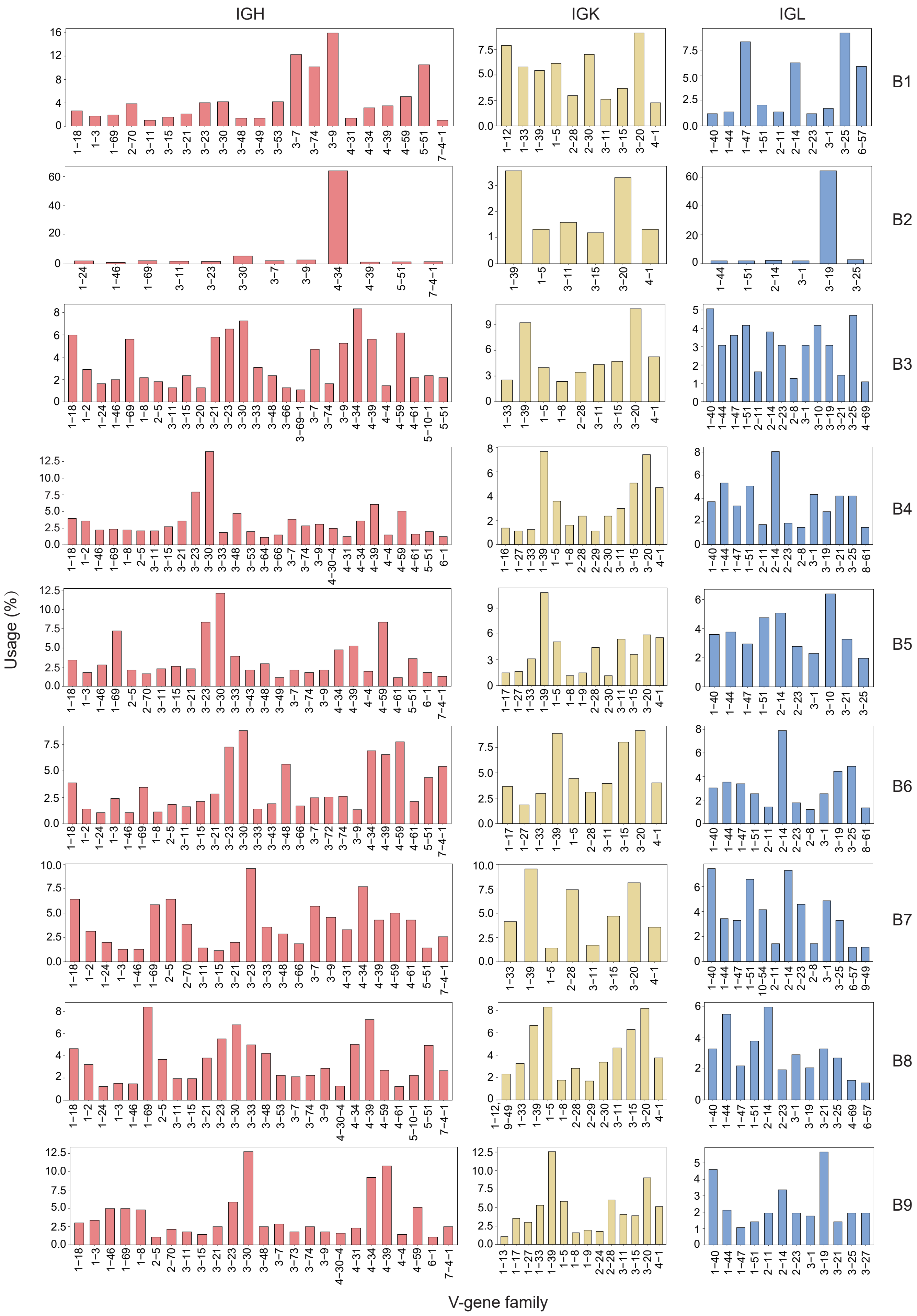

Figure S2

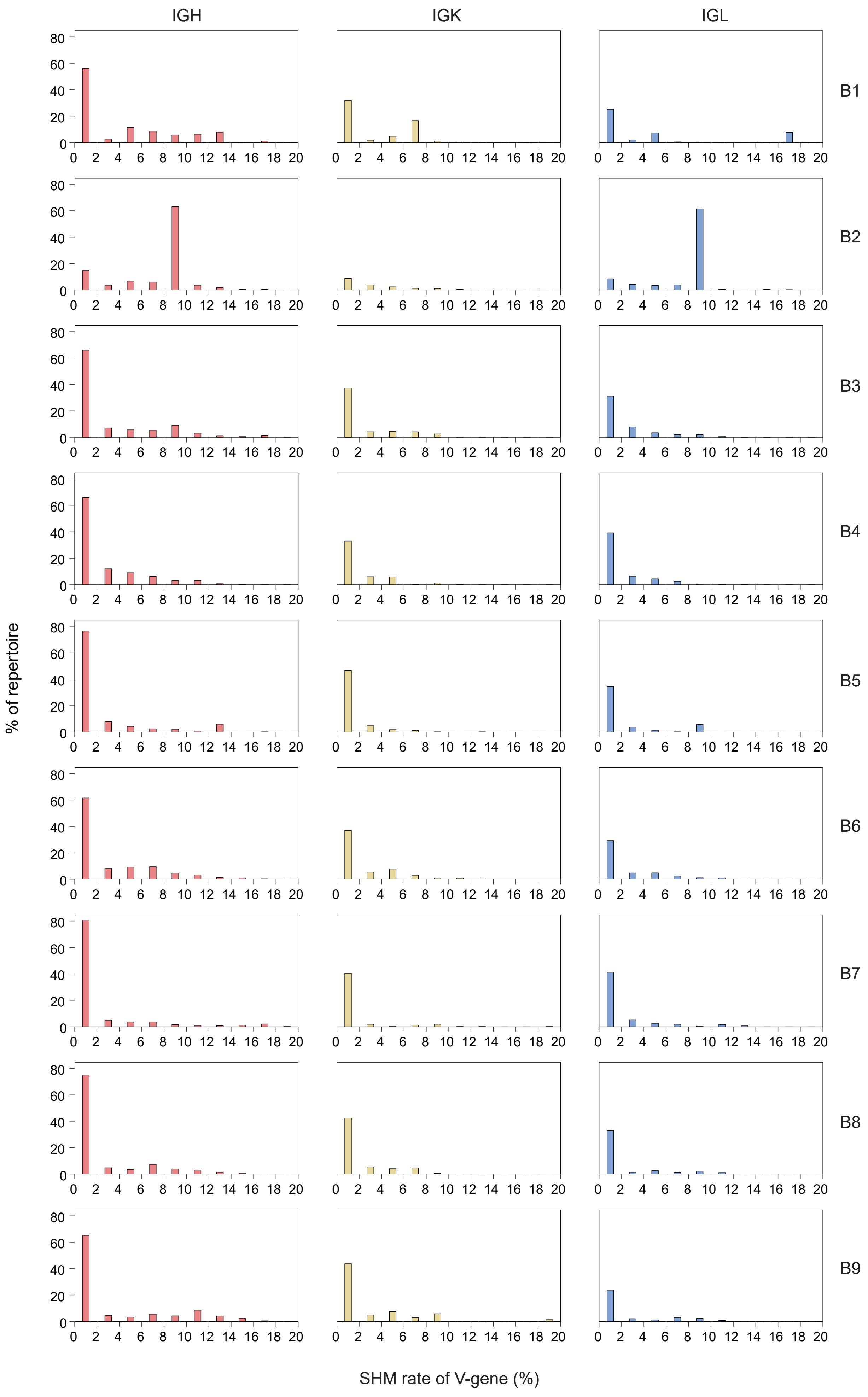
IGH
IGK
IGL
B1
B2
B3
B4
B5
B6
B7
B8
B9
% of repertoire
SHM rate of V-gene (%)
0 20 40 60 80
0 2 4 6 8 10 12 14 16 18 20

Figure S3

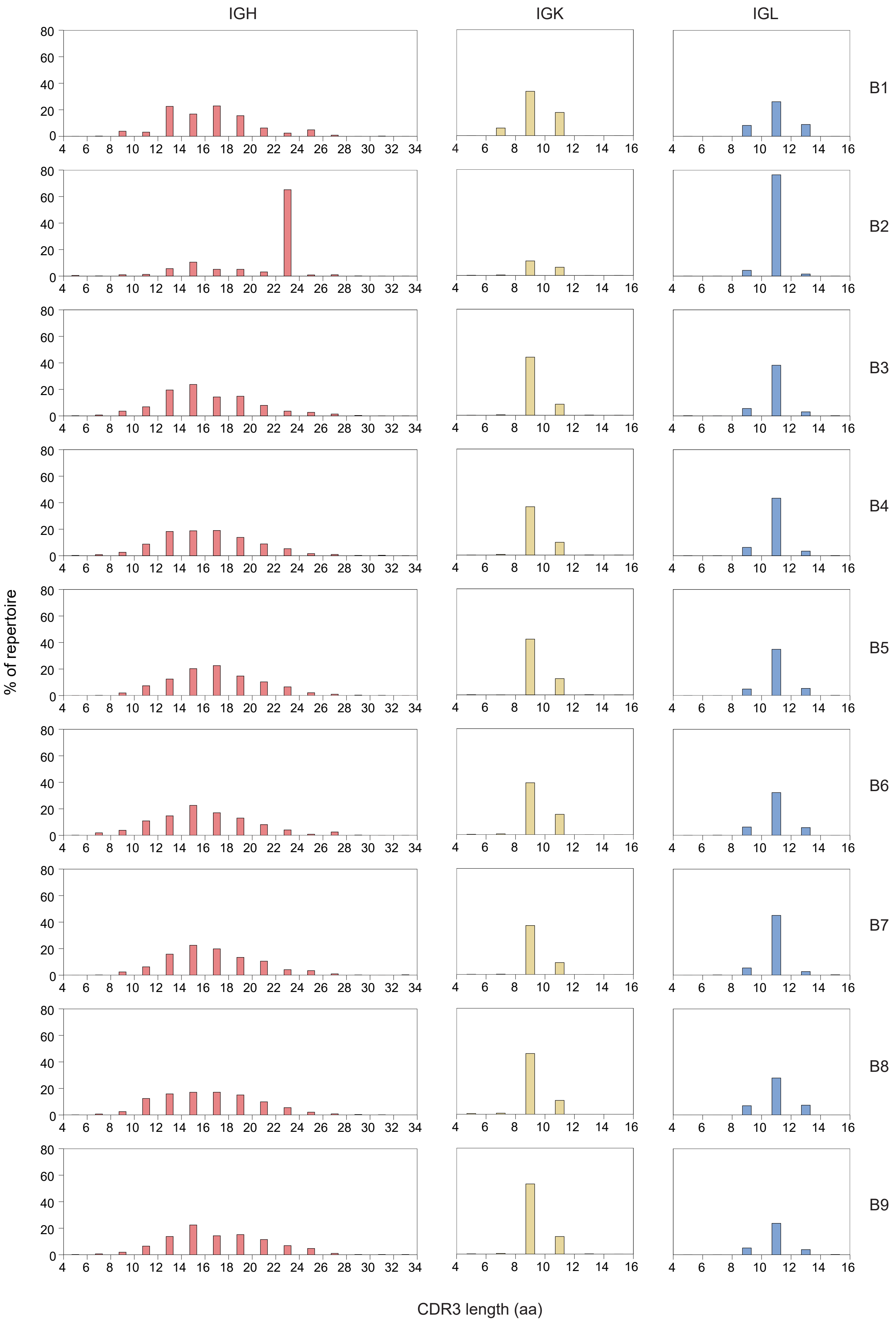

IGH
IGK
IGL
B1
B2
B3
B4
B5
B6
B7
B8
B9
% of repertoire
CDR3 length (aa)

Figure S4

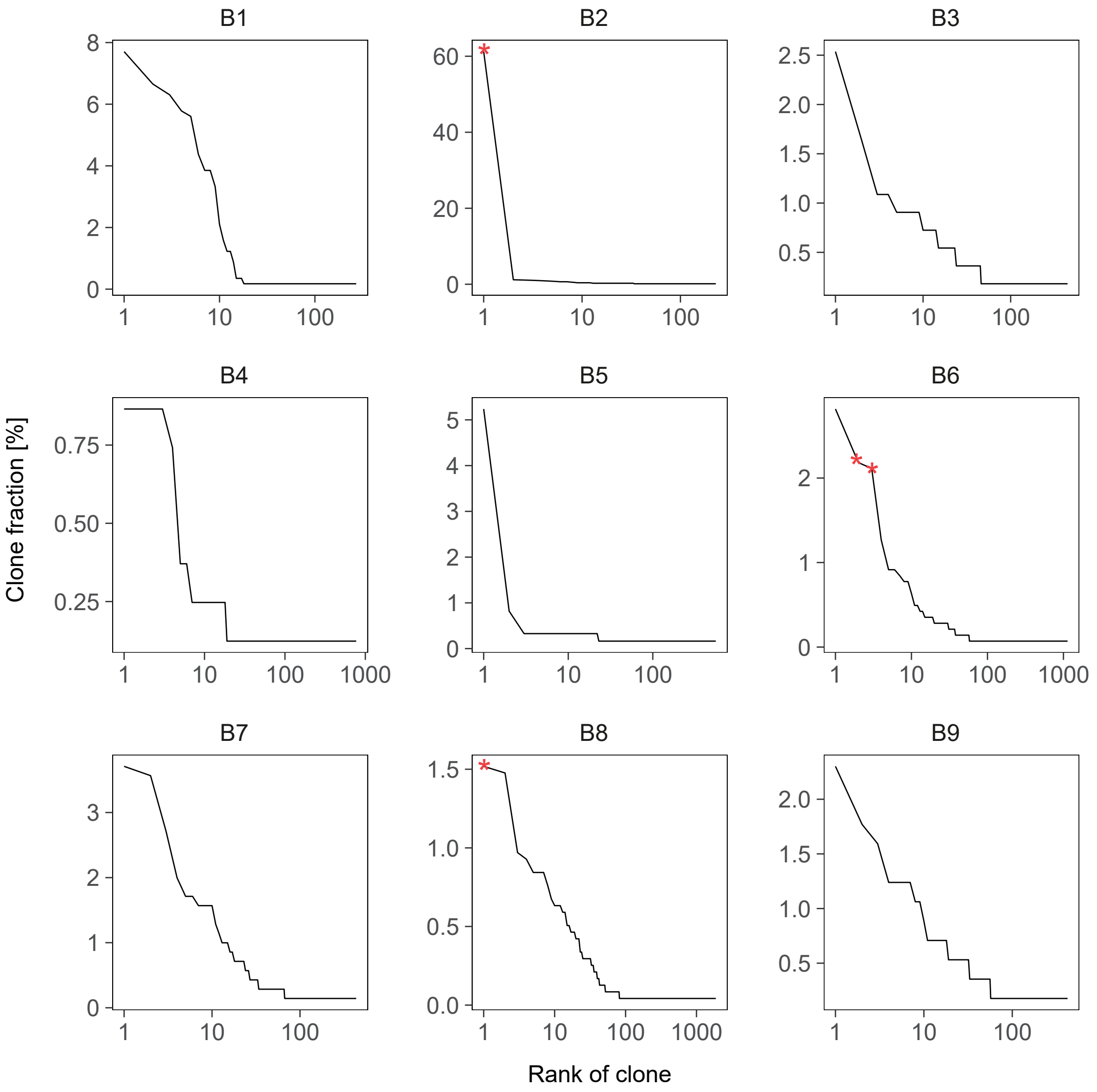
B1
B2
B3
B4
B5
B6
B7
B8
B9
Clone fraction [%]
Rank of clone
1
10
100
1000
*

Figure S5

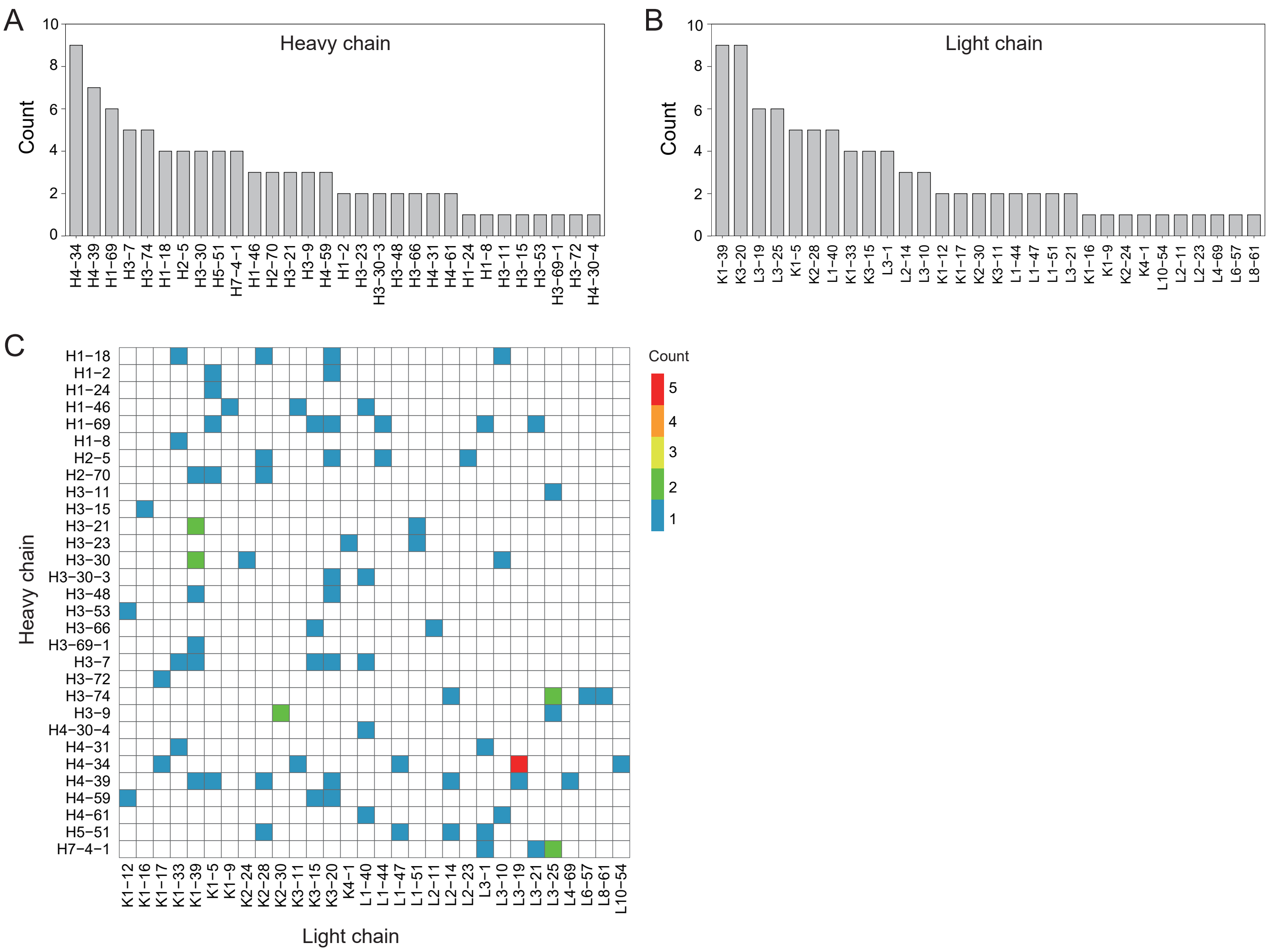
A
Heavy chain
Count
H4−34 H4−39 H1−69 H3−7 H3−74 H1−18 H2−5 H3−30 H5−51 H7−4−1 H1−46 H2−70 H3−21 H3−9 H4−59 H1−2 H3−23 H3−30−3 H3−48 H3−66 H4−31 H4−61 H1−24 H1−8 H3−11 H3−15 H3−53 H3−69−1 H3−72 H4−30−4
B
Light chain
Count
K1−39 K3−20 L3−19 L3−25 K1−5 K2−28 L1−40 K1−33 K3−15 L3−1 L2−14 L3−10 K1−12 K1−17 K2−30 K3−11 L1−44 L1−47 L1−51 L3−21 K1−16 K1−9 K2−24 K4−1 L10−54 L2−11 L2−23 L4−69 L6−57 L8−61
C
Heavy chain
Light chain
Count
5
4
3
2
1

Figure S6

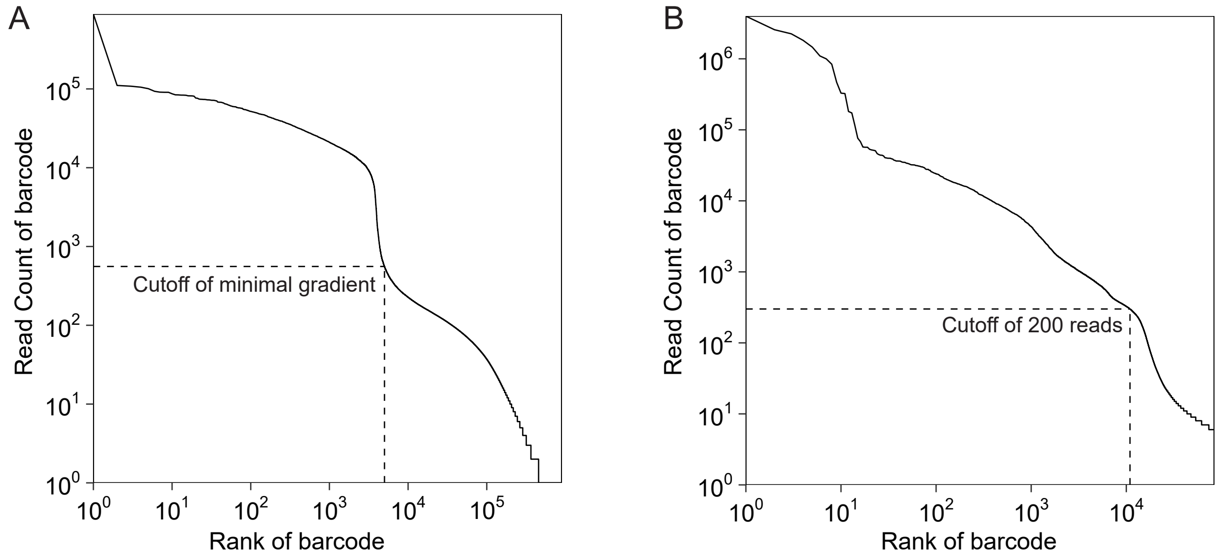
A
Read Count of barcode
Cutoff of minimal gradient
Rank of barcode
B
Read Count of barcode
Cutoff of 200 reads
Rank of barcode

Table S1

Patient ID	Gender	Age	Type
B1	Male	73	Severe
B2	Male	46	Severe
B3	Male	67	Severe
B4	Male	35	Mild
B5	Male	36	Mild
B6	Female	65	Severe
B7	Male	62	Severe
B8	Female	57	Severe
B9	Male	66	Severe

Table S2

CloneID	CloneSize	Heavy_Vgene	Heavy_Jgene	Light_Vgene	Light_Jgene	B1	B2	B5	B6	B8	B9
2	45	IGHV5-51	IGHJ4	IGLV1-47	IGLJ3	44	1	0	0	0	0
3	42	IGHV4-59	IGHJ4	IGKV3-15	IGKJ4	0	0	0	40	0	2
5	37	IGHV3-9	IGHJ4	IGKV2-30	IGKJ3	36	1	0	0	0	0
7	35	IGHV3-7	IGHJ4	IGKV3-20	IGKJ4	33	2	0	0	0	0
9	33	IGHV3-74	IGHJ4	IGLV6-57	IGLJ2,IGLJ3	32	1	0	0	0	0
10	33	IGHV4-34	IGHJ6	IGLV3-19	IGLJ3	0	0	0	30	0	3
11	33	IGHV3-30,IGHV3-30-5	IGHJ5	IGLV3-10	IGLJ2,IGLJ3	0	0	32	0	1	0
12	32	IGHV3-72	IGHJ4	IGKV1-17	IGKJ2	0	0	0	31	0	1
14	26	IGHV3-7	IGHJ4	IGKV1-33,IGKV1D-33	IGKJ5	25	1	0	0	0	0
16	23	IGHV3-74	IGHJ6	IGLV2-14	IGLJ2,IGLJ3	22	1	0	0	0	0
17	23	IGHV3-53	IGHJ5	IGKV1D-12	IGKJ4	22	1	0	0	0	0
23	20	IGHV2-70	IGHJ4	IGKV1-5	IGKJ2	19	1	0	0	0	0
24	19	IGHV7-4-1	IGHJ4	IGLV3-25	IGLJ2,IGLJ3	0	0	0	18	0	1
34	14	IGHV3-48	IGHJ4	IGKV1-39,IGKV1D-39	IGKJ2	0	0	0	13	0	1
36	14	IGHV4-59	IGHJ5	IGKV3-20	IGKJ1	0	0	0	13	0	1
38	13	IGHV4-59	IGHJ4	IGKV1-12,IGKV1D-12	IGKJ3	12	1	0	0	0	0
43	12	IGHV7-4-1	IGHJ4	IGLV3-25	IGLJ3	0	0	0	11	0	1
44	12	IGHV3-74	IGHJ6	IGLV8-61	IGLJ3	0	0	0	11	0	1
66	8	IGHV4-34	IGHJ4	IGLV2-11	IGLJ2,IGLJ3	7	1	0	0	0	0
68	8	IGHV1-2	IGHJ3	IGKV1-5	IGKJ1	0	0	0	7	0	1
96	6	IGHV3-49	IGHJ3	IGKV1-39,IGKV1D-39	IGKJ3	5	1	0	0	0	0
98	6	IGHV3-30-3	IGHJ4	IGKV1-5	IGKJ4	0	0	0	5	0	1
100	6	IGHV1-69	IGHJ6	IGKV1-27	IGKJ3	0	0	0	5	0	1
104	6	IGHV3-48	IGHJ1	IGKV1-27	IGKJ4	0	0	0	5	0	1
108	5	IGHV4-39	IGHJ4	IGKV1-39,IGKV1D-39	IGKJ4	0	0	0	4	0	1
110	5	IGHV1-8	IGHJ4	IGLV1-47	IGLJ3	0	0	0	3	0	2
121	5	IGHV3-74	IGHJ6	IGLV1-47	IGLJ3	0	0	0	4	0	1
122	5	IGHV3-43	IGHJ5	IGKV3-15	IGKJ4	0	0	0	4	0	1
124	5	IGHV3-43	IGHJ4	IGKV4-1	IGKJ4	0	0	0	4	0	1
126	5	IGHV3-43	IGHJ6	IGKV1-39,IGKV1D-39	IGKJ1	0	0	0	4	0	1
131	5	IGHV3-23,IGHV3-23D	IGHJ4	IGLV7-46	IGLJ3	0	0	0	4	0	1
135	4	IGHV3-13	IGHJ2	IGKV4-1	IGKJ5	0	0	0	3	0	1
153	4	IGHV4-59	IGHJ4	IGKV3-20	IGKJ1	0	0	0	3	0	1
157	4	IGHV1-3	IGHJ6	IGLV1-44	IGLJ6	0	0	0	3	0	1
176	3	IGHV3-30,IGHV3-30-3	IGHJ3	IGKV3-20	IGKJ1	0	0	0	2	0	1
207	3	IGHV4-4	IGHJ4	IGLV3-19	IGLJ2,IGLJ3	0	0	0	2	0	1
289	2	IGHV3-23,IGHV3-23D	IGHJ5	IGKV1-17	IGKJ4	0	0	0	1	0	1
340	2	IGHV3-11	IGHJ6	IGKV3-20	IGKJ1	0	0	0	1	0	1
369	2	IGHV3-30,IGHV3-33	IGHJ3	IGKV1-27	IGKJ1	1	1	0	0	0	0

Table S3

Antibody	Chain	Patient ID	Clone ID	Clone fraction (%)	Clone cell number	V gene	J gene	Isotype	V gene SHM (%)	CDR3 length (aa)
B2-C1	IGH	B2	1	61.21	464	IGHV4-34	IGHJ4	IGHG	9.215	23
B2-C1	IGL	B2	1	61.21	464	IGLV3-19	IGLJ2, IGLJ3	IGLC	8.042	11
B6-C2	IGH	B6	2	2.18	31	IGHV3-72	IGHJ4	IGHG	6.645	10
B6-C2	IGK	B6	2	2.18	31	IGKV1-17	IGKJ2	IGKC	4.895	9
B6-C3	IGH	B6	3	2.11	30	IGHV4-34	IGHJ6	IGHG	3.413	26
B6-C3	IGL	B6	3	2.11	30	IGLV3-19	IGLJ3	IGLC	5.190	11
B8-C1	IGH	B8	1	1.52	36	IGHV1-69	IGHJ4, IGHJ5	IGHG	7.143	11
B8-C1	IGK	B8	1	1.52	36	IGKV1-5	IGKJ4	IGKC	6.338	9

Figures for responding to reviewers

Figure R1

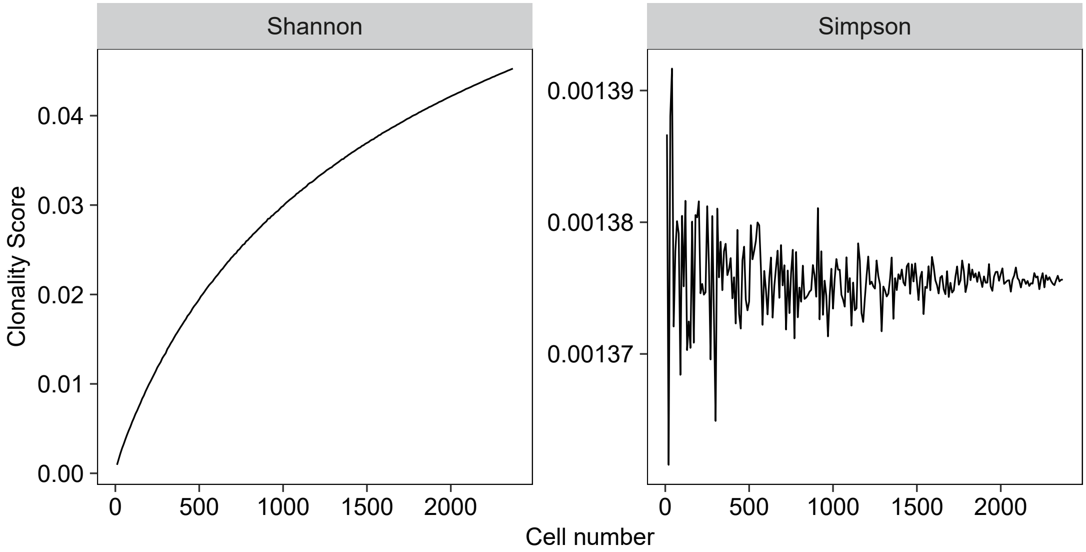

Figure R2

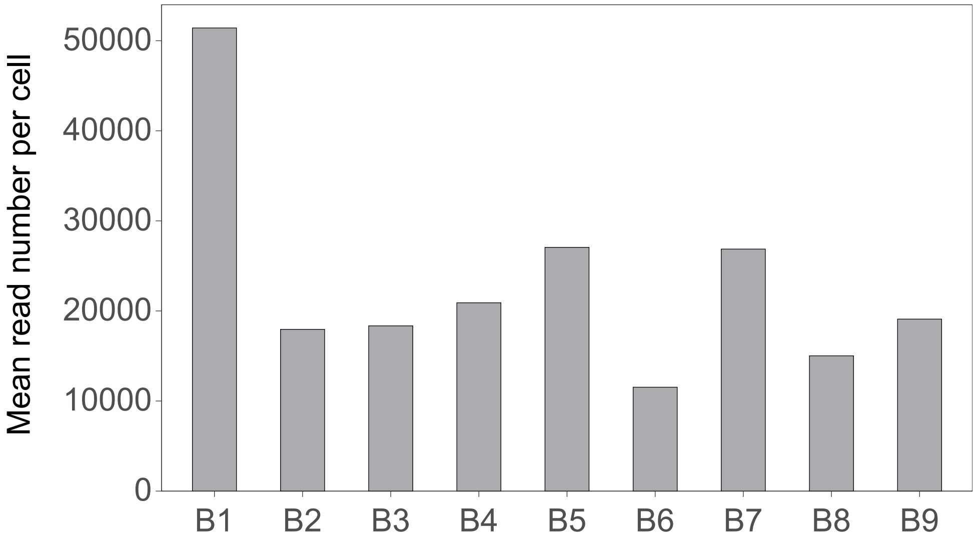

Supplement: giab050_GIGA-D-20-00341_Revision_1 [file giab050_giga-d-20-00341_revision_1.pdf]
