# Supplementary material for: SCIGA: Software for large-scale, single-cell immunoglobulin repertoire analysis
Source: Gigascience. 2021 Sep 28;10(9):giab050. doi: 10.1093/gigascience/giab050 (PMC8478610; doi:10.1093/gigascience/giab050)
Supplement: giab050_GIGA-D-20-00341_Revision_3 [file giab050_giga-d-20-00341_revision_3.pdf]

GigaScience

SCIGA: A software for large-scale, single-cell immunoglobulin repertories analysis

--Manuscript Draft--

**Manuscript Number:**	GIGA-D-20-00341R3
**Full Title:**	SCIGA: A software for large-scale, single-cell immunoglobulin repertories analysis
**Article Type:**	Technical Note
**Funding Information:**	Foundation for Distinguished Young Talents in Higher Education of Guangdong (82025022) — Dr. Zheng Zhang
**Abstract:**	Background<br>B cell immunoglobulin repertoires with paired heavy and light chain can be determined by the 10X single-cell V(D)J sequencing . Precise and quick analysis of 10X single-cell immunoglobulin repertoires remains a challenge due to the high diversity of immunoglobulin repertoires and a lack of specialized software that can analyze such diverse data.<br>Findings<br>In this study, specialized software for 10X single-cell immunoglobulin repertoire analysis was developed. SCIGA (Single-Cell Immunoglobulin Repertoire Analysis) is an easy-to-use pipeline that performs read trimming, immunoglobulin sequence assembly and annotation, heavy and light chain pairing, statistical analysis, visualization, and multiple sample integration analysis, which is all achieved by using a one-line command. Then SCIGA was used to profile the single-cell immunoglobulin repertoires of nine coronavirus disease 2019 (COVID-19) patients. Four neutralizing antibodies against severe acute respiratory syndrome coronavirus 2 (SARS-CoV-2) were identified from these repertoires.<br>Conclusions<br>SCIGA provides a complete and quick analysis for 10X single-cell V(D)J sequencing datasets. It can help researchers to interpret the B cell immunoglobulin repertoires with paired heavy and light chain.
**Corresponding Author:**	Zheng Zhang<br>Southern University of Science and Technology<br>Shenzhen, CHINA
**Corresponding Author Secondary Information:**	
**Corresponding Author's Institution:**	Southern University of Science and Technology
**Corresponding Author's Secondary Institution:**	
**First Author:**	Zheng Zhang
**First Author Secondary Information:**	
**Order of Authors:**	Zheng Zhang
	Haocheng Ye
	Lin Cheng
	Bin Ju
	Gang Xu
	Yang Liu
	Shuye Zhang
	Lifei Wang
**Order of Authors Secondary Information:**	
**Response to Reviewers:**	1) Since you submitted a PDF manuscript, can you make the following edits yourself

Powered by Editorial Manager® and ProduXion Manager® from Aries Systems Corporation

	then? In particular you will need to make some changes to the "Availability of supporting data" section. Please add a citation after "The data set(s) supporting the results of this article is(are) available in the [National Center for Biotechnology Information] repository (https://www.ncbi.nlm.nih.gov/), [PRJNA682839]." saying something like:<br>All supporting data and materials are available in the GigaScience GigaDB database [XX].<br>Then cite it as reference [XX] as:<br>Ye H; Cheng L; Ju B; Xu G; Liu Y; Zhang S; Wang L; Zhang Z. Supporting data for "SCIGA: A software for large-scale, single-cell immunoglobulin repertories analysis" GigaScience Database 2021. http://dx.doi.org/10.5524/100893.<br><br>Response:<br>Thank you for the comment. We added this citation in Line 273.<br><br><br>2) Also, please add the ORCIDs number for all authors (who have it) before "Abstract" like this:<br>ORCIDs:<br>Yujing Suo, 0000-0002-1080-5094;<br>Peng Sun, 0000-0001-5746-9684.<br><br>Response:<br>Thank you for the comment. We added the ORCIDs in Line 26 ~ 32.<br><br><br>3) And add the office phone number of the corresponding author(s) if available.<br><br>Response:<br>Thank you for the comment. We added the office phone number of the corresponding authors in Line 20 ~21 and Line 23 ~ 24.
**Additional Information:**	
**Question**	**Response**
Are you submitting this manuscript to a special series or article collection?	No
**Experimental design and statistics**<br><br>Full details of the experimental design and statistical methods used should be given in the Methods section, as detailed in our Minimum Standards Reporting Checklist. Information essential to interpreting the data presented should be made available in the figure legends.<br><br>Have you included all the information requested in your manuscript?	Yes
**Resources**<br><br>A description of all resources used, including antibodies, cell lines, animals and software tools, with enough	Yes

Powered by Editorial Manager® and ProduXion Manager® from Aries Systems Corporation

information to allow them to be uniquely identified, should be included in the Methods section. Authors are strongly encouraged to cite Research Resource Identifiers (RRIDs) for antibodies, model organisms and tools, where possible.<br><br>Have you included the information requested as detailed in our Minimum Standards Reporting Checklist?	
**Availability of data and materials**<br><br>All datasets and code on which the conclusions of the paper rely must be either included in your submission or deposited in publicly available repositories (where available and ethically appropriate), referencing such data using a unique identifier in the references and in the “Availability of Data and Materials” section of your manuscript.<br><br>Have you have met the above requirement as detailed in our Minimum Standards Reporting Checklist?	Yes

Manuscript
Click here to access/download;Manuscript;maintext.docx

SCIGA: A software for large-scale, single-cell immunoglobulin repertoires analysis

Haocheng Ye[1,3#], Lin Cheng[1#], Bin Ju[1], Gang Xu[1], Yang Liu[1], Shuye Zhang[4], Lifei Wang[2*] , Zheng Zhang[1*]

[1]Institute for Hepatology, National Clinical Research Center for Infectious Disease, Shenzhen Third People's Hospital, The Second Affiliated Hospital, School of Medicine, Southern University of Science and Technology, Shenzhen, Guangdong 518112, China.

[2]Department of Radiology, National Clinical Research Center for Infectious Disease, Shenzhen Third People's Hospital, The Second Affiliated Hospital, School of Medicine, Southern University of Science and Technology, Shenzhen, Guangdong 518112, China.

[3]CAS Key Laboratory of Pathogenic Microbiology and Immunology, Institute of Microbiology, Chinese Academy of Sciences (CAS), Beijing, 100101, China

[4]Shanghai Public Health Clinical Center, Fudan University, Shanghai, 201508, China.

[#]These authors contributed equally.

***Correspondence:**

Zheng Zhang. Institute of Hepatology, Shenzhen 3rd People's Hospital, Shenzhen, Guangdong Province 518100, China; Email: zhangzheng1975@aliyun.com. Phone: 86-755-81238983.

Lifei Wang. Department of Radiology, Shenzhen 3rd People's Hospital, Shenzhen,

Guangdong Province 518100, China; Email: wanglf007n@163.com. Phone: 86-755-61238936.

ORCIDs:

Haocheng Ye, 0000-0002-4055-7175;

Lin Cheng, 0000-0001-8066-527X;

Bin Ju, 0000-0003-0768-6327;

Shuye Zhang, 0000-0002-4749-6790;

Lifei Wang, 0000-0001-8193-4910;

Zheng Zhang, 0000-0002-3544-1389;

Abstract

Background

B cell immunoglobulin repertoires with paired heavy and light chain can be determined by the 10X single-cell V(D)J sequencing. Precise and quick analysis of 10X single-cell immunoglobulin repertoires remains a challenge due to the high diversity of immunoglobulin repertoires and a lack of specialized software that can analyze such diverse data.

Findings

In this study, specialized software for 10X single-cell immunoglobulin repertoire analysis was developed. SCIGA (Single-Cell Immunoglobulin Repertoire Analysis) is an easy-to-use pipeline that performs read trimming,

immunoglobulin sequence assembly and annotation, heavy and light chain pairing, statistical analysis, visualization, and multiple sample integration analysis, which is all achieved by using a one-line command. Then SCIGA was used to profile the single-cell immunoglobulin repertoires of nine coronavirus disease 2019 (COVID-19) patients. Four neutralizing antibodies against severe acute respiratory syndrome coronavirus 2 (SARS-CoV-2) were identified from these repertoires.

Conclusions

SCIGA provides a complete and quick analysis for 10X single-cell V(D)J sequencing datasets. It can help researchers to interpret the B cell immunoglobulin repertoires with paired heavy and light chain.

Key words: Software; Single-cell; Immunoglobulin repertoires; COVID-19; Antibody

Background

The diversity of B cell immunoglobulin is an important characteristic of the adaptive immune system. It is developed through the rearrangement of variable V, (diversity D) and the joining of J gene segments, which is referred to as V(D)J, the pairing of heavy and light chains, and the somatic hypermutation (SHM) [1]. Exposure to infections and environmental factors shapes the repertoire of B cell immunoglobulins [2-4], and leads to clonal expansion of immune cells, allowing them to change into different types of cells to respond to a specific antigen.

Understanding these immunoglobulin repertoires can help researchers to discover antibodies, monitor vaccination responses and infer B cell trafficking patterns [5, 6].

10X single-cell V(D)J sequencing is a powerful tool for investigating paired heavy and light chain repertoires of B cell immunoglobulins [7]. It has been used in the identification of neutralizing antibodies against severe acute respiratory syndrome coronavirus 2 (SARS-CoV-2) [8], the virus that causes coronavirus disease 2019 (COVID-19) [9]. However, accurately analyzing 10X single-cell immunoglobulin repertoires remains a challenge due to the high diversity of immunoglobulin repertoires and the lack of specialized software that can analyze such diverse data.

Here, we developed SCIGA (Single-Cell Immunoglobulin Repertoire Analysis), a software for quickly analyzing the data of 10X single-cell immunoglobulin repertoires. SCIGA performs read trimming, immunoglobulin sequence assembly and annotation, heavy and light chain pairing by a one-line command. It also computes the statistics of repertoires, including gene usage frequency, SHM rate, length of complementarity determining region 3 (CDR3), and clonality, and further implements visualization. We profiled the immunoglobulin repertoires of peripheral blood mononuclear cells (PBMCs) from nine COVID-19 patients using SCIGA. Finally, we identified four neutralizing antibodies against SARS-CoV-2 from these repertoires.

Methods

SCIGA is a software for the analysis of 10X single-cell immunoglobulin repertoires. It integrates several tools and algorithms into a single workflow. The input data can be raw reads or the output of Cell Ranger (RRID:SCR_017344) [10]. The details of the SCIGA algorithm can be found in the Supplementary Methods and Materials. Briefly, the workflow, which is summarized in Fig. 1, is as follows: 1) Quality control of reads. Trim the reads of low-quality using Trimmomatic (RRID:SCR_011848)[11]; 2) Call cell. The 10X system generates a large amount of Gel Beads-in-Emulsion (GEMs) that contain no cell. We need to identify the cell-containing GEMs before further analysis. SCIGA considers the GEMs containing cell(s) when the read number of the GEMs is over a threshold (see the Supplementary Methods and Materials); 3) Immunoglobulin sequence assembly. The immunoglobulin sequences for each cell were assembled using SSAKE (RRID:SCR_010753) [12], which is a reliable de novo assembler for short reads; 4) Gene call. To detect the usage of the V(D)J gene and C gene (isotype), SCIGA aligns the assembled immunoglobulin sequences against the V-, D-, and J- gene reference database using IgBLAST (RRID:SCR_002873)[13] and against C-gene reference database using BLAST (RRID:SCR_004870)[14]. The V(D)JC reference databases for humans, mice and rats were downloaded from the international immunogenetics information system (IMGT) [15] and embedded in the SCIGA software; 5) Quality control of the immunoglobulin sequence. Only the immunoglobulins that are complete, in

the correct reading frame and have no stop codon are retained; 6) Quality control of the cells. After immunoglobulin sequence assembly and filtering, some cells have multiple heavy or light chains, whereas some cells have only one chain. SCIGA reports the heavy and light chain with the highest number of unique molecular identifiers (UMIs) for each cell. A certainty score is calculated for each reported chain (see the Supplementary Methods and Materials). The chains with a certainty score less than a given threshold are discarded. Next, the cells without paired heavy and light chains are filtered out; 7) Clonal lineage grouping. Clonal lineage is defined as the cells that have identical V_H, J_H, V_L and J_L genes, identical H-CDR3 length, and over a given similarity threshold of H-CDR3 nucleotide sequences; 8) Statistical analysis and visualization. SCIGA calculates a list of statistics, including gene usage frequency, SHM rate, CDR3 length, Simpson index, Shannon entropy, and others. SCIGA subsequently generates figures to show the features of the repertoires. 9) Multiple sample integration analysis. After analyzing each sample, SCIGA consolidates all of the outputs into one. It identifies the shared immunoglobulins that are potential public antibodies suitable for use against a specific pathogen. Shared immunoglobulins are defined as immunoglobulins from different samples that can be clustered into the same clonal lineage. Clustering is performed as described in step 7 with cells of all samples.

Findings

Comparing SCIGA to existing software

At the time of this study, Cell Ranger is the only existing software for processing raw data generated by 10X single-cell V(D)J sequencing. A test dataset was therefore built to compare SCIGA to Cell Ranger. The PBMCs from nine COVID-19 patients (B1 to B9) were collected and 10X single-cell V(D)J sequencing was performed (Fig. 2A and Table S1). The raw data were analyzed using SCIGA and Cell Ranger (v3.1.0) with the default parameters. The comparison mainly focused on the following aspects: 1) Cell quality control. For Cell Ranger, the final results still included low-quality cells that either had multiple heavy or light chains, or only one chain. In our test dataset, the percentage of the low-quality cells reached an average of 29% (16.8% to 47.6% per sample, Fig. 2B). SCIGA implements the cell quality control process (step 6 described in Methods) and only outputs the high-quality cells. The cell count was generally less in the output of SCIGA compared to Cell Ranger due to the strict quality control process (Fig. 2C); 2) Detecting B cell clonal lineage. Cell Ranger clusters B cells into a clonal lineage when cells have identical nucleotide sequences of CDR3. However, it will break up the clonotypes that are clonally related in fact when the SHM falls within the CDR3 region. SCIGA uses a popular clonal grouping method (step 7 of Methods), which considers the SHM and allows mismatch in the CDR3 region. Therefore, SCIGA could detect a larger clonal lineage than Cell Ranger (Fig. 2D); 3) Output information. The output of Cell Ranger is quite limited and some important information, such

as SHM rate, is not included. SCIGA outputs the necessary information, including gene frequency, clone frequency, clonality, SHM rate, CDR3 length, the immunoglobulin variable region sequence, and others (Fig. 2E). Moreover, SCIGA is able to implement visualization to display the features of the repertoires; 4) Detecting shared immunoglobulin. This is a specific function in SCIGA and it could detect the shared immunoglobulin across samples.

Determining the features of immunoglobulin repertoires of COVID-19 using SCIGA

We performed a trial study to show the usage and performance of SCIGA. The 10X V(D)J sequencing data of the nine COVID-19 patients were analysis and features of the immunoglobulin repertoires were determined. A total of 8,358 B cells were detected (571-2,371 cells per sample, Fig. 2C). We focused on the genes used in at least 1% of B cells for the V-gene usage (Fig. 3A and Fig. S1). The top three gene families were IGHV4-34 (12.51%), IGHV3-30 (7.95%), and IGHV3-23 (6.30%) for the heavy chain, and IGLV3-19 (9.46%), IGKV1-39 (8.26%), and IGKV3-20 (7.90%) for the light chain. IGHV4-34 and IGLV3-19 had elevated usage frequency in the repertoire of patient B2, and reached 63.98% and 64.39%, respectively. IGHM had the highest usage in the repertoires of most patients, except for patient B2, who showed the highest usage of IGHG1 (Fig. 3B). The SHM levels were low in the repertoires of most patients (<2%, Fig. 3C and Fig. S2). However, patient B2 showed a high SHM level in the IGH chain (7.48%) and IGL chain (7.13%). Moreover, patient B2 had

an elevated CDR3 length for its immunoglobulin repertoire (Fig. 3D and Fig. S3).

Clonal lineage analysis using SCIGA

Clonal lineages were grouped using SCIGA with the default parameter. We used the Simpson index and Shannon entropy to determine the clonality of the immunoglobulin repertoires (Fig. 4A and 4B). Both indices showed that the repertoires of patient B2 experienced clonal expansion. The top 10 largest clones of each patient were reviewed (Fig. 4C and Fig. S4). The frequency of the largest clone for most patients was below 8%. However, the largest clone in patient B2 reached a frequency of 61.21%. This strongly expanded clone used the IGHV4-34 and IGLV3-19 genes, with an 8.82% mean SHM rate and 23-amino acid H-CDR3 length. We determined the most used V-genes in the top 10 largest clones of all patients. It was observed that IGHV4-34 (nine clones) was the most used gene in the heavy chain, IGKV1-39 (nine clones) and IGKV3-20 (nine clones) were the most used genes in the light chain, and IGHV4-34: IGLV3-19 (five clones) was the most used gene pair (Fig. S5A and S5B and S5C). Next, the shared immunoglobulin sequences across patients were determined using SCIGA. There were 12 immunoglobulins shared between patient B1 and B2, 1 immunoglobulin was shared between patient B5 and B8, and 26 immunoglobulins were shared between patient B6 and B9 (Fig. 4D and Table S2).

Identification of neutralizing monoclonal antibodies

It was hypothesized that IgGs with higher clonal expansion may be SARS-CoV-2-specific antibodies in the COVID-19 patients. Thus, monoclonal antibodies (mAbs) were screened for the following criteria: IgG antibodies in the clone with fraction ≥ 1% and cell number ≥ 20 (see the Supplementary Methods and Materials). Four mAbs met the criteria and were expressed: B2-C1, B6-C2, B6-C3, and B8-C1 (Fig. 4C and Table S3). Remarkably, enzyme-linked immunosorbent assay (ELISA) revealed that all four mAbs were SARS-CoV-2 RBD (receptor binding domain) specific antibodies, which bound to the extracellular domain (ECD), the S1 subunit, and the RBD of the SARS-CoV-2 spike (Fig. 5A). They did not bind to the N-terminal domain (NTD) and the S2 subunit. The monoclonal antibodies could neutralize SARS-CoV-2 by blocking the attachment of RBD to the receptor (angiotensin-converting enzyme 2, ACE2) on host cells. B2-C1 and B6-C3 exhibited potent neutralizing activity [half-maximal inhibitory concentrations (IC_{50}) = 0.75 μg/ml and 0.32 μg/ml, respectively] against SARS-CoV-2 pseudovirus, whereas B8-C1 (1.47 μg/ml) and B6-C2 (14.89 μg/ml) were moderate and weak neutralizing antibodies (Fig. 5B). Similar results were found for the neutralization of the four mAbs against SARS-CoV-2 live virus (Fig. 5C).

Discussion

In this study, we developed the SCIGA software for 10X single-cell immunoglobulin repertoire analysis. SCIGA is an easy-to-use software and allows researchers to quickly perform advanced analysis on 10X V(D)J

sequencing datasets. Cell Ranger has previously been used for 10X single-cell immunoglobulin repertoire analysis. However, this software includes low-quality cells in the output and disregards the effect of SHM when defining clonal lineage. In addition, some important information about repertoires, including the level of SHM for example, is not included in the output of Cell Ranger. SCIGA performs the quality control process for cells and defines the clonal lineage including the effect of SHM. Larger clones can be detected by using SCIGA. Moreover, SCIGA generates the needed statistical outputs and implements visualization. It is therefore a more efficacious tool for researchers.

In this study, SCIGA was used to analyze the single-cell immunoglobulin repertoires of the PBMCs of COVID-19 patients. Large-scale clone expansion was not observed in most patients. In patient B2, however, B cells expanded, which was indicated by a large size of clonal lineage with the IgG isotype. This indicates that patient B2 likely generated neutralizing antibodies against SARS-CoV-2.

Finally, we tried to identify the SARS-CoV-2-responding antibodies. In previous work, immunoglobulins with an SHM rate lower than 2% were excluded in screens for neutralizing antibodies [8]. However, some studies have shown that several potent neutralizing antibodies against SARS-CoV-2 have low SHM rates [16-18]. Therefore, we included the antibodies with low SHM levels in our work. Four neutralizing antibodies with different potency were identified using our criteria. This demonstrates that SCIGA is useful for 10X single-cell

immunoglobulin repertoire analysis.

List of abbreviations

ACE2: angiotensin-converting enzyme 2

CDR3: complementarity determining region 3

COVID-19: coronavirus disease 2019

ECD: extracellular domain

GEMs: Gel Beads-in-emulsion

IC50: half-maximal inhibitory concentration

Ig: immunoglobulin

mAbs: monoclonal antibodies

NTD: N-terminal domain

PBMCs: peripheral blood mononuclear cells

RBD: receptor binding domain

SARS-CoV-2: severe acute respiratory syndrome coronavirus 2

SHM: somatic hypermutation

UMI: unique molecular identifier

Availability of supporting source code and requirements

Project name: SCIGA (RRID: SCR_021002, Biotools ID: sciga)

Project home page: https://github.com/sciensic/SCIGA

Operating system(s): Linux

Programming language: Perl

Other requirements: IgBlast 1.15.0 or higher, Blast 2.9.0 or higher, R (optional), ggplot2 (optional)

License: GNU GPL-3.0 License

Data Availability

The data sets supporting the results of this article are available in the NCBI repository (https://www.ncbi.nlm.nih.gov/), [PRJNA682839]. All additional supporting data and materials are available in the *GigaScience* GigaDB database[19].

Ethics, consent and permissions

This study was conducted according to the ethical principles of the Declaration of Helsinki. Ethical approval was obtained from the Research Ethics Committee of Shenzhen Third People's Hospital (2020-207). All participants provided written informed consent for sample collection and subsequent analyses.

Authors' contributions

Z.Z. and L.W. designed this study and wrote the manuscript. H.Y. performed this study and wrote the manuscript. L.C. performed the antibody neutralization test and wrote the manuscript. B.J. performed the ELISA test. G.X. performed the 10X single-cell V(D)J sequencing. Y.L. revised the manuscript. S.Z.

contributed to the discussion.

Competing interests

The authors declare that they have no competing interests.

Funding

This study was supported by the National Science Fund for Distinguished Young Scholars (82025022), the Sanming Project for Medicine of Shenzhen (SZSM201612053), the National Key Plan for Scientific Research and Development of China (2020YFC0848800, 2020YFC0844200), the National Science and Technology Major Project of the Infectious Diseases (2018ZX10301404 to ZZ and SZ), the Science and Technology Innovation Committee of Shenzhen Municipality (202002073000002, 2020A1111350032, JCYJ20190809115617365), the National Natural Science Foundation of China (82002140) and the Natural Science Foundation of Guangdong Province of China (2019A1515011197).

Acknowledgements

We thank LetPub (www.letpub.com) for its linguistic assistance during the preparation of this manuscript.

References

1. V, G., et al., *- Bioinformatic and Statistical Analysis of Adaptive Immune Repertoires.* -

Trends Immunol. 2015 Nov;36(11):738-749. doi: 10.1016/j.it.2015.09.006. Epub 2015, (- 1471-4981 (Electronic)): p. - 738-749.

2. FA, T., et al., - *Biased IGH VDJ gene repertoire and clonal expansions in B cells of chronically.* - Blood. 2018 Feb 1;131(5):546-557. doi: 10.1182/blood-2017-09-805762. Epub 2017 Dec, (- 1528-0020 (Electronic)): p. - 546-557.

3. SCA, N., et al., - *Shaping of infant B cell receptor repertoires by environmental factors and.* - Sci Transl Med. 2019 Feb 27;11(481):eaat2004. doi: 10.1126/scitranslmed.aat2004., (- 1946-6242 (Electronic)): p. T - ppublish.

4. A, N., et al., - *Fierce Selection and Interference in B-Cell Repertoire Response to Chronic HIV-1.* - Mol Biol Evol. 2019 Oct 1;36(10):2184-2194. doi: 10.1093/molbev/msz143., (- 1537-1719 (Electronic)): p. - 2184-2194.

5. H, R., - *Immunosequencing: applications of immune repertoire deep sequencing.* - Curr Opin Immunol. 2013 Oct;25(5):646-52. doi: 10.1016/j.coi.2013.09.017. Epub 2013, (- 1879-0372 (Electronic)): p. - 646-52.

6. JN, S., et al., - *B cells populating the multiple sclerosis brain mature in the draining cervical.* - Sci Transl Med. 2014 Aug 6;6(248):248ra107. doi: 10.1126/scitranslmed.3008879., (- 1946-6242 (Electronic)): p. - 248ra107.

7. LD, G., et al., - *Massively parallel single-cell B-cell receptor sequencing enables rapid discovery of.* - Commun Biol. 2019 Aug 9;2:304. doi: 10.1038/s42003-019-0551-y. eCollection 2019., (- 2399-3642 (Electronic)): p. - 304.

8. Y, C., et al., - *Potent Neutralizing Antibodies against SARS-CoV-2 Identified by High-Throughput.* - Cell. 2020 Jul 9;182(1):73-84.e16. doi: 10.1016/j.cell.2020.05.025. Epub

2020 May, (- 1097-4172 (Electronic)): p. - 73-84.e16.

9. D, W., et al., - *The SARS-CoV-2 outbreak: What we know.* - Int J Infect Dis. 2020 May;94:44-48. doi: 10.1016/j.ijid.2020.03.004. Epub 2020 Mar, (- 1878-3511 (Electronic)): p. - 44-48.

10. Cellranger: https://support.10xgenomics.com/single-cell-gene-expression/software/downloads/latest? Accessed 1st May 2021

11. AM, B., L. M, and U. B, - *Trimmomatic: a flexible trimmer for Illumina sequence data.* - Bioinformatics. 2014 Aug 1;30(15):2114-20. doi: 10.1093/bioinformatics/btu170. Epub, (- 1367-4811 (Electronic)): p. - 2114-20.

12. RL, W., et al., - *Assembling millions of short DNA sequences using SSAKE.* - Bioinformatics. 2007 Feb 15;23(4):500-1. doi: 10.1093/bioinformatics/btl629. Epub, (- 1367-4811 (Electronic)): p. - 500-1.

13. J, Y., et al., - *IgBLAST: an immunoglobulin variable domain sequence analysis tool.* - Nucleic Acids Res. 2013 Jul;41(Web Server issue):W34-40. doi: 10.1093/nar/gkt382., (- 1362-4962 (Electronic)): p. - W34-40.

14. C, C., et al., - *BLAST+: architecture and applications.* - BMC Bioinformatics. 2009 Dec 15;10:421. doi: 10.1186/1471-2105-10-421., (- 1471-2105 (Electronic)): p. - 421.

15. MP, L., et al., - *IMGT®, the international ImMunoGeneTics information system® 25 years on.* - Nucleic Acids Res. 2015 Jan;43(Database issue):D413-22. doi: 10.1093/nar/gku1056., (- 1362-4962 (Electronic)): p. - D413-22.

16. C, K., et al., - *Longitudinal Isolation of Potent Near-Germline SARS-CoV-2-Neutralizing Antibodies.* - Cell. 2020 Aug 20;182(4):843-854.e12. doi: 10.1016/j.cell.2020.06.044.

Epub 2020 Jul, (- 1097-4172 (Electronic)): p. - 843-854.e12.

17. B, J., et al., - *Human neutralizing antibodies elicited by SARS-CoV-2 infection.* - Nature. 2020 Aug;584(7819):115-119. doi: 10.1038/s41586-020-2380-z. Epub 2020 May, (- 1476-4687 (Electronic)): p. - 115-119.

18. TF, R., et al., - *Isolation of potent SARS-CoV-2 neutralizing antibodies and protection from disease.* - Science. 2020 Aug 21;369(6506):956-963. doi: 10.1126/science.abc7520. Epub 2020 Jun, (- 1095-9203 (Electronic)): p. - 956-963.

19. H, Y., et al., *Supporting data for "SCIGA: A software for large-scale, single-cell immunoglobulin repertories analysis" GigaScience Database 2021.* http://dx.doi.org/10.5524/100893.

20. I, L., et al., - *BraCeR: B-cell-receptor reconstruction and clonality inference from single-cell.* - Nat Methods. 2018 Aug;15(8):563-565. doi: 10.1038/s41592-018-0082-3., (- 1548-7105 (Electronic)): p. - 563-565.

21. N, C. and W. DR, - *Analyzing Immunoglobulin Repertoires.* - Front Immunol. 2018 Mar 14;9:462. doi: 10.3389/fimmu.2018.00462. eCollection 2018., (- 1664-3224 (Print)): p. - 462.

22. L, Z., et al., - *Lineage tracking reveals dynamic relationships of T cells in colorectal cancer.* - Nature. 2018 Dec;564(7735):268-272. doi: 10.1038/s41586-018-0694-x. Epub 2018 Oct, (- 1476-4687 (Electronic)): p. - 268-272.

Figure legend

Fig. 1 The workflow of SCIGA. The workflow includes quality control of reads,

call cell, immunoglobulin (Ig) sequence assembly, V(D)JC gene call, quality control of Ig sequence, cell quality control, group clonal lineage, statistical analysis and visualization, and multiple sample integration analysis.

Fig. 2 Comparison of SCIGA and Cell Ranger. (A) Flowchart of the experiment. **(B)** The percentage of low-quality cells in the output of Cell Ranger. Single denotes the cells containing single chain. Multiple denotes the cells containing multiple heavy or light chains. **(C)** The count of B cells in the output of SCIGA and Cell Ranger. **(D)** The fraction of the top 10 largest clone analyzed using SCIGA and Cell Ranger. **(E)** The output information of the SCIGA and Cell Ranger.

Fig. 3 The features of the single-cell immunoglobulin repertoires of nine COVID-19 patients. (A) The average usage frequency of V-genes in the repertoires. Only show the genes with frequency > 1%. **(B)** The usage frequency of isotypes in the repertoire of each patient. Colors denote the isotypes. **(C)** The mean SHM rate of V-genes **(D)** The mean CDR3 length in the repertoire of each patient, with IGH shown in red, IGK in yellow and IGL in blue. The error bars represent the standard error.

Fig. 4 The B cell clonal expansion of nine COVID-19 patients. (A) The Simpson index **(B)** The Shannon entropy denote the clonality of the repertoire

of each patient. **(C)** The top 10 largest clones in the repertoire of each patient. The x-axis captures the clone ID and used V-genes. The initial of the gene names denote the chain, with H is IGH, K is IGK, and L is IGL. * denotes the screened antibody candidate. **(D)** Number of immunoglobulins shared between patients. Blank means zero.

Fig. 5 Characteristics of the spike specific monoclonal antibodies. (A) The binding profile of selected monoclonal antibodies to the extracellular domain and subdomains of the SARS-CoV-2 spike by ELISA. HIV-1-GP140 is the negative control. **(B-C)** Neutralization activity of selected monoclonal antibodies against the pseudovirus (**B**) and live SARS-CoV-2 (**C**). The dashed line indicates a 50% reduction in viral infectivity. Human IgG1 is the negative control. Results presented here are the representative of two independent experiments.

Fig. S1 Frequency of V-genes in the repertoire of each patient. Only the genes with a frequency > 1% are shown.

Fig. S2 Distribution of the SHM rate in the repertoire of each patient. Color denotes the chain, with IGH shown in red, IGK in yellow and IGL in blue.

Fig. S3 Distribution of the CDR3 length in the repertoire of each patient. Color denotes the chain, with IGH shown in red, IGK in yellow and IGL in blue.

Fig. S4 Fraction of all clones in the repertoire of each patient. The x-axis captures the clone rank and y-axis captures the clone fraction. * denotes the selected antibody candidate.

Fig. S5 Number of the used V-genes of the top 10 largest clones of all patients. (A) The count of the used V-gene for the heavy chain. **(B)** The count of the used V-gene for the light chain. **(C)** The count of the used V-gene pair. The initial of the gene names denote the chain, with H is IGH, K is IGK, and L is IGL.

Fig. S6 Two different examples show how to chose the threshold. The example having **(A)** and not having **(B)** large difference in the read counts between cells-containing GEMs and background.

Table. S1 Information of the nine COVID-19 patients.

Table. S2 Information of the shared immunoglobulins. The columns 7-11 denote the number of shared immunoglobulin sequences.

Table. S3 Information of the four antibody candidates.

Supplementary Methods and Materials

Note 1. Algorithm of SCIGA

Note 2. Sample processing and sequencing

Note 3. Data analysis

Note 4. Identification of the monoclonal antibody

Note 5. Nucleotide sequences of the antibody candidates

Note 1. Algorithm of SCIGA

Quality control of reads

SCIGA trims low-quality reads using Trimmomatic which is embedded in the SCIGA software. SCIGA allows users to set up the criteria for quality control, including the size of the sliding window for trimming reads (default value is 4), the cut off value for the average quality score in a sliding window (default value is 15), and the cut off for the length of reads after trimming (default value is 75).

Cell calling

In the 10X system, the majority (~90–99%) of generated GEMs contain no cell. We need to detect the cell-containing GEMs base on the read counts. SCIGA trims the first 39 bases of read 1 containing the 16-nt cell barcode, 10-nt unique molecular identifier (UMI) and 13-nt switch oligonucleotide as described previously [7]. The barcode and UMI are retained for each read. Reads with identical cell barcodes are considered as being derived from the same cell.

SCIGA calculates the read number per barcode and ranks these barcodes by read number in reverse order. The barcodes not in the top 10% are discarded, since at least 90% of GEMs contain no cell. The barcodes with read number over a given threshold are retained. SCIGA provides two methods to help choose the threshold as follows: 1) SCIGA is used to construct a curve where the rank of barcodes is used as x-axis information and the read count of the barcode serves as y-axis information. The threshold is set at the point where the gradient is minimal. This method is suitable for samples having large differences in read counts between “real” cells and background (Fig. S6A); 2) The threshold is set arbitrarily (default value is 200) (Fig. S6B).

Immunoglobulin sequence assembly

If the read number for a given barcode exceeds 80,000, it is downsampled to 80,000. SCIGA performs immunoglobulin assembly for each barcode separately using SSAKE, a reliable de novo assembler for short reads that is embedded in the SCIGA software. The trimmed reads 1 and reads 2 are used as input for SSAKE with the parameter “-w 5 -p 1 -c 1”. Contiguous sequences with length less than 300 bases or coverage less than a given threshold (default value is 3) are discarded.

Making a reference database

A reference database (embedded in SCIGA) is needed before determining the usage of the V-, (D-), J- gene of the assembled immunoglobulin sequences. The ungapped nucleotide sequences of all V-, D-, J-, and C-gene segments of

heavy, kappa, and lambda chains were downloaded from the international immunogenetics information system (www.imgt.org). The reference database contained human, mouse, and rat sequences. Most C-genes had identical gene names but had different sequences. Tags were added to the gene names to distinguish them from each other. The indexes of V-, D-, J- sequences were built by IgBLAST and indexes of C- sequences were built by BLAST.

V(D)JC gene calling

The usage of the V-, (D-), J- genes are determined. For this purpose, SCIGA aligns the high-quality contiguous sequences against the V-, D-, J- gene reference database using IgBLAST with the parameter "-evalue 0.001". To determine the isotype usage, SCIGA aligns the contiguous sequences against the C-gene reference database using BLAST with the parameter "-evalue 0.001". SCIGA only retains the alignments with the highest score.

Quality control of immunoglobulin sequences

SCIGA sets up several quality-control steps to obtain complete V(D)J sequences, as follows: 1) The V(D)J sequences that cannot be assigned to certain V- or J- genes are discarded; 2) The V(D)J sequences that fail to identify the CDR3 region are discarded; 3) The V(D)J sequences must be in the correct reading frame and have no stop codon; 4) The V(D)J nucleotide sequences should be aligned to the first position of the V-gene to ensure the intactness of the FR1 region. 5) The V(D)J amino sequences should include the first four positions of the FR4 region, as previously defined [20], to ensure the intactness

of the CDR3 region. The first four positions should be the conserved motifs of XGXG, WSQG (heavy chain), FGXG (light chains), or FSDG (kappa chain).

B cell quality control

Typically, a B cell has one heavy and one light chain. However, after immunoglobulin sequence assembly and quality control, some cells have multiple heavy or light chains (This may be due to the contamination of free RNA or multiple cells in GEMs). The other cells have only one chain which may be due to the low sequencing depth. For each cell, SCIGA reports the heavy and light chain with the highest UMIs number. For each reported heavy (light) chain, SCIGA calculates a certainty score, which is defined as the number of UMIs supporting the chain divided by the total number of UMIs of all heavy (light) chains for that cell [7]. The chains with a certainty score less than a given threshold (default value is 80%) are discarded. Finally, the cells without paired heavy and light chains are filtered out.

Clonal lineage grouping

SCIGA defines the cells as clonal lineage when the cells have identical V_H, J_H, V_L and J_L genes, identical H-CDR3 length, and over a given similarity threshold (default value is 90%) of H-CDR3 nucleotide sequences [7]. SCIGA implements this step by using a custom script as follows: 1) Group the cells with identical V_H, J_H, V_L and J_L genes and identical H-CDR3 length into a cluster; 2) Merge the identical H-CDR3 nucleotide sequences for each cluster into a unique representative sequence and calculate the abundance of the representative

sequence. Next, rank the representative sequences by abundance in the reverse order. 3) Perform an iteration process: the first representative sequence serves as the centroid of the first clone. Next sequentially compare the given nucleotide sequence to the centroids of all existing clones and calculate the identity scores. If the maximum identity score is more than a given similarity threshold (default value is 90%), SCIGA assigns the given sequence to the clone with the maximum identity score, or assigns it to a new clone as the centroid.

Statistical analysis and visualization

SCIGA computes a list of statistics. Some of them are calculated as below:

Gene usage frequency is calculated as $\frac{gene\ usage\ count}{total\ cell\ count} \times 100\%$.

SHM rate of the V(D)J gene is calculated as $\frac{mismatchs\ in\ gene}{gene\ length} \times 100\%$.

Simpson index is calculated as $\frac{\sum_{i=1}^{S} n_i(n_i-1)}{N(N-1)}$, where n_i is the number of cells of the ith clone, N is the total number of cells, and S is the total number of clones [21].

Shannon entropy is calculated as $1 - \frac{-\sum_{i=1}^{S} p_i log_2 p_i}{log_2 S}$, where p_i is the fraction of the ith clone and S is the total number of clones [22].

For visualization of the repertoires, SCIGA generates several figures to show the distribution of the V-gene usage frequency, the SHM of V-gene, the CDR3 length, and the clone frequency by using the R programming language.

Multiple sample integration analysis

After analyzing each sample, SCIGA integrates the outputs of several samples

into one and determines the shared immunoglobulin sequences. Shared immunoglobulins are defined as the immunoglobulins from different samples that can be clustered into the same clonal lineage. Clustering is performed with the cells of all samples.

Note 2. Sample processing and sequencing

PBMCs from COVID-19 convalescent patients were isolated using a Ficoll-Hypaque density gradient centrifugation protocol. The single-cell immunoglobulin (Ig) libraries were generated by using the Chromium Single Cell V(D)J Reagent Kits (10X Genomics; PN-1000006, PN-1000020, PN-120236, PN-120262) following the manufacturer's instruction. Briefly, GEMs were generated by combining barcoded single cell 5’ gel beads, a master mix containing about 20,000 PBMCs, and partitioning oil onto chromium chip A. Reverse transcription takes place inside each GEM, which produces full-length cDNA from poly-adenylated mRNA. Next full-length cDNAs were amplified for V(D)J segment enrichment via PCR amplification with primers specific to Ig constant regions. Variable length fragments that collectively span the V(D)J segments of the enriched Ig transcripts were generated via enzymatic fragmentation for library construction. The resulting libraries that comprised standard Illumina paired-end constructs were sequenced.

Note 3. Data analysis

For the analysis of each sample, we used the SCIGA to process the paired-end reads generated by sequencing, with the default parameter. The code is following:

```
sciga -fq1 <read1.fastq.gz> -fq2 <read2.fastq.gz> -outdir <output> -species
  human
```

For the integration analysis of multiple samples, we used the SCIGA to process the results of multiple samples, with the default parameter. The code is following:

```
sciga-merge -in <B1, B2 … B9> -out <output>
```

For the analysis by using Cell Ranger, the code is following:

```
cellranger vdj --id=<sample_name> --fastqs=<fastq_directory>
  --reference=<hg38_vdj> --sample=<sample_name> --denovo
```

Note 4. Identification of the monoclonal antibody

Screening the monoclonal antibody

Monoclonal antibodies were screened as follows: 1) Screen the clone with fraction ≥ 1% and cell number ≥ 20; 2) For each clone, screen the IgG immunoglobulin; 3) Trim the nucleotide sequences of immunoglobulin and retain the variable region; 4) Calculate the abundance of trimmed immunoglobulin sequences. The immunoglobulin sequence with the highest abundance in a clone is the monoclonal antibody candidate.

The expression and purification of monoclonal antibodies

The IgG heavy and light chain variable genes were synthesized and cloned into

the human full-length IgG1 expression vectors (Sangon Biotech, Shanghai). Paired heavy- and light-chain expressing plasmids were co-transfected into 293 F cells, and antibodies were purified from the cell supernatants using protein A columns according to the manufacturer's instructions (National Engineering Research Center for Biotechnology, Beijing) after 5 days. The concentration of purified monoclonal antibodies was determined using a NanoDrop spectrophotometer (Thermo Scientific).

Enzyme-linked immunosorbent assay

The recombinant extracellular domain or other subdomains of SARS-CoV-2 S protein (spike, S1, RBD, NTD, and S2, all from Sino Biological, Beijing) were coated (2 μg/ml) onto 96-well plates overnight at 4°C. The plates were blocked with the blocking buffer (phosphate buffered saline containing 5% skim milk and 2% bovine albumin) at RT for 1 h. Five-fold serial-diluted mAbs were added to the plates and subsequently incubated for 1 h at 37°C. HRP-conjugated goat anti-human IgG (ZSGB-BIO, Beijing) secondary antibody was added to the plates and incubated at 37°C for 1 h. The enzymatic reaction was developed with 3,3′,5,5′-tetramethylbenzidine (TMB) substrate (Kinghawk, Beijing) and stopped by addition of 2M H_2SO_4. The absorbance was measured at 450 nm using a Varioskan™ LUX Multimode Microplate Reader (Thermo Scientific). HIV-1-GP140 (purified in our lab) was an irrelevant antigen control.

Pseudovirus-based neutralization assay

The SARS-CoV-2 pseudovirus was generated through co-transfection of 293T cells with pVAX1-S and pNL4-3.Luc.R-E-, which carried the codon optimized SARS-CoV-2 S gene (GenBank: MN988668.1) and HIV-1 backbone,

respectively. Viral supernatant was collected at 48 h post-transfection and frozen at -80°C. The serially diluted antibodies were incubated with equal volume pseudovirus at 37°C for 1 h. The antibody-virus mixtures were subsequently added onto 96-well plates which pre-seeded HEK 293T-ACE2 cells. After 48 h, infected cells were lysed to measure the luciferase activity using Bright-Glo Luciferase (Promega, Madison, WI) according to the manufacturer's protocol. The IC_{50} was determined by GraphPad Prism 7 using asymmetric (five parameters) model.

Focus reduction neutralization test

SARS-CoV-2 focus reduction neutralization test (FRNT) was performed in a certified Biosafety level 3 lab. Antibodies were 3-fold serially diluted and mixed with equal volume of SARS-CoV-2 live virus (containing 200 focus forming unit) on U-bottom 96-well plates. The mixtures were incubated for 60 min at 37 ^{o}C and next transferred onto the 96-well plate seeded with Vero E6 cells for 1 h at 37 ^{o}C before removed. After washing, the overlay media (MEM containing 1.6% Carboxymethylcellulose, 2% fetal bovine serum) was added and cells were incubated at 37 ^{o}C for 24 h. After removing the overlay media, cells were fixed with 4% paraformaldehyde solution, permeabilized with Perm/Wash buffer (BD Biosciences) containing 0.1% Triton X-100, incubated with HRP-conjugated anti-SARS-CoV-2-N IgG (isolated in our lab). The reactions were developed with KPL TrueBlue Peroxidase substrates (Seracare Life Sciences Inc). The numbers of SARS-CoV-2 foci were calculated using an EliSpot reader (Cellular Technology Ltd).

Note 5. Nucleotide sequences of the antibody candidates

Paired heavy and light chains of the antibodies are shown as follows:

>B2-C1_IGH

CAGGTGCAGCTACAGCAGTGGGGCGCGGGACTGTTGAAGCCTTCGGAGACCCTGT
CCCTCACCTGCGCTGTCTATGGTGTGTCGCCCACTAGTTACTATTGGAGCTGGATCC
GTCAGTCCCCCGGGAAGGGTCTGGAGTGGATAGGGGAGATCACTCATAGTGGAAGC
ACCAACTACAATCCGTCCCTCAAGAGTCGAGTCACCATGTCGCTGGACACGTCCAA
GAGCCAGTTCTCCCTGAAGTTGAGTTCTGTGACCGCCGCGGACACGGCTATATATTA
TTGTGCGAGGGGACGCAGTGAGGAGACCATGATAGTGATGGTTGTCACGGGAATTG
ATTTCTACTTTGACTCTTGGGGCCAGGGGACCCTGGTCACCGTCTCCTCA

>**B2-C1_IGL**

TCTTCTGAGCTGACTCAGGACCCTGCTGTGTCTGTGGCCTTGGGACAGACAGTCAG
GATCACATGCCAAGGAGACAACCTCAAACTCTTTTATACAAACTGGTACCAGCAGAA
GCCAGGCCAGGCCCCCGTACTTGTCATCCATGGTAAAAACAACCGGCCCTCAGGGA
TCCCAGACCGATTCTCTGGCTCCAGTTCAGCGTACACCACTTCCTTGACCATCATTG
GGGCTCAGGCGGAGGATGAGGCTGACTATTACTGTAGCTCTCGCGACAGAAGTGGT
GACCGTGTTATATTCGGCGGAGGGACCAAGGTGACTGTCCTA

>B6-C2_IGH

GAGGTGCTCCTGGTGGAGTCTGGGGGAGGCTTGGTCCGGCCTGGAGGGTCCCTAA
GACTCTCCTGTGCAGCCTCTGGATTCACCTTCACTGACCACTATTTGGACTGGGTCC
GCCAGGCTCCAGGGATGGGGCTGGAGTGGGTTGGCCGTATTAGAAATAAAGTTAAT
GGTTACACCACAGAATACGCCGCGTCTGTGAAAGGCAGATTCACCATCTCAAGAGAT

GATTCAAAGAACTCAGTTTATCTGCAAATGAATAGCCTGAGAAGCGAGGACACGGCC
GTGTATTACTGCACTAGAGTGGGAGTTGGGAGCCCTGACTACTGGGGCCAGGGAAC
CCTGGTCGCCGTCTCCTCA

>B6-C2_IGK

GACATCCAGATGACCCAGTCTCCATCCTCCCTGTCTGCATCTGTAGGAGACAGAGTC
ACCATCACTTGCCGGGCAAGTCAGGGCATTAGAGATGAGTTAGCCTGGTATCAGCAA
AAACCAGGGAAAGCCCCTAAGCGCCTGATCTATGATGCATCGAGGTTGCAAAGTGG
GATCCCATCGAGGTTCAGCGGCAGTGGATCTGGGACAGAATTCACTCTCACAATCAG
CAGTCTGCAGCCTGAAGATTTTGCAACTTATCATTGTCTACAGTATACTAGTTACCCTC
ACACTTTTGGCCAGGGGACCAAGCTGGACATCAAA

>B6-C3_IGH

CAGGTGCAGCTACAACAGTGGGGCGCAGGACTGTTGAAGCCTTCGGAGACCCTGT
CCCTCACCTGCGCTGTCTATGGTGGGTCCTTCAGTGGTTACCAGTGGAGGTGGATC
CGCCAGGCCCCAGGGAAGGGGCTGGAGTGGATTGGGGAAATCAATCATAGTGGAA
GCACCAATTACAACCCGTCCCTCAAGAGTCGAGTCACCATATCAGTAGACACGTCCA
AGAACCAGTTCTCCCTGAGGTTGAGGTCTGTGACCGCCGCGGACACGTCTGTGTAT
TTCTGTGCGAGAGGCCAAAATGGAGTAGTTCCAGCTCCTGTATTGGGGATCGGACCT
TACTACACCTACTCCTACATGGACGTCTGGGGCACAGGGACCACGGTCAGTGTCTC
CTCA

>B6-C3_IGL

TCTTCTGAGCTGACTCAGGACCCTGCTGTGTCTGTGGCCTTGGGACAGACAGTCAG
GATCACATGCCAAGGAGACAGCCTCAGAAGCTATTATGCAAGTTGGTACCAGCAGAA

GCCAAGACAGGCCCCTATTCTTGTCATCTATGGTAAAAACAATCGACCCTCAGGGATC
CCGGACCGATTCTCTGGCTCCTACTCAGGAGCCACAGCTTCCTTAACCATCACTGGG
GCTCAGGCGGAGGATGAGGCTGACTATTATTGTGACTCCCGGGACAGCAGTGGTAA
CCATCGAGTGTTCGGCAGAGGGACCACGGTGACCGTGCTA

>B8-C1_IGH

CAGGTGCAACTGGTGCAGTCTGGGGCTGAGGTGAAGAAGCCTGGGTCCTCGGTGA
GGGTCTCCTGCCAGGCTTCTGGAGACACCTTCAGCAACTATGCTTTCAGTTGGGTG
CGACAGGCCCCTGGACAAGGGCTTGAGTGGATGGGAAGGATCATCCCTATCTTTGG
AACACCAAACTACGCACAGAGGTTCCAGGGGAGAGTCACGATTACCGCGGACGAGT
CTACGAGGACAGCCTACATGGAATTGACCGGCCTGAGGTCTGACGACACGGCCGTG
TATTACTGTGCGAGACACACTTTGGTGACTGCTATTCAGAAGTGGGGCCAGGGAACC
CTGGTCACCGTCTCCTCA

>B8-C1_IGK

GACATCCAGATGACCCAGTCTCCTTCCACCCTGTCTGCGTCTGTTGGAGACAGAGT
CACCATCACTTGCCGGGCCAGTCAGAGTGTTAGTGACTGGTTGGCCTGGTATCAGC
AGAAACCAGGGGAGCCCCCTAAGCTCCTCATCTCTAGGGCATCTACTTTAGAGATTG
GGGTCGCATCAAGGTTCAGCGGCAGTGGATCTGGGACAGAATTCACTCTCACCATC
AGCAGCCTGCAGCCTGATGATTATGCAACTTATTACTGCCAACAGTATAATACTTATTC
GCTCACTTTCGGCGGAGGGACCAAGGTGGAGATCAAA

 Click here to access/download;Figure;all_figures_and_table.pdf

Figure 1

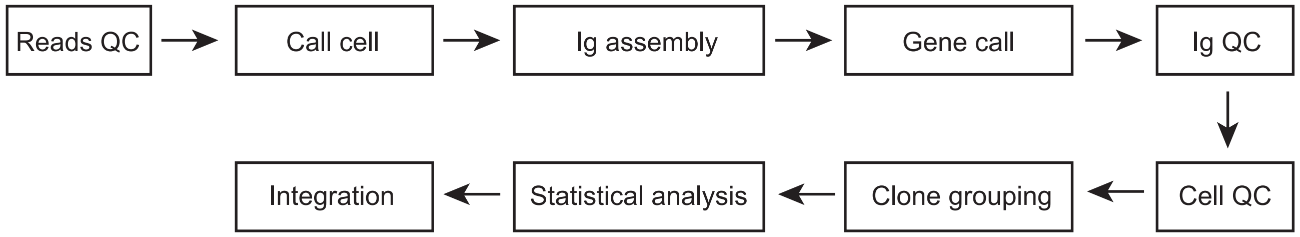

Figure 2

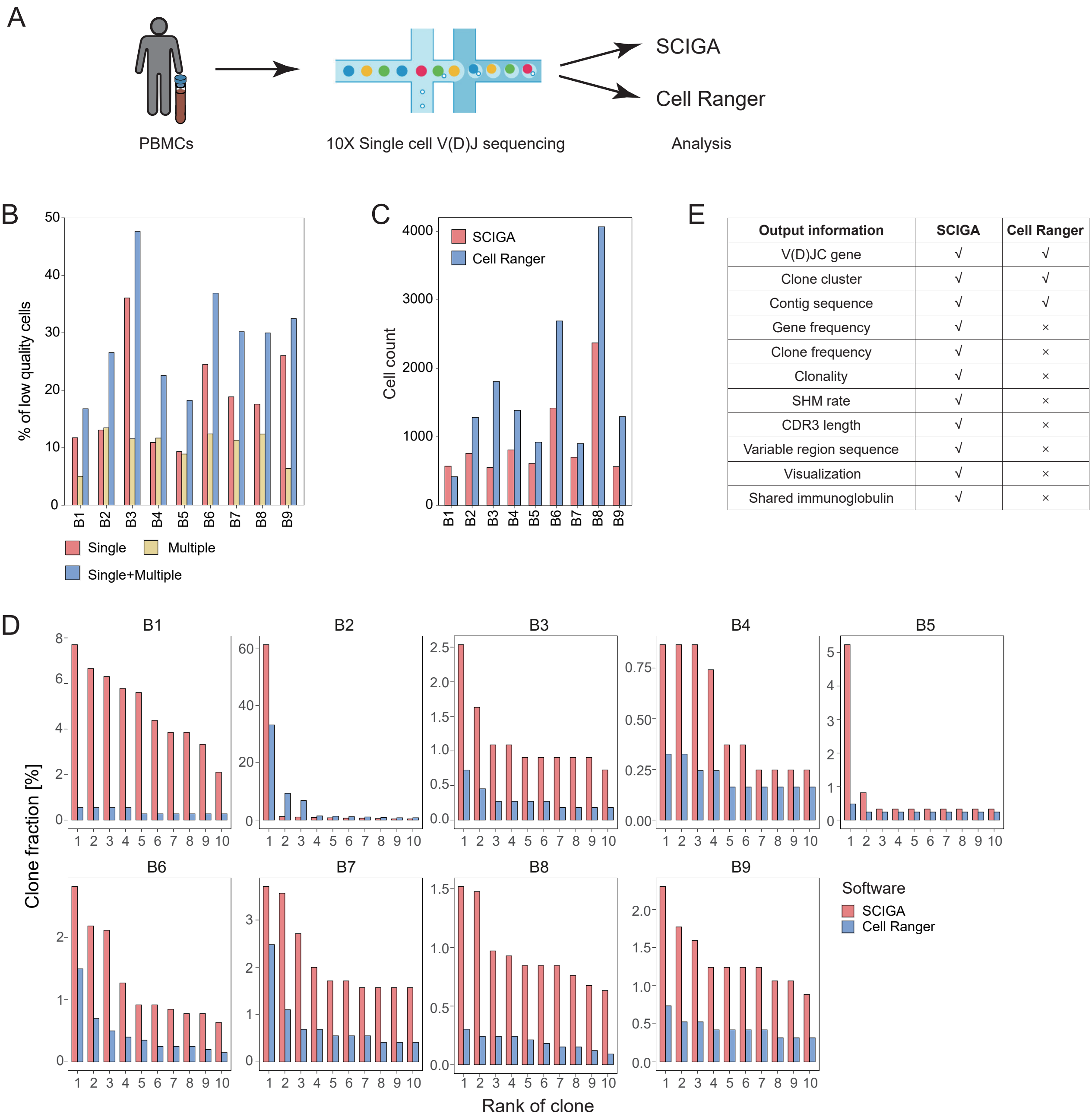

Output information	SCIGA	Cell Ranger
V(D)JC gene	√	√
Clone cluster	√	√
Contig sequence	√	√
Gene frequency	√	×
Clone frequency	√	×
Clonality	√	×
SHM rate	√	×
CDR3 length	√	×
Variable region sequence	√	×
Visualization	√	×
Shared immunoglobulin	√	×

Figure 3

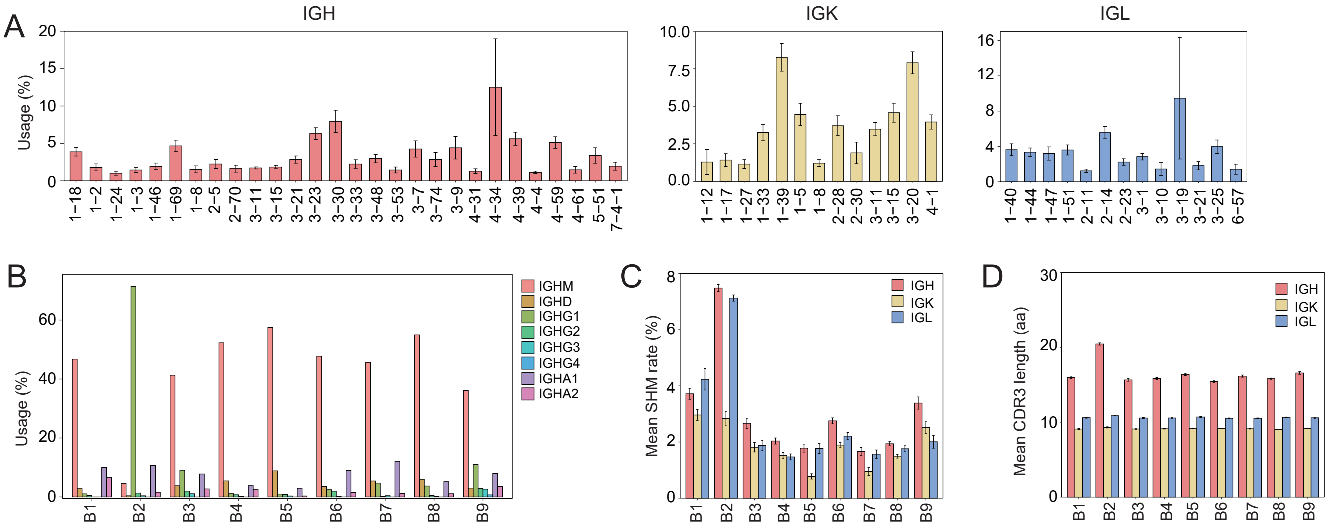

A
IGH
IGK
IGL
Usage (%)
B
Usage (%)
IGHM
IGHD
IGHG1
IGHG2
IGHG3
IGHG4
IGHA1
IGHA2
C
Mean SHM rate (%)
IGH
IGK
IGL
D
Mean CDR3 length (aa)
IGH
IGK
IGL

Figure 4

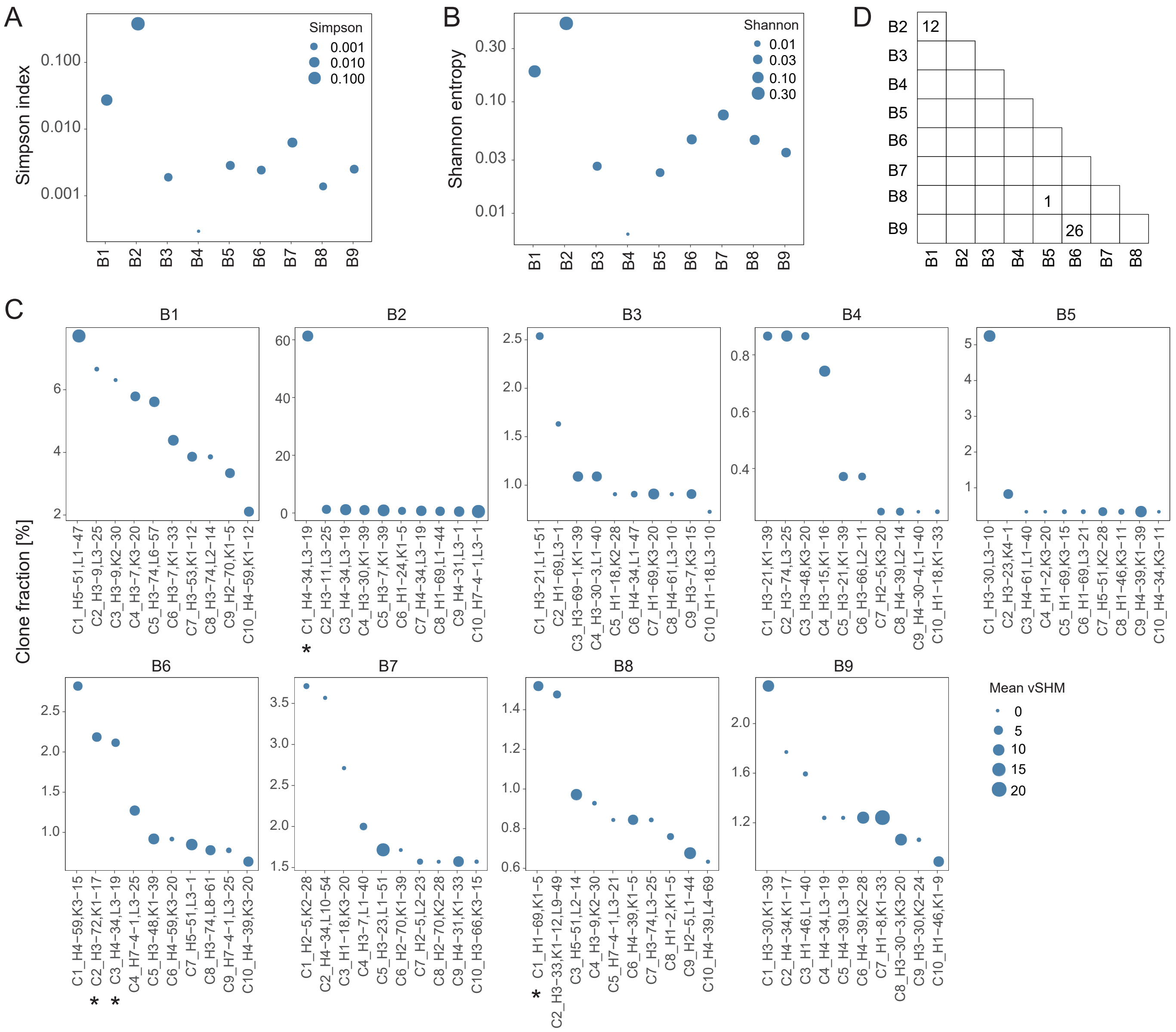
A
Simpson index
Simpson
0.001
0.010
0.100
B
Shannon entropy
Shannon
0.01
0.03
0.10
0.30
C
Clone fraction [%]
Mean vSHM
D
12
1
26
B1 B2 B3 B4 B5 B6 B7 B8 B9

Figure 5

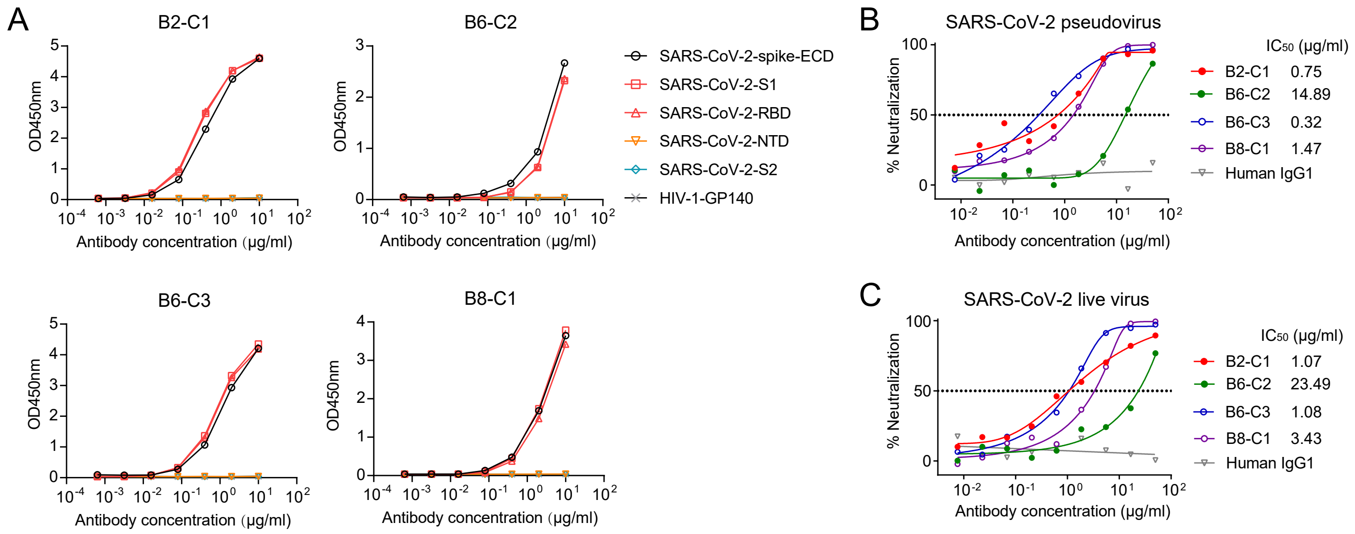
A
B2-C1
B6-C2
B6-C3
B8-C1
OD450nm
Antibody concentration (μg/ml)
SARS-CoV-2-spike-ECD
SARS-CoV-2-S1
SARS-CoV-2-RBD
SARS-CoV-2-NTD
SARS-CoV-2-S2
HIV-1-GP140
B
SARS-CoV-2 pseudovirus
% Neutralization
IC50 (μg/ml)
B2-C1 0.75
B6-C2 14.89
B6-C3 0.32
B8-C1 1.47
Human IgG1
C
SARS-CoV-2 live virus
% Neutralization
IC50 (μg/ml)
B2-C1 1.07
B6-C2 23.49
B6-C3 1.08
B8-C1 3.43
Human IgG1

Figure S1

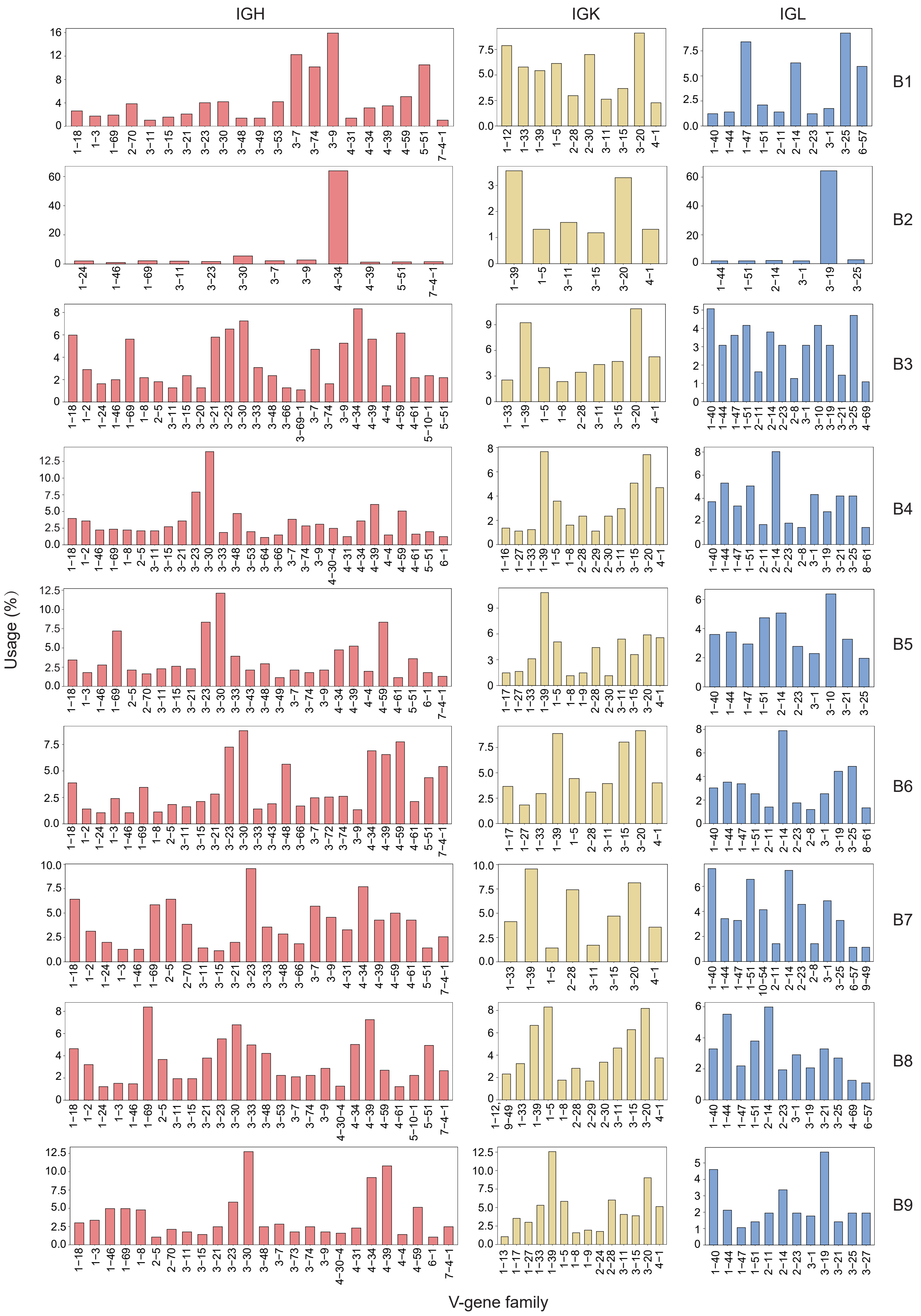

Figure S2

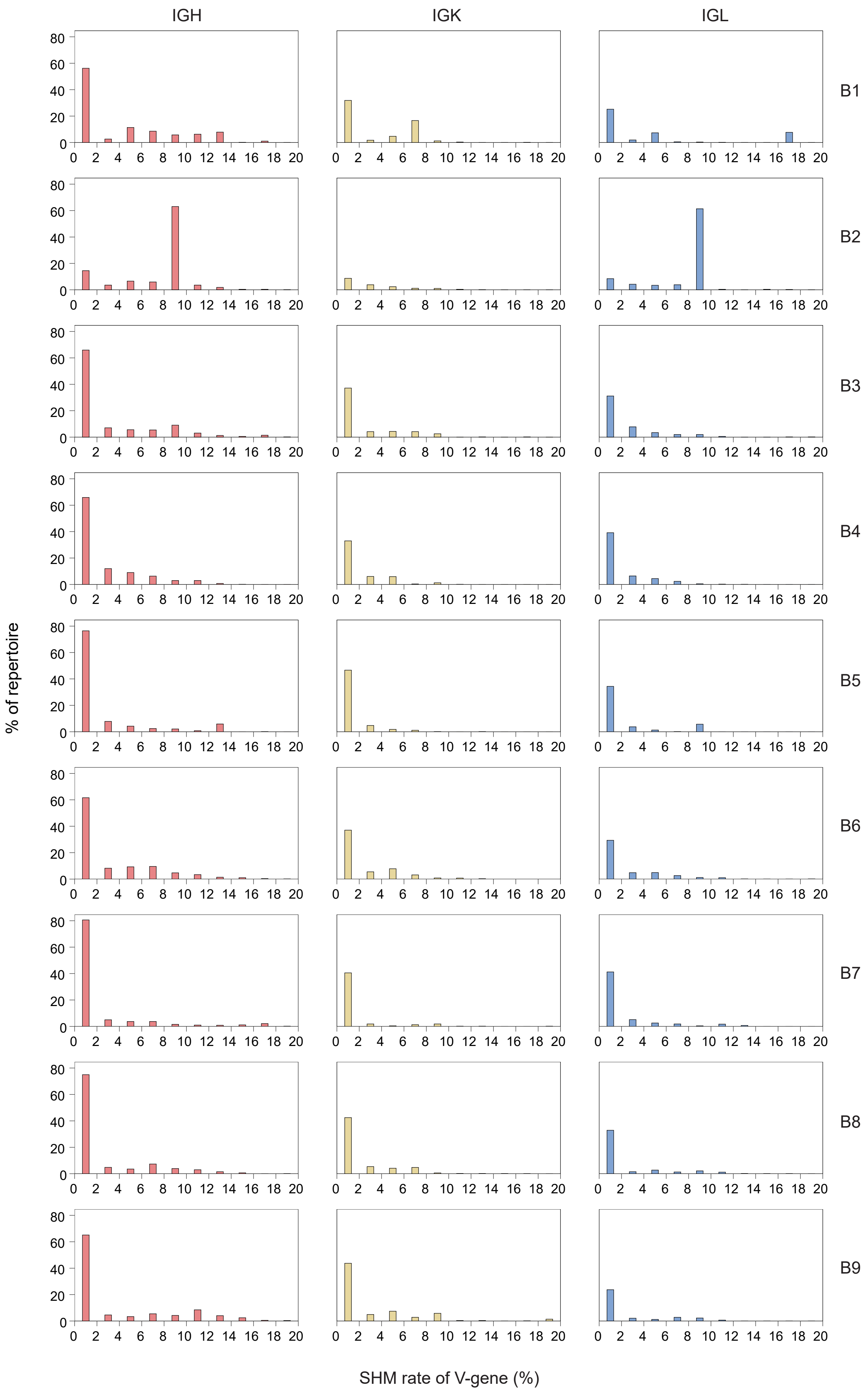
IGH
IGK
IGL
B1
B2
B3
B4
B5
B6
B7
B8
B9
% of repertoire
SHM rate of V-gene (%)

Figure S3

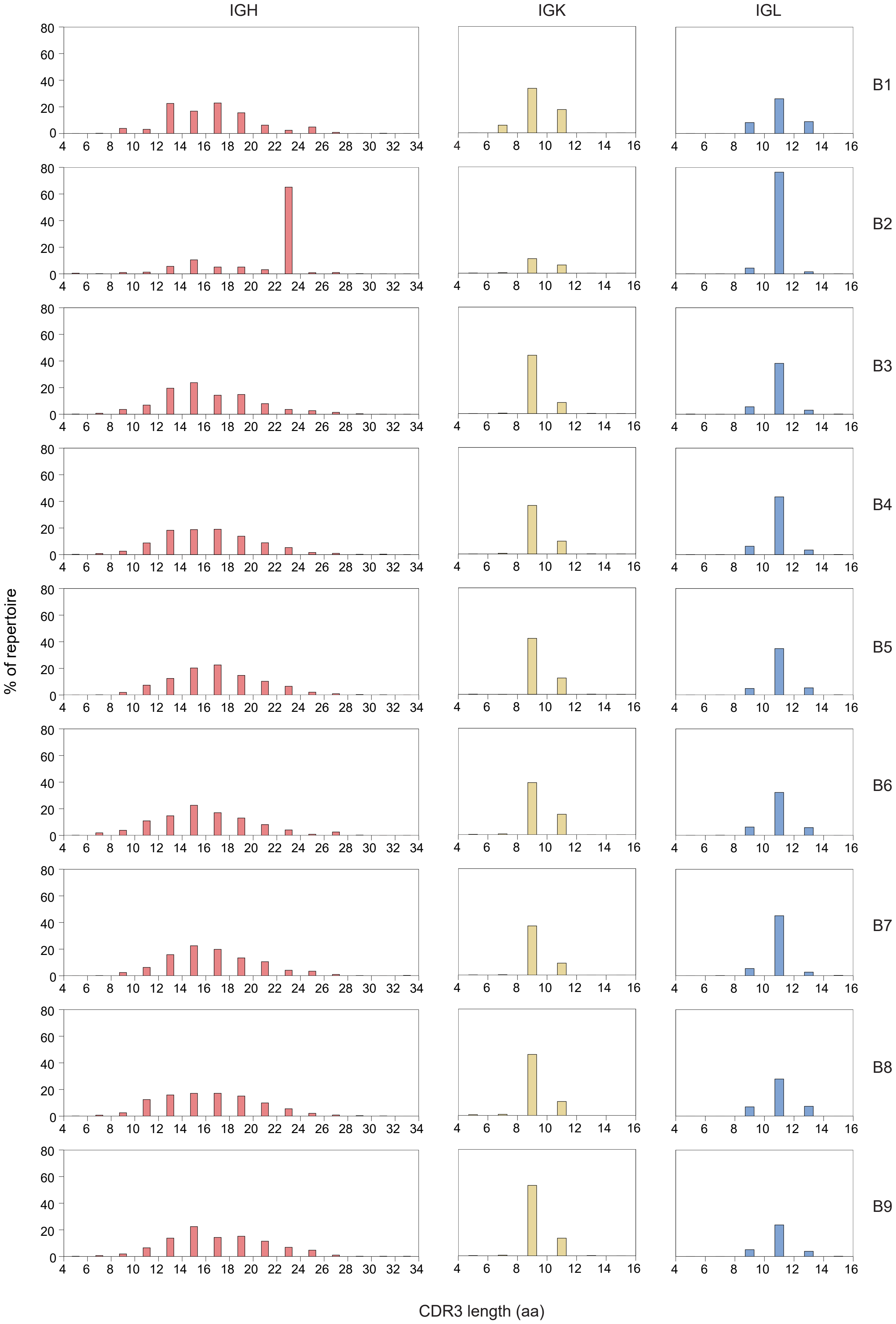

IGH
IGK
IGL
B1
B2
B3
B4
B5
B6
B7
B8
B9
0
20
40
60
80
4 6 8 10 12 14 16 18 20 22 24 26 28 30 32 34
4 6 8 10 12 14 16
% of repertoire
CDR3 length (aa)

Figure S4

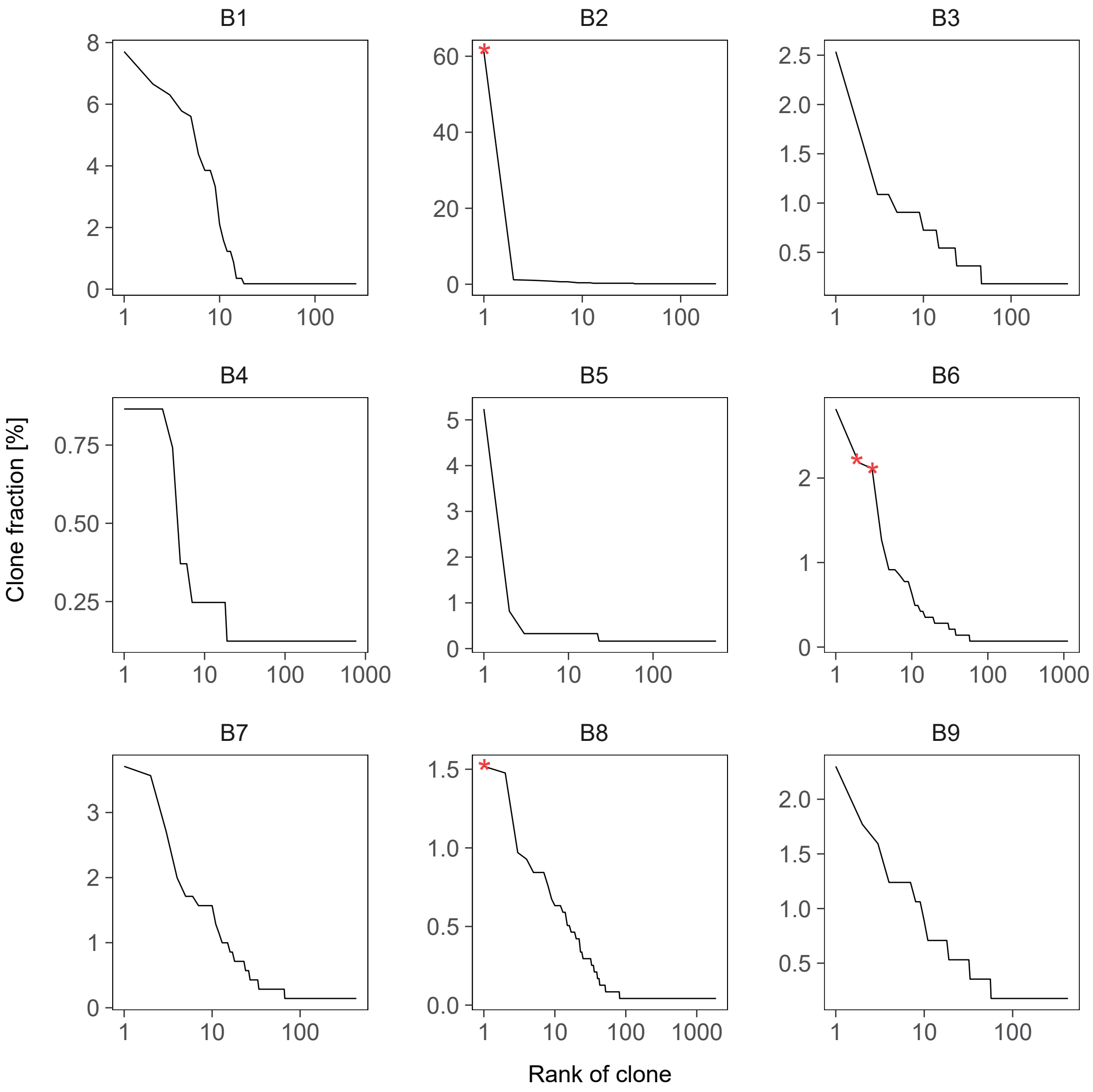

Figure S5

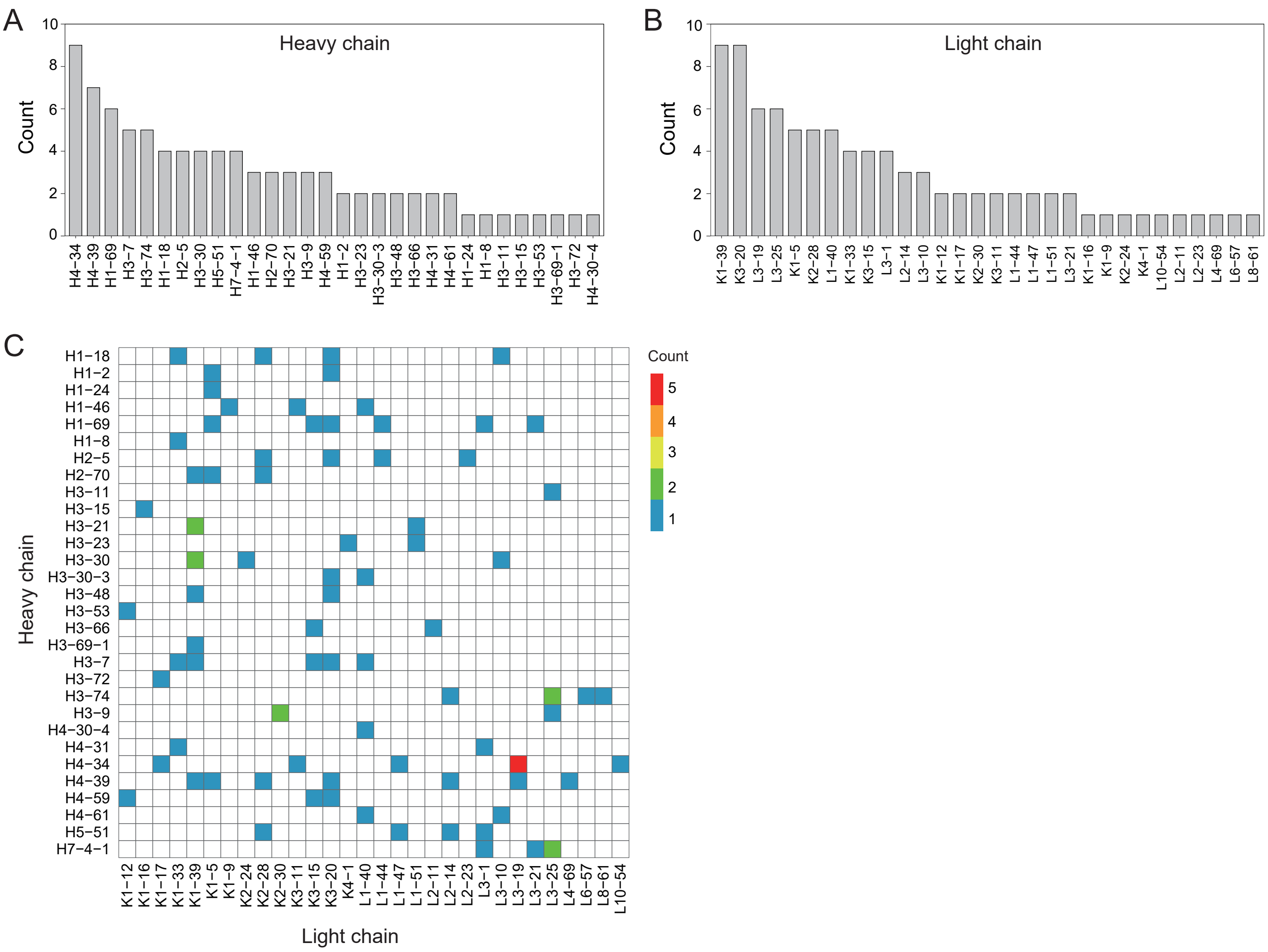

A
Heavy chain
Count
H4−34
H4−39
H1−69
H3−7
H3−74
H1−18
H2−5
H3−30
H5−51
H7−4−1
H1−46
H2−70
H3−21
H3−9
H4−59
H1−2
H3−23
H3−30−3
H3−48
H3−66
H4−31
H4−61
H1−24
H1−8
H3−11
H3−15
H3−53
H3−69−1
H3−72
H4−30−4
B
Light chain
Count
K1−39
K3−20
L3−19
L3−25
K1−5
K2−28
L1−40
K1−33
K3−15
L3−1
L2−14
L3−10
K1−12
K1−17
K2−30
K3−11
L1−44
L1−47
L1−51
L3−21
K1−16
K1−9
K2−24
K4−1
L10−54
L2−11
L2−23
L4−69
L6−57
L8−61
C
Heavy chain
Light chain
Count

Figure S6

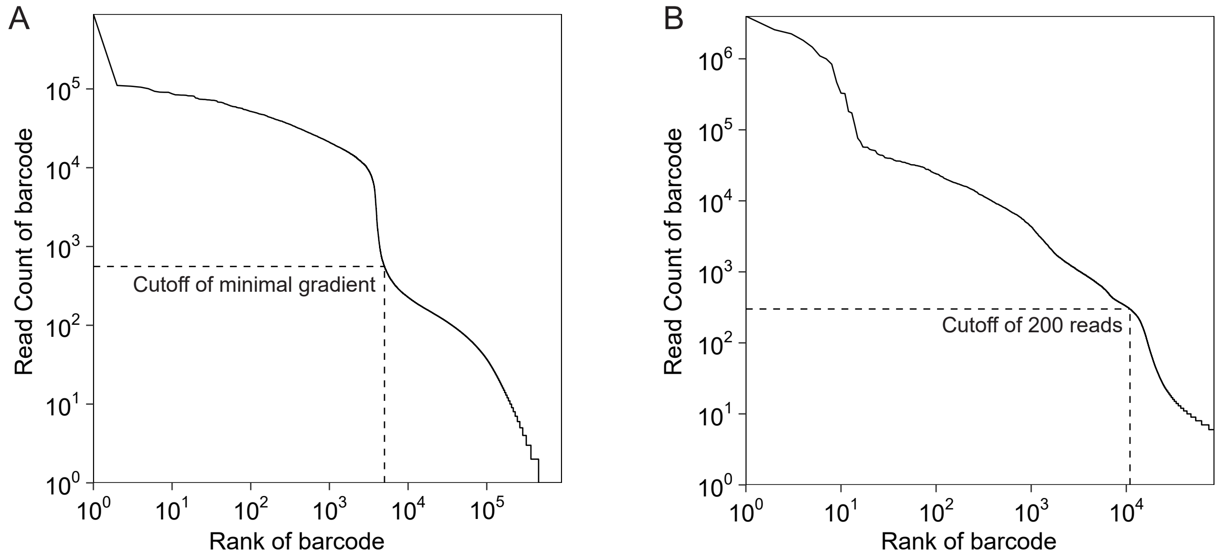
A
Read Count of barcode
10^5
10^4
10^3
10^2
10^1
10^0
Cutoff of minimal gradient
10^0 10^1 10^2 10^3 10^4 10^5
Rank of barcode
B
Read Count of barcode
10^6
10^5
10^4
10^3
10^2
10^1
10^0
Cutoff of 200 reads
10^0 10^1 10^2 10^3 10^4
Rank of barcode

Table S1

Patient ID	Gender	Age	Type
B1	Male	73	Severe
B2	Male	46	Severe
B3	Male	67	Severe
B4	Male	35	Mild
B5	Male	36	Mild
B6	Female	65	Severe
B7	Male	62	Severe
B8	Female	57	Severe
B9	Male	66	Severe

Table S2

CloneID	CloneSize	Heavy_Vgene	Heavy_Jgene	Light_Vgene	Light_Jgene	B1	B2	B5	B6	B8	B9
2	45	IGHV5-51	IGHJ4	IGLV1-47	IGLJ3	44	1	0	0	0	0
3	42	IGHV4-59	IGHJ4	IGKV3-15	IGKJ4	0	0	0	40	0	2
5	37	IGHV3-9	IGHJ4	IGKV2-30	IGKJ3	36	1	0	0	0	0
7	35	IGHV3-7	IGHJ4	IGKV3-20	IGKJ4	33	2	0	0	0	0
9	33	IGHV3-74	IGHJ4	IGLV6-57	IGLJ2,IGLJ3	32	1	0	0	0	0
10	33	IGHV4-34	IGHJ6	IGLV3-19	IGLJ3	0	0	0	30	0	3
11	33	IGHV3-30,IGHV3-30-5	IGHJ5	IGLV3-10	IGLJ2,IGLJ3	0	0	32	0	1	0
12	32	IGHV3-72	IGHJ4	IGKV1-17	IGKJ2	0	0	0	31	0	1
14	26	IGHV3-7	IGHJ4	IGKV1-33,IGKV1D-33	IGKJ5	25	1	0	0	0	0
16	23	IGHV3-74	IGHJ6	IGLV2-14	IGLJ2,IGLJ3	22	1	0	0	0	0
17	23	IGHV3-53	IGHJ5	IGKV1D-12	IGKJ4	22	1	0	0	0	0
23	20	IGHV2-70	IGHJ4	IGKV1-5	IGKJ2	19	1	0	0	0	0
24	19	IGHV7-4-1	IGHJ4	IGLV3-25	IGLJ2,IGLJ3	0	0	0	18	0	1
34	14	IGHV3-48	IGHJ4	IGKV1-39,IGKV1D-39	IGKJ2	0	0	0	13	0	1
36	14	IGHV4-59	IGHJ5	IGKV3-20	IGKJ1	0	0	0	13	0	1
38	13	IGHV4-59	IGHJ4	IGKV1-12,IGKV1D-12	IGKJ3	12	1	0	0	0	0
43	12	IGHV7-4-1	IGHJ4	IGLV3-25	IGLJ3	0	0	0	11	0	1
44	12	IGHV3-74	IGHJ6	IGLV8-61	IGLJ3	0	0	0	11	0	1
66	8	IGHV4-34	IGHJ4	IGLV2-11	IGLJ2,IGLJ3	7	1	0	0	0	0
68	8	IGHV1-2	IGHJ3	IGKV1-5	IGKJ1	0	0	0	7	0	1
96	6	IGHV3-49	IGHJ3	IGKV1-39,IGKV1D-39	IGKJ3	5	1	0	0	0	0
98	6	IGHV3-30-3	IGHJ4	IGKV1-5	IGKJ4	0	0	0	5	0	1
100	6	IGHV1-69	IGHJ6	IGKV1-27	IGKJ3	0	0	0	5	0	1
104	6	IGHV3-48	IGHJ1	IGKV1-27	IGKJ4	0	0	0	5	0	1
108	5	IGHV4-39	IGHJ4	IGKV1-39,IGKV1D-39	IGKJ4	0	0	0	4	0	1
110	5	IGHV1-8	IGHJ4	IGLV1-47	IGLJ3	0	0	0	3	0	2
121	5	IGHV3-74	IGHJ6	IGLV1-47	IGLJ3	0	0	0	4	0	1
122	5	IGHV3-43	IGHJ5	IGKV3-15	IGKJ4	0	0	0	4	0	1
124	5	IGHV3-43	IGHJ4	IGKV4-1	IGKJ4	0	0	0	4	0	1
126	5	IGHV3-43	IGHJ6	IGKV1-39,IGKV1D-39	IGKJ1	0	0	0	4	0	1
131	5	IGHV3-23,IGHV3-23D	IGHJ4	IGLV7-46	IGLJ3	0	0	0	4	0	1
135	4	IGHV3-13	IGHJ2	IGKV4-1	IGKJ5	0	0	0	3	0	1
153	4	IGHV4-59	IGHJ4	IGKV3-20	IGKJ1	0	0	0	3	0	1
157	4	IGHV1-3	IGHJ6	IGLV1-44	IGLJ6	0	0	0	3	0	1
176	3	IGHV3-30,IGHV3-30-3	IGHJ3	IGKV3-20	IGKJ1	0	0	0	2	0	1
207	3	IGHV4-4	IGHJ4	IGLV3-19	IGLJ2,IGLJ3	0	0	0	2	0	1
289	2	IGHV3-23,IGHV3-23D	IGHJ5	IGKV1-17	IGKJ4	0	0	0	1	0	1
340	2	IGHV3-11	IGHJ6	IGKV3-20	IGKJ1	0	0	0	1	0	1
369	2	IGHV3-30,IGHV3-33	IGHJ3	IGKV1-27	IGKJ1	1	1	0	0	0	0

Table S3

Antibody	Chain	Patient ID	Clone ID	Clone fraction (%)	Clone cell number	V gene	J gene	Isotype	V gene SHM (%)	CDR3 length (aa)
B2-C1	IGH	B2	1	61.21	464	IGHV4-34	IGHJ4	IGHG	9.215	23
B2-C1	IGL	B2	1	61.21	464	IGLV3-19	IGLJ2, IGLJ3	IGLC	8.042	11
B6-C2	IGH	B6	2	2.18	31	IGHV3-72	IGHJ4	IGHG	6.645	10
B6-C2	IGK	B6	2	2.18	31	IGKV1-17	IGKJ2	IGKC	4.895	9
B6-C3	IGH	B6	3	2.11	30	IGHV4-34	IGHJ6	IGHG	3.413	26
B6-C3	IGL	B6	3	2.11	30	IGLV3-19	IGLJ3	IGLC	5.190	11
B8-C1	IGH	B8	1	1.52	36	IGHV1-69	IGHJ4, IGHJ5	IGHG	7.143	11
B8-C1	IGK	B8	1	1.52	36	IGKV1-5	IGKJ4	IGKC	6.338	9
