# Supplementary material for: SCIGA: Software for large-scale, single-cell immunoglobulin repertoire analysis
Source: Gigascience. 2021 Sep 28;10(9):giab050. doi: 10.1093/gigascience/giab050 (PMC8478610; doi:10.1093/gigascience/giab050)
Supplement: giab050_Supplemental_Files [file giab050_supplemental_files.zip › all_figures_and_table.pdf]

Figure S1

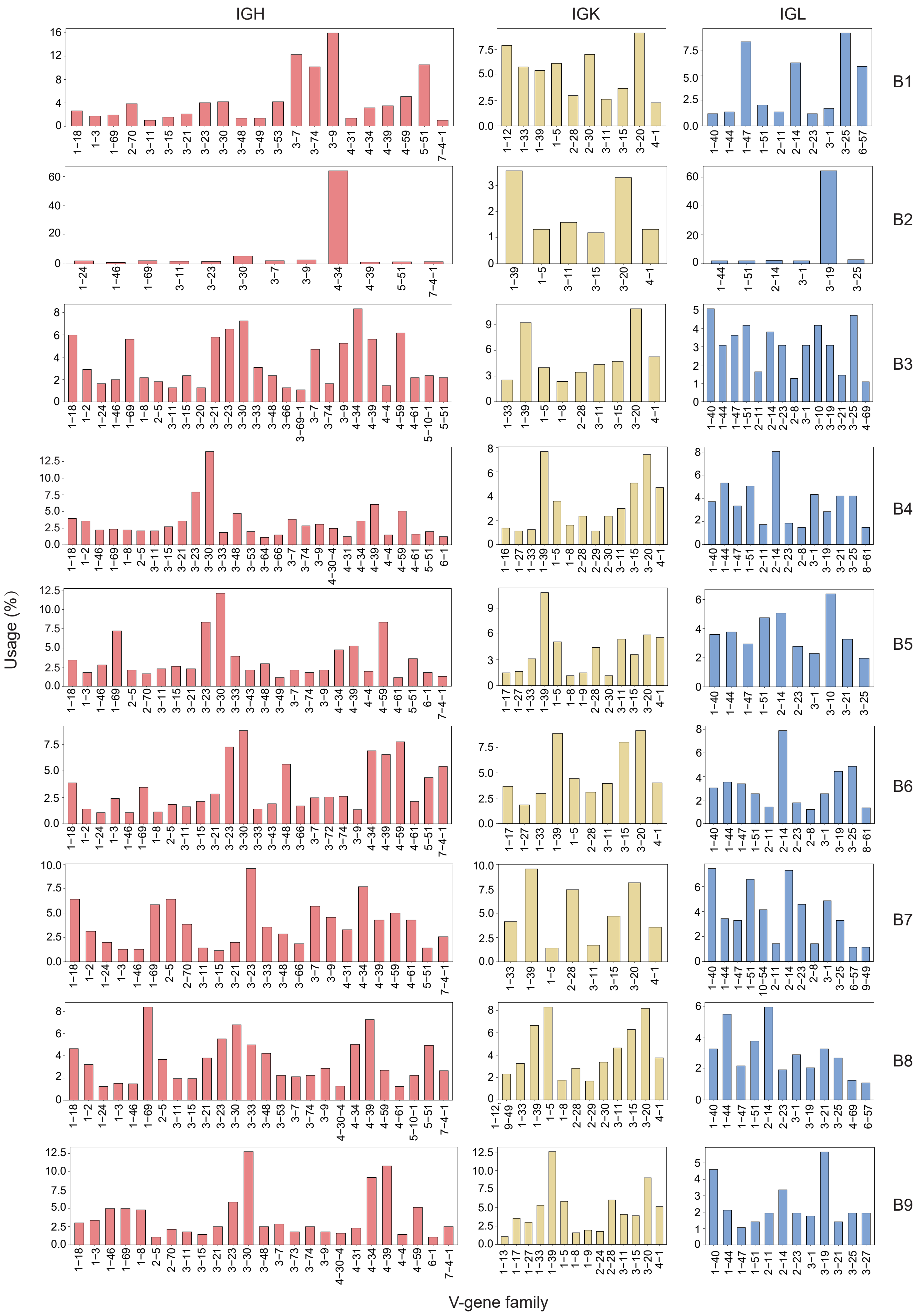

Figure S2

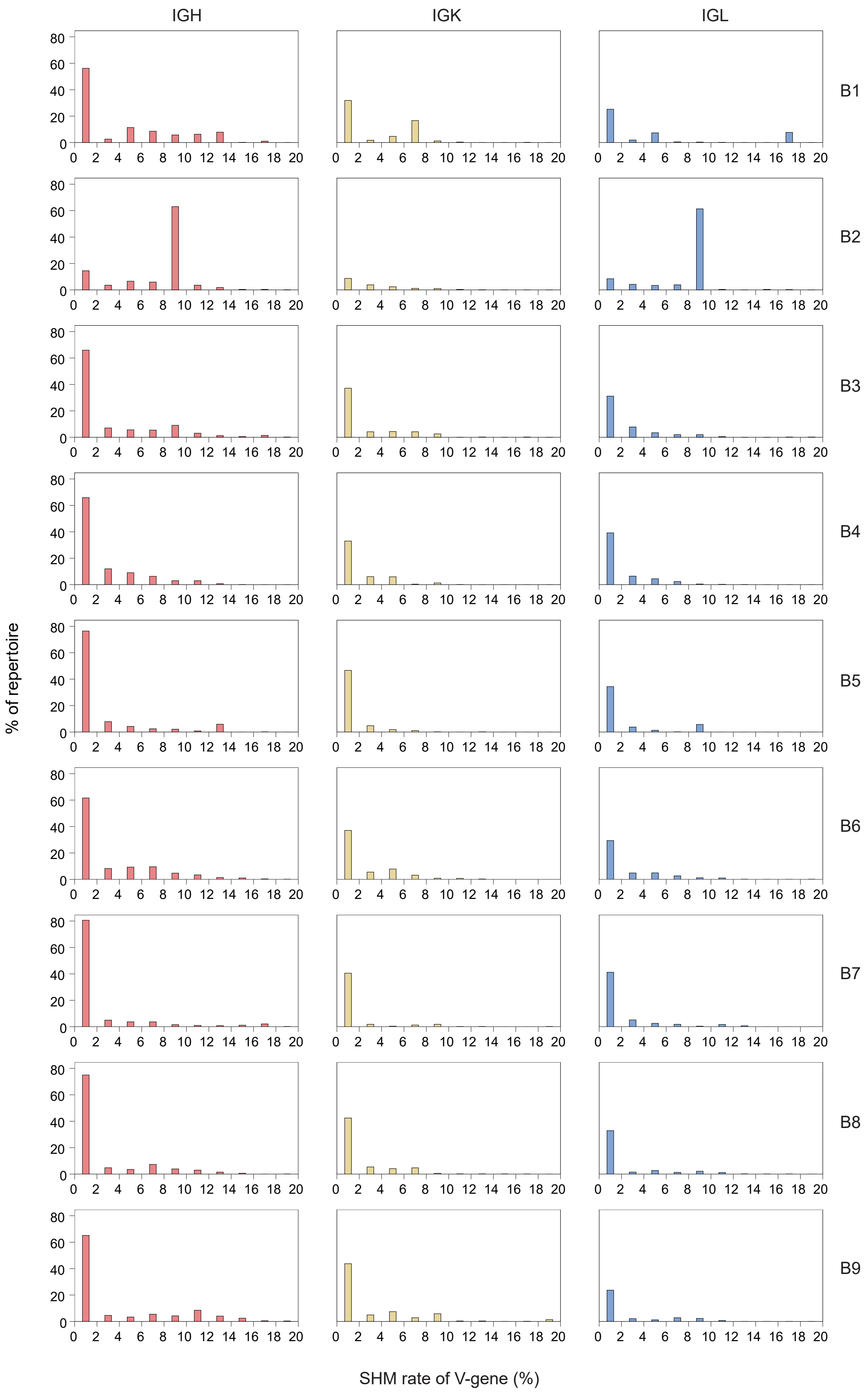
IGH
IGK
IGL
B1
B2
B3
B4
B5
B6
B7
B8
B9
% of repertoire
SHM rate of V-gene (%)
0 2 4 6 8 10 12 14 16 18 20
0
20
40
60
80

Figure S3

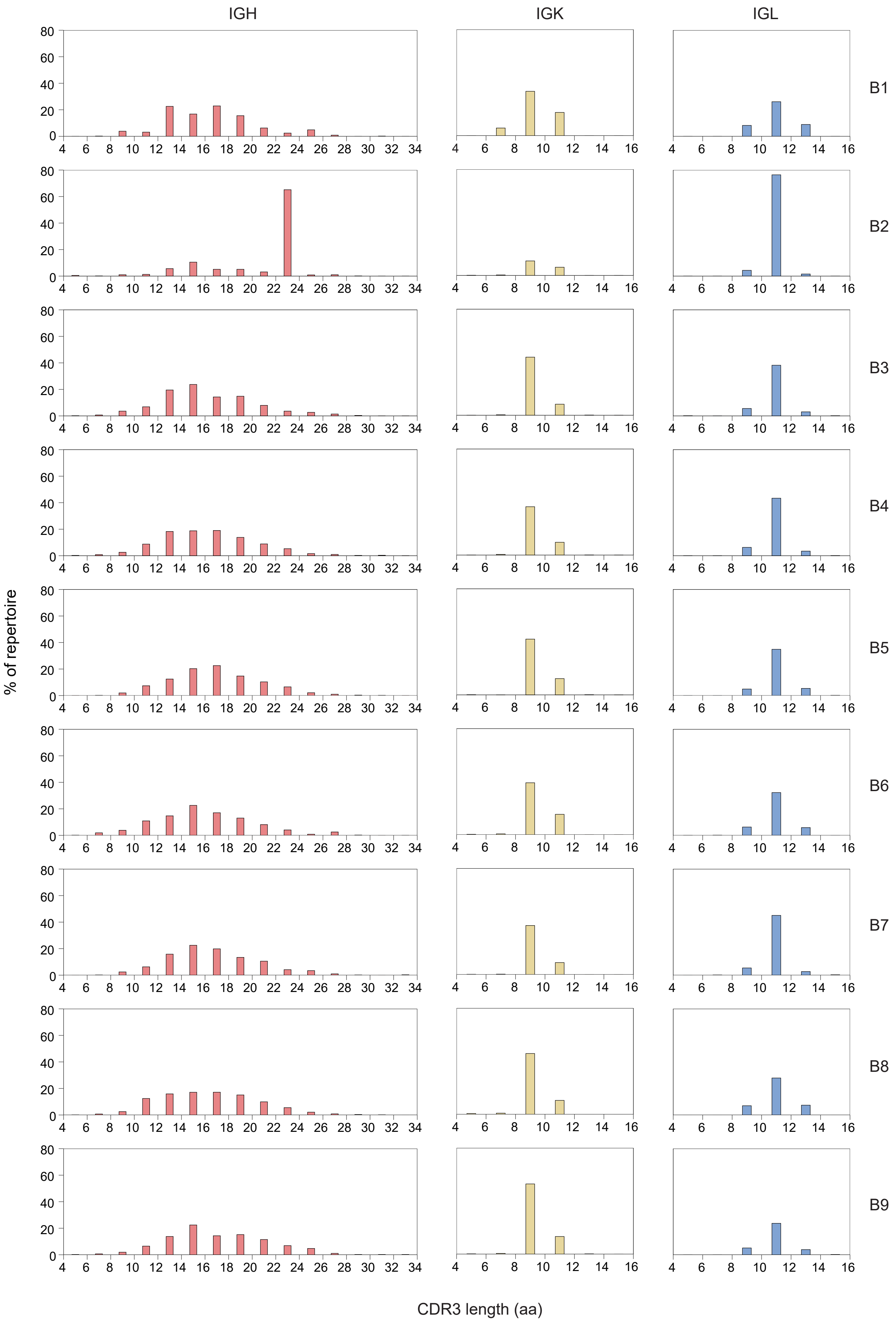
IGH
IGK
IGL
B1
B2
B3
B4
B5
B6
B7
B8
B9
% of repertoire
CDR3 length (aa)

Figure S4

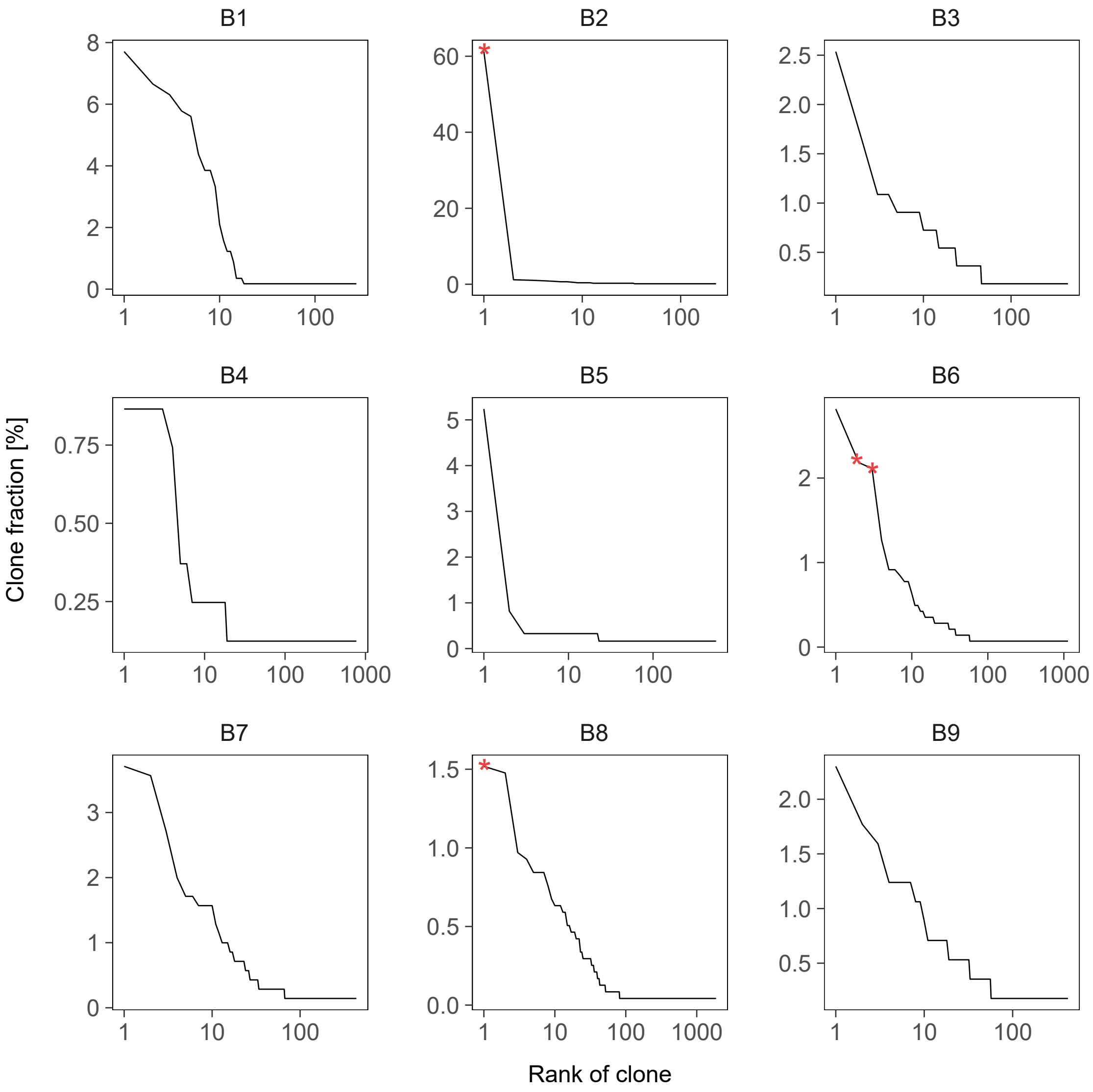

Figure S5

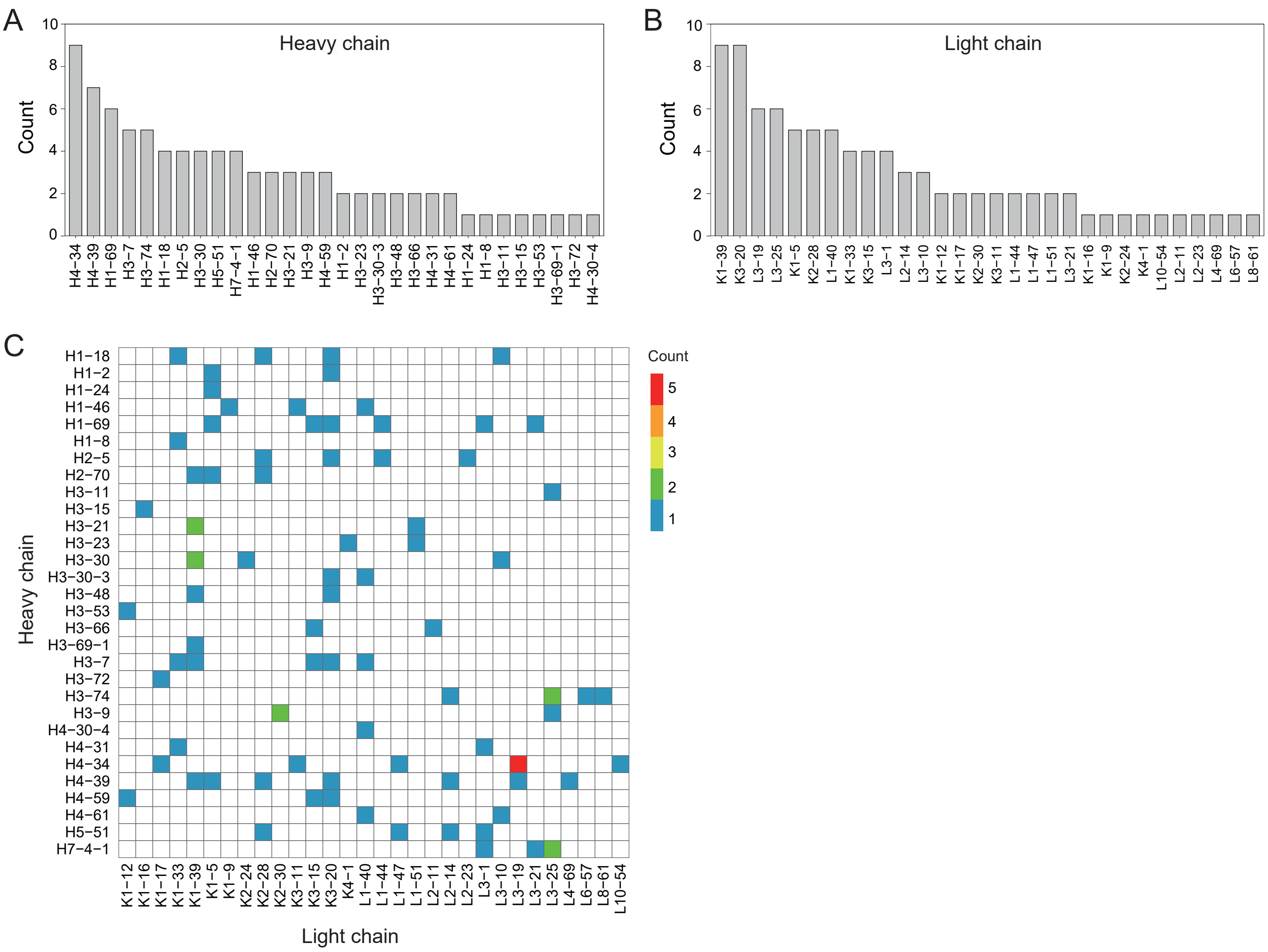
A
Heavy chain
Count
H4−34
H4−39
H1−69
H3−7
H3−74
H1−18
H2−5
H3−30
H5−51
H7−4−1
H1−46
H2−70
H3−21
H3−9
H4−59
H1−2
H3−23
H3−30−3
H3−48
H3−66
H4−31
H4−61
H1−24
H1−8
H3−11
H3−15
H3−53
H3−69−1
H3−72
H4−30−4
B
Light chain
Count
K1−39
K3−20
L3−19
L3−25
K1−5
K2−28
L1−40
K1−33
K3−15
L3−1
L2−14
L3−10
K1−12
K1−17
K2−30
K3−11
L1−44
L1−47
L1−51
L3−21
K1−16
K1−9
K2−24
K4−1
L10−54
L2−11
L2−23
L4−69
L6−57
L8−61
C
Heavy chain
Light chain
Count
5
4
3
2
1

Figure S6

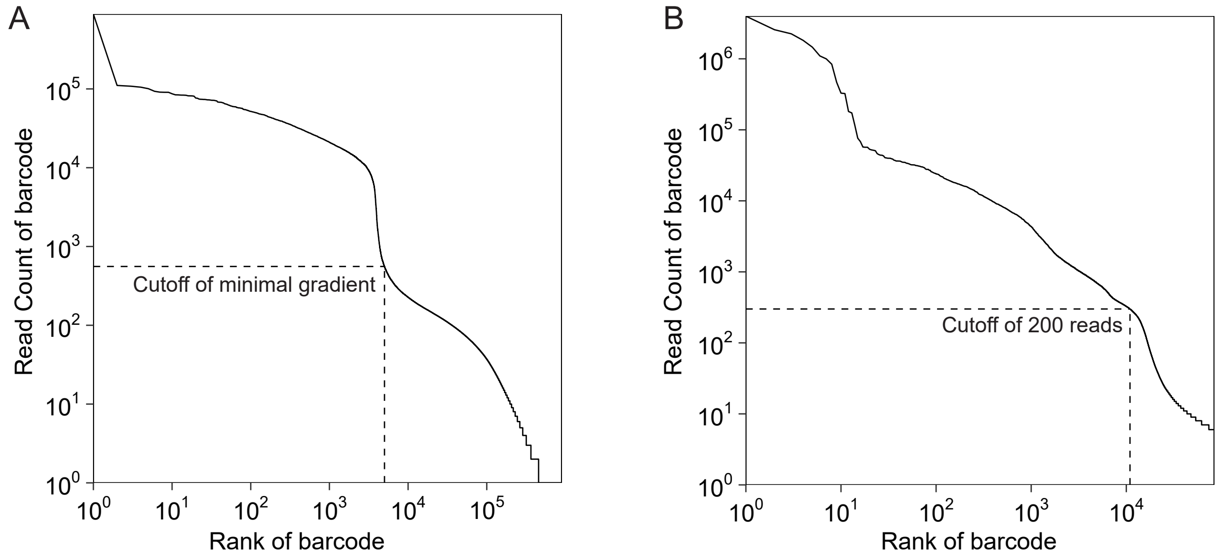
A
Read Count of barcode
10^0
10^1
10^2
10^3
10^4
10^5
Cutoff of minimal gradient
Rank of barcode
B
10^6
Cutoff of 200 reads

Table S1

Patient ID	Gender	Age	Type
B1	Male	73	Severe
B2	Male	46	Severe
B3	Male	67	Severe
B4	Male	35	Mild
B5	Male	36	Mild
B6	Female	65	Severe
B7	Male	62	Severe
B8	Female	57	Severe
B9	Male	66	Severe

Table S2

CloneID	CloneSize	Heavy_Vgene	Heavy_Jgene	Light_Vgene	Light_Jgene	B1	B2	B5	B6	B8	B9
2	45	IGHV5-51	IGHJ4	IGLV1-47	IGLJ3	44	1	0	0	0	0
3	42	IGHV4-59	IGHJ4	IGKV3-15	IGKJ4	0	0	0	40	0	2
5	37	IGHV3-9	IGHJ4	IGKV2-30	IGKJ3	36	1	0	0	0	0
7	35	IGHV3-7	IGHJ4	IGKV3-20	IGKJ4	33	2	0	0	0	0
9	33	IGHV3-74	IGHJ4	IGLV6-57	IGLJ2,IGLJ3	32	1	0	0	0	0
10	33	IGHV4-34	IGHJ6	IGLV3-19	IGLJ3	0	0	0	30	0	3
11	33	IGHV3-30,IGHV3-30-5	IGHJ5	IGLV3-10	IGLJ2,IGLJ3	0	0	32	0	1	0
12	32	IGHV3-72	IGHJ4	IGKV1-17	IGKJ2	0	0	0	31	0	1
14	26	IGHV3-7	IGHJ4	IGKV1-33,IGKV1D-33	IGKJ5	25	1	0	0	0	0
16	23	IGHV3-74	IGHJ6	IGLV2-14	IGLJ2,IGLJ3	22	1	0	0	0	0
17	23	IGHV3-53	IGHJ5	IGKV1D-12	IGKJ4	22	1	0	0	0	0
23	20	IGHV2-70	IGHJ4	IGKV1-5	IGKJ2	19	1	0	0	0	0
24	19	IGHV7-4-1	IGHJ4	IGLV3-25	IGLJ2,IGLJ3	0	0	0	18	0	1
34	14	IGHV3-48	IGHJ4	IGKV1-39,IGKV1D-39	IGKJ2	0	0	0	13	0	1
36	14	IGHV4-59	IGHJ5	IGKV3-20	IGKJ1	0	0	0	13	0	1
38	13	IGHV4-59	IGHJ4	IGKV1-12,IGKV1D-12	IGKJ3	12	1	0	0	0	0
43	12	IGHV7-4-1	IGHJ4	IGLV3-25	IGLJ3	0	0	0	11	0	1
44	12	IGHV3-74	IGHJ6	IGLV8-61	IGLJ3	0	0	0	11	0	1
66	8	IGHV4-34	IGHJ4	IGLV2-11	IGLJ2,IGLJ3	7	1	0	0	0	0
68	8	IGHV1-2	IGHJ3	IGKV1-5	IGKJ1	0	0	0	7	0	1
96	6	IGHV3-49	IGHJ3	IGKV1-39,IGKV1D-39	IGKJ3	5	1	0	0	0	0
98	6	IGHV3-30-3	IGHJ4	IGKV1-5	IGKJ4	0	0	0	5	0	1
100	6	IGHV1-69	IGHJ6	IGKV1-27	IGKJ3	0	0	0	5	0	1
104	6	IGHV3-48	IGHJ1	IGKV1-27	IGKJ4	0	0	0	5	0	1
108	5	IGHV4-39	IGHJ4	IGKV1-39,IGKV1D-39	IGKJ4	0	0	0	4	0	1
110	5	IGHV1-8	IGHJ4	IGLV1-47	IGLJ3	0	0	0	3	0	2
121	5	IGHV3-74	IGHJ6	IGLV1-47	IGLJ3	0	0	0	4	0	1
122	5	IGHV3-43	IGHJ5	IGKV3-15	IGKJ4	0	0	0	4	0	1
124	5	IGHV3-43	IGHJ4	IGKV4-1	IGKJ4	0	0	0	4	0	1
126	5	IGHV3-43	IGHJ6	IGKV1-39,IGKV1D-39	IGKJ1	0	0	0	4	0	1
131	5	IGHV3-23,IGHV3-23D	IGHJ4	IGLV7-46	IGLJ3	0	0	0	4	0	1
135	4	IGHV3-13	IGHJ2	IGKV4-1	IGKJ5	0	0	0	3	0	1
153	4	IGHV4-59	IGHJ4	IGKV3-20	IGKJ1	0	0	0	3	0	1
157	4	IGHV1-3	IGHJ6	IGLV1-44	IGLJ6	0	0	0	3	0	1
176	3	IGHV3-30,IGHV3-30-3	IGHJ3	IGKV3-20	IGKJ1	0	0	0	2	0	1
207	3	IGHV4-4	IGHJ4	IGLV3-19	IGLJ2,IGLJ3	0	0	0	2	0	1
289	2	IGHV3-23,IGHV3-23D	IGHJ5	IGKV1-17	IGKJ4	0	0	0	1	0	1
340	2	IGHV3-11	IGHJ6	IGKV3-20	IGKJ1	0	0	0	1	0	1
369	2	IGHV3-30,IGHV3-33	IGHJ3	IGKV1-27	IGKJ1	1	1	0	0	0	0

Table S3

Antibody	Chain	Patient ID	Clone ID	Clone fraction (%)	Clone cell number	V gene	J gene	Isotype	V gene SHM (%)	CDR3 length (aa)
B2-C1	IGH	B2	1	61.21	464	IGHV4-34	IGHJ4	IGHG	9.215	23
B2-C1	IGL	B2	1	61.21	464	IGLV3-19	IGLJ2, IGLJ3	IGLC	8.042	11
B6-C2	IGH	B6	2	2.18	31	IGHV3-72	IGHJ4	IGHG	6.645	10
B6-C2	IGK	B6	2	2.18	31	IGKV1-17	IGKJ2	IGKC	4.895	9
B6-C3	IGH	B6	3	2.11	30	IGHV4-34	IGHJ6	IGHG	3.413	26
B6-C3	IGL	B6	3	2.11	30	IGLV3-19	IGLJ3	IGLC	5.190	11
B8-C1	IGH	B8	1	1.52	36	IGHV1-69	IGHJ4, IGHJ5	IGHG	7.143	11
B8-C1	IGK	B8	1	1.52	36	IGKV1-5	IGKJ4	IGKC	6.338	9
