# Supplementary material for: SCIGA: Software for large-scale, single-cell immunoglobulin repertoire analysis
Source: Gigascience. 2021 Sep 28;10(9):giab050. doi: 10.1093/gigascience/giab050 (PMC8478610; doi:10.1093/gigascience/giab050)
Supplement: giab050_Supplemental_Files [file giab050_supplemental_files.zip › giab050_Online_Appendix.pdf]

Appendix

Supplementary Methods and Materials

Note 1. Algorithm of SCIGA

Note 2. Sample processing and sequencing

Note 3. Data analysis

Note 4. Identification of the monoclonal antibody

Note 5. Nucleotide sequences of the antibody candidates

Note 1. Algorithm of SCIGA

Quality control of reads

SCIGA trims low-quality reads using Trimmomatic which is embedded in the SCIGA software. SCIGA allows users to set up the criteria for quality control, including the size of the sliding window for trimming reads (default value is 4), the cut off value for the average quality score in a sliding window (default value is 15), and the cut off for the length of reads after trimming (default value is 75).

Cell calling

In the 10X system, the majority (~90–99%) of generated GEMs contain no cell. We need to detect the cell-containing GEMs base on the read counts. SCIGA trims the first 39 bases of read 1 containing the 16-nt cell barcode, 10-nt unique molecular identifier (UMI) and 13-nt switch oligonucleotide as described previously [7]. The barcode and UMI are retained for each read. Reads with identical cell barcodes are considered as being derived from the same cell. SCIGA calculates the read number per barcode and ranks these barcodes by read number in reverse order. The barcodes not in the top 10% are discarded, since at least 90% of GEMs contain no cell. The barcodes with read number over a given threshold are retained. SCIGA provides two methods to help choose the threshold as follows: 1) SCIGA is used to construct a curve where the rank of barcodes is used as x-axis information and the read count of the barcode serves as y-axis information. The threshold is set at the point where the gradient is minimal. This method is suitable for samples having large differences in read counts between "real" cells and background (Fig. S6A); 2) The threshold is set arbitrarily (default value is 200) (Fig. S6B).

Immunoglobulin sequence assembly

If the read number for a given barcode exceeds 80,000, it is downsampled to 80,000. SCIGA performs immunoglobulin assembly for each barcode separately using SSAKE, a reliable de novo assembler for short reads that is embedded in the SCIGA software. The trimmed reads 1 and reads 2 are used as input for SSAKE with the parameter "-w 5 -p 1 -c 1." Contiguous sequences with length less than 300 bases or coverage less than a given threshold (default value is 3) are discarded.

Making a reference database

A reference database (embedded in SCIGA) is needed before determining the usage of the V-, (D-), J- gene of the assembled immunoglobulin sequences. The ungapped nucleotide sequences of all V-, D-, J-, and C-gene segments of heavy, kappa, and lambda chains were downloaded from the international ImMunoGeneTics information system (www.imgt.org). The reference database contained human, mouse, and rat sequences. Most C-genes had identical gene names but had different sequences. Tags were added to the gene names to distinguish them from each other. The indexes of V-, D-, J- sequences were built by IgBLAST and indexes of C- sequences were built by BLAST.

V(D)JC gene calling

The usage of the V-, (D-), J- genes are determined. For this purpose, SCIGA aligns the high-quality contiguous sequences against the V-, D-, J- gene reference database using IgBLAST with the parameter "-evalue 0.001." To determine the isotype usage, SCIGA aligns the contiguous sequences against the C-gene reference database using BLAST with the parameter "-evalue 0.001." SCIGA only retains the alignments with the highest score.

Quality control of immunoglobulin sequences

SCIGA sets up several quality-control steps to obtain complete V(D)J sequences, as follows: 1) The V(D)J sequences that cannot be assigned to certain V- or J- genes are discarded; 2) The V(D)J sequences that fail to identify the CDR3 region are discarded; 3) The V(D)J sequences must be in the correct reading frame and have no stop codon; 4) The V(D)J nucleotide sequences should be aligned to the first position of the V-gene to ensure the intactness of the FR1 region. 5) The V(D)J amino sequences should include the first four positions of the FR4 region, as previously defined [20], to ensure the intactness of the CDR3 region. The first four positions should be the conserved motifs of XGXG, WSQG (heavy chain), FGXG (light chains), or FSDG (kappa chain).

B cell quality control

Typically, a B cell has one heavy and one light chain. However, after immunoglobulin sequence assembly and quality control, some cells have multiple heavy or light chains (This may be due to the contamination of free RNA or multiple cells in GEMs). The other cells have only one chain which may be due to the low sequencing depth. For each cell, SCIGA reports the heavy and light chain with the highest UMIs number. For each reported heavy (light) chain, SCIGA calculates a certainty score, which is defined as the number of UMIs supporting the chain divided by the total number of UMIs of all heavy (light) chains for that cell [7]. The chains with a certainty score less than a given threshold (default value is 80%) are discarded. Finally, the cells without paired heavy and light chains are filtered out.

Clonal lineage grouping

SCIGA defines the cells as clonal lineage when the cells have identical V_H, J_H, V_L and J_L genes, identical H-CDR3 length, and over a given similarity threshold (default value is 90%) of H-CDR3 nucleotide sequences [7]. SCIGA implements this step by using a custom script as follows: 1) Group the cells with identical V_H, J_H, V_L and J_L genes and identical H-CDR3 length into a cluster; 2) Merge the identical H-CDR3 nucleotide sequences for each cluster into a unique representative sequence and calculate the abundance of the representative sequence. Next, rank the representative sequences by abundance in the reverse order. 3) Perform an iteration process: the first representative sequence serves as the centroid of the first clone. Next sequentially compare the given nucleotide sequence to the centroids of all existing clones and calculate the identity scores. If the maximum identity score is more than a given similarity threshold (default value is 90%), SCIGA assigns the given sequence to the clone with the maximum identity score, or assigns it to a new clone as the centroid.

Statistical analysis and visualization

SCIGA computes a list of statistics. Some of them are calculated as below:

Gene usage frequency is calculated as $\frac{gene\ usage\ count}{total\ cell\ count} \times 100\%$.

SHM rate of the V(D)J gene is calculated as $\frac{mismatchs\ in\ gene}{gene\ length} \times 100\%$.

Simpson index is calculated as $\frac{\sum_{i=1}^{S} n_i(n_i-1)}{N(N-1)}$, where n_i is the number of cells of the ith clone, N is the total number of cells, and S is the total number of clones [21].

Shannon entropy is calculated as $1 - \frac{-\sum_{i=1}^{S} p_i log_2 p_i}{log_2 S}$, where p_i is the fraction of the ith clone and S is the total number of clones [22].

For visualization of the repertoires, SCIGA generates several figures to show the distribution of the V-gene usage frequency, the SHM of V-gene, the CDR3 length, and the clone frequency by using the R programming language.

Multiple sample integration analysis

After analyzing each sample, SCIGA integrates the outputs of several samples into one and determines the shared immunoglobulin sequences. Shared immunoglobulins are defined as the immunoglobulins from different samples that can be clustered into the same clonal lineage. Clustering is performed with the cells of all samples.

Note 2. Sample processing and sequencing

PBMCs from COVID-19 convalescent patients were isolated using a Ficoll-Hypaque density gradient centrifugation protocol. The single-cell immunoglobulin (Ig) libraries were generated by using the Chromium Single Cell V(D)J Reagent Kits (10X Genomics; PN-1000006, PN-1000020, PN-120236, PN-120262) following the manufacturer's instruction. Briefly, GEMs were generated by combining barcoded single cell 5' gel beads, a master mix containing about 20,000 PBMCs, and partitioning oil onto chromium chip A. Reverse transcription takes place inside each GEM, which produces full-length cDNA from poly-adenylated mRNA. Next full-length cDNAs were amplified for V(D)J segment enrichment via PCR amplification with primers specific to Ig constant regions. Variable length fragments that collectively span the V(D)J segments of the enriched Ig transcripts were generated via enzymatic fragmentation for library construction. The resulting libraries that comprised standard Illumina paired-end constructs were sequenced.

Note 3. Data analysis

For the analysis of each sample, we used the SCIGA to process the paired-end reads generated by sequencing, with the default parameter. The code is following:

sciga -fq1 <read1.fastq.gz> -fq2 <read2.fastq.gz> -outdir <output> -species human

For the integration analysis of multiple samples, we used the SCIGA to process the results of multiple samples, with the default parameter. The code is following:

sciga-merge -in <B1, B2 … B9> -out <output>

For the analysis by using Cell Ranger, the code is following:

cellranger vdj –id = <sample_name> –fastqs = <fastq_directory>
–reference = <hg38_vdj> –sample = <sample_name> –denovo

Note 4. Identification of the monoclonal antibody

Screening the monoclonal antibody

Monoclonal antibodies were screened as follows: 1) Screen the clone with fraction $\geq$ 1% and cell number $\geq$ 20; 2) For each clone, screen the IgG immunoglobulin; 3) Trim the nucleotide sequences of immunoglobulin and retain the variable region; 4) Calculate the abundance of trimmed immunoglobulin sequences. The immunoglobulin sequence with the highest abundance in a clone is the monoclonal antibody candidate.

The expression and purification of monoclonal antibodies

The IgG heavy and light chain variable genes were synthesized and cloned into the human full-length IgG1 expression vectors (Sangon Biotech, Shanghai). Paired heavy- and light-chain expressing plasmids were co-transfected into 293 F cells, and antibodies were purified from the cell supernatants using protein A columns according to the manufacturer's instructions (National Engineering Research Center for Biotechnology, Beijing) after 5 days. The concentration of purified monoclonal antibodies was determined using a NanoDrop spectrophotometer (Thermo Scientific).

Enzyme-linked immunosorbent assay

The recombinant extracellular domain or other subdomains of SARS-CoV-2 S protein (spike, S1, RBD, NTD, and S2, all from Sino Biological, Beijing) were coated (2 μg/ml) onto 96-well plates overnight at 4°C. The plates were blocked with the blocking buffer (phosphate buffered saline containing 5% skim milk and 2% bovine albumin) at RT for 1 h. Five-fold serial-diluted mAbs were added to the plates and subsequently incubated for 1 h at 37°C. HRP-conjugated goat anti-human IgG (ZSGB-BIO, Beijing) secondary antibody was added to the plates and incubated at 37°C for 1 h. The enzymatic reaction was developed with 3,3′,5,5′-tetramethylbenzidine (TMB) substrate (Kinghawk, Beijing) and stopped by addition of 2M H_2SO_4. The absorbance was measured at 450 nm using a Varioskan^{TM} LUX Multimode Microplate Reader (Thermo Scientific). HIV-1-GP140 (purified in our lab) was an irrelevant antigen control.

Pseudovirus-based neutralization assay

The SARS-CoV-2 pseudovirus was generated through co-transfection of 293T cells with pVAX1-S and pNL4-3.Luc.R-E-, which carried the codon optimized SARS-CoV-2 S gene (GenBank: MN988668.1) and HIV-1 backbone, respectively. Viral supernatant was collected at 48 h post-transfection and frozen at -80°C. The serially diluted antibodies were incubated with equal volume pseudovirus at 37°C for 1 h. The antibody-virus mixtures were subsequently added onto 96-well plates which pre-seeded HEK 293T-ACE2 cells. After 48 h, infected cells were lysed to measure the luciferase activity using Bright-Glo Luciferase (Promega, Madison, WI) according to the manufacturer's protocol. The IC_{50} was determined by GraphPad Prism 7 using asymmetric (five parameters) model.

Focus reduction neutralization test

SARS-CoV-2 focus reduction neutralization test (FRNT) was performed in a certified Biosafety level 3 lab. Antibodies were 3-fold serially diluted and mixed with equal volume of SARS-CoV-2 live virus (containing 200 focus forming unit) on U-bottom 96-well plates. The mixtures were incubated for 60 min at 37 °C and next transferred onto the 96-well plate seeded with Vero E6 cells for 1 h at 37 °C before removed. After washing, the overlay media (MEM containing 1.6% Carboxymethylcellulose, 2% fetal bovine serum) was added and cells were incubated at 37 °C for 24 h. After removing the overlay media, cells were fixed with 4% paraformaldehyde solution, permeabilized with Perm/Wash buffer (BD Biosciences) containing 0.1% Triton X-100, incubated with HRP-conjugated anti-SARS-CoV-2-N IgG (isolated in our lab). The reactions were developed with KPL TrueBlue Peroxidase substrates (Seracare Life Sciences Inc). The numbers of SARS-CoV-2 foci were calculated using an EliSpot reader (Cellular Technology Ltd).

Note 5. Nucleotide sequences of the antibody candidates

Paired heavy and light chains of the antibodies are shown as follows:

>B2-C1_IGH

CAGGTGCAGCTACAGCAGTGGGGCGCGGGACTGTTGAAGCC
TTCGGAGACCCTGTCCCTCACCTGCGCTGTCTATGGTG
TGTCGCCCACTAGTTACTATTGGAGCTGGATCCGTCAG
TCCCCCGGGAAGGGTCTGGAGTGGATAGGGGAGATCA
CTCATAGTGGAAGCACCAACTACAATCCGTCCCTCAAGAGTC
GAGTCACCATGTCGCTGGACACGTCCAAGAGCC
AGTTCTCCCTGAAGTTGAGTTCTGTGACCGCCGCG
GACACGGCTATATATTATTGTGCGAGGGGACGC
AGTGAGGAGACCATGATAGTGATGGTTGTCACG
GGAATTGATTTCTACTTTGACTCTTGGGGCCAG
GGGACCCTGGTCACCGTCTCCTCA

>B2-C1_IGL

TCTTCTGAGCTGACTCAGGACCCTGCTGTGTCTGTGGCCTT
GGGACAGACAGTCAGGATCACATGCCAAGGAGACAACC
TCAAACTCTTTTATACAAACTGGTACCAGCAGAAGC
CAGGCCAGGCCCCCGTACTTGTCATCCATGGTAAAAAC
AACCGGCCCTCAGGGATCCCAGACCGATTCTCTGGCTCCAGTT
CAGCGTACACCACTTCCTTGACCATCATTGGGG
CTCAGGCGGAGGATGAGGCTGACTATTACTGTAGC
TCTCGCGACAGAAGTGGTGACCGTGTTATATTC
GGCGGAGGGACCAAGGTGACTGTCCTA

>B6-C2_IGH

GAGGTGCTCCTGGTGGAGTCTGGGGGAGGCTTGGTCCGGCC
TGGAGGGTCCCTAAGACTCTCCTGTGCAGCCTCTGGAT
TCACCTTCACTGACCACTATTTGGACTGGGTCCGCC
AGGCTCCAGGGATGGGGCTGGAGTGGGTTGGCCGTATT
AGAAATAAAGTTAATGGTTACACCACAGAATACGCCGCGTCTG
TGAAAGGCAGATTCACCATCTCAAGAGATGATT
CAAAGAACTCAGTTTATCTGCAAATGAATAGCCTG
AGAAGCGAGGACACGGCCGTGTATTACTGCACT
AGAGTGGGAGTTGGGAGCCCTGACTACTGGGGC
CAGGGAACCCTGGTCGCCGTCTCCTCA

>B6-C2_IGK

GACATCCAGATGACCCAGTCTCCATCCTCCCTGTCTGCATC
TGTAGGAGACAGAGTCACCATCACTTGCCGGGCAAGTCAGG
GCATTAGAGATGAGTTAGCCTGGTATCAGCAAAAACCAGGGA
AAGCCCCTAAGCGCCTGATCTATGATGCATCGAG
GTTGCAAAGTGGGATCCCATCGAGGTTCAGCGGCAGTGGATCT
GGGACAGAATTCACTCTCACAATCAGCAGTC
TGCAGCCTGAAGATTTTGCAACTTATCATTGTCTACAGT
ATACTAGTTACCCTCACACTTTTGGCCAGGGGAC
CAAGCTGGACATCAAA

>B6-C3_IGH

CAGGTGCAGCTACAACAGTGGGGCGCAGGACTGTTGAAGCC
TTCGGAGACCCTGTCCCTCACCTGCGCTGTCTATGGTGGGTC
CTTCAGTGGTTACCAGTGGAGGTGGATCCGCCAGGCCCCA
GGGAAGGGGCTGGAGTGGATTGGGGAAATCAATCATA
GTGGAAGCACCAATTACAACCCGTCCCTCAAGAGTCGA
GTCACCATATCAGTAGACACGTCCAAGAACCAGT
TCTCCCTGAGGTTGAGGTCTGTGACCGCCGCGGACACG
TCTGTGTATTTCTGTGCGAGAGGCCAAAATGGAGTA
GTTCCAGCTCCTGTATTGGGGATCGGACCTTACTACACC
TACTCCTACATGGACGTCTGGGGCACAGGGACCACGGTC
AGTGTCTCCTCA

>B6-C3_IGL

TCTTCTGAGCTGACTCAGGACCCTGCTGTGTCTGTGGCCT
TGGGACAGACAGTCAGGATCACATGCCAAGGAGACAGCCTCAG
AAGCTATTATGCAAGTTGGTACCAGCAGAAGCCAAGACAG
GCCCCTATTCTTGTCATCTATGGTAAAAACAATCGACCCT
CAGGGATCCCGGACCGATTCTCTGGCTCCTACTCAG
GAGCCACAGCTTCCTTAACCATCACTGGGGCTC
AGGCGGAGGATGAGGCTGACTATTATTGTGACTCCCGGGA
CAGCAGTGGTAACCATCGAGTGTTCGGCAGAGGGA
CCACGGTGACCGTGCTA

>B8-C1_IGH

CAGGTGCAACTGGTGCAGTCTGGGGCTGAGGTGAAGAAGCC
TGGGTCCTCGGTGAGGGTCTCCTGCCAGGCTTCTGGAGACAC
CTTCAGCAACTATGCTTTCAGTTGGGTGCGACAGGCCCCT
GGACAAGGGCTTGAGTGGATGGGAAGGATCATCCCTAT
CTTTGGAACACCAAACTACGCACAGAGGTTCCAGGGGAG
AGTCACGATTACCGCGGACGAGTCTACGAGGAC
AGCCTACATGGAATTGACCGGCCTGAGGTCTGACGACACGG
CCGTGTATTACTGTGCGAGACACACTTTGGTGAC
TGCTATTCAGAAGTGGGGCCAGGGAACCCTGGTCA
CCGTCTCCTCA

>B8-C1_IGK

GACATCCAGATGACCCAGTCTCCTTCCACCCTGTCTGCGTC
TGTTGGAGACAGAGTCACCATCACTTGCCGGGCCAGTCAGAG
TGTTAGTGACTGGTTGGCCTGGTATCAGCAGAAACCAGGG
GAGCCCCCTAAGCTCCTCATCTCTAGGGCATCTACTTT
AGAGATTGGGGTCGCATCAAGGTTCAGCGGCAGTGGAT
CTGGGACAGAATTCACTCTCACCATCAGCAGCCT
GCAGCCTGATGATTATGCAACTTATTACTGCCAACAGTATAAT
ACTTATTCGCTCACTTTCGGCGGAGGGACCAAGG
TGGAGATCAAA
