# Supplementary material for: SCIGA: Software for large-scale, single-cell immunoglobulin repertoire analysis
Source: Gigascience. 2021 Sep 28;10(9):giab050. doi: 10.1093/gigascience/giab050 (PMC8478610; doi:10.1093/gigascience/giab050)
Supplement: giab050_Response_to_Reviewer_Comments_Original_Submission [file giab050_response_to_reviewer_comments_original_submission.pdf]

Author's Response To Reviewer Comments

Close

The language of this manuscript has been revised by a language company. The software has received the RRID and Biotools ID (Line 244). We have fully addressed the points, as follows:

Reviewer #1: Ye and colleagues develop and describe a software for large scale, single-cell immunoglobulin repertoires analysis.

1) Are the methods appropriate to the aims of the study, are they well described, and are necessary controls included?
No. The sample processing, post-sequencing analysis and statistics is not fully discribed.
Response:
Thank you for the comment. The methods that describe the sample processing in Line 533-547, the post-sequencing analysis in Line 549-560, the statistics in Line 516-522.

2) Are the conclusions adequately supported by the data shown?
Partially, but there is not enough information in the manuscript, such as how the antibodies were selected.
Response:
Thank you for the comment. The method to select antibodies as follows: 1) Screen the clone with fraction ≥ 1% and cell number ≥ 20; 2) For each clone, screen the IgG immunoglobulin; 3) Trim the nucleotide sequences of immunoglobulin and retain the variable region; 4) Calculate the abundance of trimmed immunoglobulin sequences. The immunoglobulin sequence with the highest abundance in a clone is the monoclonal antibody candidate. (Line 564-569)

3) Please indicate the quality of language in the manuscript. Does it require a heavy editing for language and clarity?
Reasonable. Only a few corrections (below)
Response:
Thank you for the comment. The language of this manuscript has been revised by a language company.

4) Are you able to assess all statistics in the manuscript, including the appropriateness of statistical tests used?
No.
Response:
Thank you for the comment. The statistics in Line 516-522. But we didn't use statistical test in this study.

Major comments
* Productive BCRs are important in many situations, however, understanding the non-productive repertoire can be just as important in the context of haematological malignancies. Is this a possibility in your workflow? If not, this should be included.
Response:
Thank you for the comment. We modified the SCIGA program and retained the non-productive BCRs in the final result. A tag called "Productive" was added to denote whether the BCR is productive.

* Can the user introduce a custom reference for gene calling?
Response:
Thank you for the comment. Yes. Users can introduce a custom reference using the parameter "-index". Lesson to help users building the custom reference index in https://github.com/sciensic/SCIGA.

* Line 95: "The chains with a certainty score less than a specified threshold are discarded" can the authors explain how the threshold is calculated?
Response:
Thank you for the comment. The certainty score is defined as the number of UMIs supporting the chain

divided by the total number of UMIs of all heavy or light chains (Line 493-494). We set the default threshold as 80, followed the reference 7. Users can set a custom threshold using the parameter "- certainty".

* Line 97: "then the cells without pairing heavy- and light- chains are filtered out" Given the cost of sequencing by 10X, it is often helpful to include the analysis of cells with the capture of only one chain (such as clonality scores for rare cell subsets). Is there a possibility to include these?
Response:
Thank you for the comment.
Firstly, one of the important functions of 10X V(D)J sequencing is that 10X V(D)J sequencing can produce paired heavy and light repertoires. That why we prefer to just retain the cells with the capture of paired chains in the final results. However, SCIGA retains the intermediate results, including the cells with the capture of only one chain (in the file called "3_filtered_contig_annotation"). Users can use the intermediate results if they need to analyze the cells with only one chain.
Secondly, SCIGA groups clone using both heavy and light chains. It is hard to group a cell with only one chain into certain clonal lineage. For example, A cell has paired heavy and light chain, while B cell has only one identical heavy chain. We are not sure whether the A and B cell belong to a clonal lineage.

* Line 100: what is the threshold used for defining clones?
Response:
Thank you for the comment. We set the default threshold as 90 (Line 501), followed the reference 2. Users can set a custom threshold using the parameter "- identity".

* In figure 2C, sample B1 has moer cells passing filtering than CellRanger. Can the authors discuss why this sample is different from the rest?
Response:
Thank you for the comment.
The cell number mainly relates to the two aspects: 1) Immunoglobulin sequence assembly. SCIGA performs assembly using SSAKE. Only the barcodes with read number over a given threshold (default as 200) are performed assembly. This will speed up the software running, but fail to capture the cells with low V(D)J transcripts abundance. For the Cell Ranger, it performs assembly on all barcodes based on the De Bruijn graph. Cell Ranger considers the barcode as cell when the barcode includes a productive, confident contig. The two assembly methods make a difference on cell number. Generally, the cell number of SCIGA is less than Cell Ranger when the sequencing depth is low. 2) Cell quality controlling. For Cell Ranger, the output still included considerably low-quality cells, which either had multiple heavy or light chains, or had only one chain. SCIGA implements the cell quality control process and only output the high-quality cells.
In our dataset, the sample B1 had the highest sequencing depth (Figure R1 of the "Figures for responding to reviewers"). It meant that, more barcodes of sample B1 over the threshold in the read number, more high-quality contigs were assembled, and more cells pass the cell quality control. This may be the reason why sample B1 has more cells passing filtering than Cell Ranger.

* Most users will be comparing the clonality between multiple samples captured at differing cell depths. What have the authors done to address the issue of cell depth in the statistical analyses (such as subsampling etc), given that cell depth have a major impact on Simpson and Shannon indices? Was this point addressed in Figure 4?
Response:
Thank you for the comment. It is a controversial point. Some researchers suggested that subsampling analysis resolves randomly generated differences in sequence depth, but also results in discarding data, which leads to loss of assay power. (reference 20). Therefore, on one hand, SCIGA program computes the Simpson and Shannon indices using all cells of a sample. On the other hand, SCIGA program produces the rarefaction curves to show the value of two indices under different cell depth. (Figure R2 of the "Figures for responding to reviewers"). The rarefaction curves will be benefit to understand how the cell depth affect the two indices.

* Can the authors describe what the differences are between the Simpson and Shannon indices to guide the reader for their anlayses? A discussion of the pros and cons of each would be useful.
Response:
Thank you for the comment. A literature has discussed the difference between the two indices. (Bo-Ra Kim, et al. Deciphering Diversity Indices for a Better Understanding of Microbial Communities. Journal of Microbiology and Biotechnology). Both the two indices have specific biases. The Shannon index places a greater weight on clone richness. The value increases when the clone number increases. Whereas, the

Simpson index considers clone evenness more than clone richness in its measurement. It tends to indicate the clone dominance.

* Could the authors provide a plot or table of the clone sizes of the 4 expressed antibodies compared the rest of the BCR repertoire for each individual? How did the authors choose these antibodies? Were there only 4 clones that >1%/>20 cells, or were these selected. If the latter, please can the authors show how representative these expressed clones are of the total expanded IgG+ B cell population?
Response:
Thank you for the comment.
The plots of the clone sizes of the 4 expressed antibodies compared the rest of the BCR repertoire for each individual are in the Figure 4C and Figure S4. * denotes the antibody.
The method to select antibodies as follows: 1) Screen the clone with fraction ≥ 1% and cell number ≥ 20; 2) For each clone, screen the IgG immunoglobulin; 3) Trim the nucleotide sequences of immunoglobulin and retain the variable region; 4) Calculate the abundance of trimmed immunoglobulin sequences. The immunoglobulin sequence with the highest abundance in a clone is the monoclonal antibody candidate. (Line 564-569)
There were only 4 IgG clones that > 1% and > 20 cells.

* In line 205, they authors suggest that IGHV4-34 is associated with SARS-COV2. This cannot be deduced from the data provided given that there are no healthy controls for comparison. (It should be noted that this association has been seen in some other papers previously. However, I belive that IGHV3-33 is also associated which is not picked up here. It would be worth discussing this in the context of the current literature of BCR repertoire from COVID patients).
Response:
Thank you for the comment. The statement may be not serious, we removed it.

* Could the authors provide the SHM and IGHG information about the 4 expressed antibodies given that this is in the discussion (lines 211-213)?
Response:
Thank you for the comment. We added a table to show the SHM and IGHG information about the 4 expressed antibodies (Table S3). For the discussion, it was just about the strategy for screening antibody candidate. The reason why we didn’t exclude the antibody with SHM < 2% was that several potent neutralizing antibodies against SARS-CoV-2 have low SHM rates. However, we didn’t pick out the antibody with SHM < 2% using our strategy. Our statement may lead to misunderstanding, we re-phrased it (Line 218-223).

Minor comments
* The language used is informal at ponits, such as line 149 "who preferred to use IGHG1", instead of "with elevated IGHG1 usage". This needs to be tightened up as the preference of the patient (mental) is unlikely related to their IGH usage! Also line 146 "pretty high usage in patient B2". Also, line 164.
Response:
Thank you for the comment. This has been corrected. (Line 153 and 155-156)

* SHM rate is not measured (line 151), rather the level of SHM. This needs to be corrected. Also line 196.
Response:
Thank you for the comment. This has been corrected. (Line 157-158 and 206)

* Line 213: the word "Totally" does not make sense here.
Response:
Thank you for the comment. This has been corrected. (Line 223)

Reviewer #2: The manuscript entitled: "SCIGA: A software for large-scale, single-cell immunoglobulin repertories analysis" describes a computational platform for the single B cell analysis of data derived from 10xgenomics experiments. The authors state that SCIGA is superior to the challenger software especially by providing additional data regarding the characteristics of the identified antibodies and improves the sequence calls. The authors describe how SCIGA enables the isolation of 4 SARS-CoV-2 specific mAbs that exhibit neutralisation capacity.
Indeed, the rise in the usage of single cell platforms and particularly, 10xgenomics calls for the development of computational platforms to facilitate the data interpolation. This need was addressed in the submitted manuscript however, I would not recommend publishing the paper in its current version.

Beyond the comments specific below: the manuscript in not coherent and I had great difficulties reading it and at some paragraphs I completely was confused by some of the statements. The method section is lacking several key components (cloning and expression mAbs) and in general the manuscript requires a professional native English speaker, scientific editing.

Comments:
Line 79: "Since the 10x system generate a large of Gel…" - sentence not clear. All of the paragraph describing the cell call is not clear - please re-phrase paragraph.
Response:
Thank you for the comment. We have re-phrased this paragraph. (Line 79-82)

Line 94: State specifically the criteria for clone clustering.
Response:
Thank you for the comment. Clonal lineage is defined as the cells that have identical VH, JH, VL and JL genes, identical H-CDR3 length, and over a given similarity threshold (default value is 90) of H-CDR3 nucleotide sequences. The detail for clone clustering in Line 499-513.

Line 99: What is the similarity threshold ? 100%, 80% identity - on the basis of nt or AA ?
Response:
Thank you for the comment. We set the default threshold as 90 on the basis of nt, followed the reference 2. Users can set a custom threshold by using the parameter "- identity".

What is the meaning of V gene usage when analyzing B cell from PBMCs. There was no enrichment to B cell sub-types, so it would be good if the authors will clarify the biological meaning of the V gene usage for such a broad population of B cells. Furthermore, it is not clear why they used only 1% of the cells in this analysis.
Response:
Thank you for the comment.
The previous study said that the "unbiased" B-cell repertoire analysis will provide a more accurate view of the overall B-cell repertoire. (Linling He, et al. Toward a more accurate view of human B-cell repertoire by next-generation sequencing, unbiased repertoire capture and single-molecule barcoding. Scientific Report). Therefore, analyzing the V gene usage of a broad population of B cells can help knowing how the infection shapes the overall B-cell repertoire.
Under infection, the antigens will drive the specific B cells to proliferate, with the expansion of specific V gene. The elevated usage of V gene may be associated with the infection. Therefore, we only investigated the V gene with usage > 1%, followed the reference 2.

Line 149 - "who preferred to use…" - the patient did no prefer to use rather the response was characterised by …
Response:
Thank you for the comment. We have re-phrased this sentence. (Line 155-156)

In general for the section: " Profiling immunoglobulin repertoires of COVID-19 by SCIGA" - the fact that the authors did not use any sorting prior the single cell sequencing impacts the ability to generate meaningful insights regarding the repertoire of the antibodies. I am not sure that the data supports repertoire analysis. Maybe should not use the term repertoire.
Response:
Thank you for the comment. We think that plasma cells are important component of immunoglobulin repertoires. However, there is no marker that can sort the plasma cells out from PBMCs. That why we did not use any sorting prior the single cell sequencing.

Line 158-159: This is a circular logic - if both patients show high clonality - meaning that they have large clones. This sentence should be re-phrased.
Response:
Thank you for the comment. We have re-phrased this sentence. (Line 164-165)

Line 161: at line 158-159, the author state that patient B1 had large clones but here they state that patient B1 has even clone frequency.
Response:
Thank you for the comment. The statement was not correct and we have removed it.

Line 164: the clone do not prefer to use rather - there was a preferential usage of XYZ V family in the

clones…
Response:
Thank you for the comment. We have re-phrased this paragraph. (Line 171-174)

Line 169-170: This paragraph is not clear at all. Please re-phrase.
Response:
Thank you for the comment. We have re-phrased this paragraph. (Line 176-178)

Line 154: Clonal lineage grouping - it is not clear from the description what was the clonal lineage grouping serve ? insights regarding the repertoire ? a methodological strategy to identify monoclonal antibodies? Moreover, looking for shared clones in population of PBMC is not likely to yield a result - can the authors clarify how they found shared clones?
Response:
Thank you for the comment.
When encountering an antigen, the specific B cell will be activated and proliferate, forming the B cell clonal lineage. The expanded clonal lineage may be associated with the antigen. Grouping the clonal lineage can help find out the clones that response to the antigen.
The method to select antibodies as follows: 1) Screen the clone with fraction ≥ 1% and cell number ≥ 20; 2) For each clone, screen the IgG immunoglobulin; 3) Trim the nucleotide sequences of immunoglobulin and retain the variable region; 4) Calculate the abundance of trimmed immunoglobulin sequences. The immunoglobulin sequence with the highest abundance in a clone is the monoclonal antibody candidate. (Line 564-569)
Shared immunoglobulins are defined as the immunoglobulins from different samples that can be clustered into the same clonal lineage. (Line 529-531)

Line 175: it seems that the only criteria for identifying nt mAbs in the samples was frequency of the clones and cells. What about SHM? Was this taken inot account? What about isotype?
Response:
Thank you for the comment. We didn’t take into account the SHM, since several potent neutralizing antibodies against SARS-CoV-2 have low SHM rates (reference 16-18). The isotype of the selected antibodies must be IGHG.

Line 195: It is hard to conclude any insights regarding the repertoire of B cell (or BCRs) based on the analysis of single cells from PBMC. I recommend to re-phrase the statement to emphasis that the data can inform us on the V gene usage/SHM etc. on the identified cells by single cell analysis.
Response:
Thank you for the comment. Maybe you suggested the second paragraph of results (Title: Profiling immunoglobulin repertoires of COVID-19 by SCIGA). We have re-phrased the statement. The title was changed to “Determining the features of immunoglobulin repertoires of COVID-19 using SCIGA”.

Figure S4 - why to plot the CDR3 length of the heavy and light chain on the same graph. This is confusing.
Response:
Thank you for the comment. Maybe you suggested the Figure S3. We have modified the plot.

Methods: the authors did not describe how they - 1) sequenced the amplicons including the sequencer platform, what reagent kit was used for the sequencing, 2) the authors did not describe how they cloned and expressed and purified the monoclonal antibodies?
Response:
Thank you for the comment. 1) The method for sample processing and sequencing in Line 534-547. 2) The method for expressing and purifying the monoclonal antibodies in Line 570-578.

General comment: I would recommend the authors to use the services of scientific editor (native speaker) as many sentences require grammar revisions.
Response:
Thank you for the comment. The manuscript has been revised by a language company.

Close
