# Supplementary material for: SCIGA: Software for large-scale, single-cell immunoglobulin repertoire analysis
Source: Gigascience. 2021 Sep 28;10(9):giab050. doi: 10.1093/gigascience/giab050 (PMC8478610; doi:10.1093/gigascience/giab050)
Supplement: giab050_Response_to_Reviewer_Comments_Revision_1 [file giab050_response_to_reviewer_comments_revision_1.pdf]

Author's Response To Reviewer Comments

Close

Reviewer reports:
Reviewer #1: The authors have made significant efforts to improve their manuscript and have addressed most of the comments well. Whilst there is limited novelty of this method, there is utility and potentially opens up the analysis of this type of data to the readership of Gigascience who are less familiar with scVDJ sequencing analysis and interpretation. The identification of potential antibodies of interest is via "largest clonal abundance" which is a well-appreciated method which is usually only applicable during the peak of the B cell response to infection, however, will likely fail in most situations of chronic infection, autoimmunity or outside of the window of peak response.

Major comment:
* "The certainty score is defined as the number of UMIs supporting the chain divided by the total number of UMIs of all heavy or light chains (Line 493-494)." Either this needs rewording or readdressing: do you mean the sum of the UMIs for the most likely heavy + light chain divided by the total UMIs for that cell?

Thank you for the comment. We reworded this paragraph: For each reported heavy (light) chain, SCIGA calculates a certainty score, which is defined as the number of UMIs supporting the chain divided by the total number of UMIs of all heavy (light) chains for that cell. (Line 498-501)

Close
