# Supplementary material for: SCIGA: Software for large-scale, single-cell immunoglobulin repertoire analysis
Source: Gigascience. 2021 Sep 28;10(9):giab050. doi: 10.1093/gigascience/giab050 (PMC8478610; doi:10.1093/gigascience/giab050)
Supplement: giab050_Response_to_Reviewer_Comments_Revision_2 [file giab050_response_to_reviewer_comments_revision_2.pdf]

Author's Response To Reviewer Comments

Close

1) Since you submitted a PDF manuscript, can you make the following edits yourself then? In particular you will need to make some changes to the "Availability of supporting data" section. Please add a citation after "The data set(s) supporting the results of this article is(are) available in the [National Center for Biotechnology Information] repository (https://www.ncbi.nlm.nih.gov/), [PRJNA682839]." saying something like:
All supporting data and materials are available in the GigaScience GigaDB database [XX].
Then cite it as reference [XX] as:
Ye H; Cheng L; Ju B; Xu G; Liu Y; Zhang S; Wang L; Zhang Z. Supporting data for "SCIGA: A software for large-scale, single-cell immunoglobulin repertories analysis" GigaScience Database 2021. http://dx.doi.org/10.5524/100893.

Response:
Thank you for the comment. We added this citation in Line 273.

2) Also, please add the ORCIDs number for all authors (who have it) before "Abstract" like this:
ORCIDs:
Yujing Suo, 0000-0002-1080-5094;
Peng Sun, 0000-0001-5746-9684.

Response:
Thank you for the comment. We added the ORCIDs in Line 26 ~ 32.

3) And add the office phone number of the corresponding author(s) if available.

Response:
Thank you for the comment. We added the office phone number of the corresponding authors in Line 20 ~21 and Line 23 ~ 24.

Close
