# Supplementary material for: SCIGA: Software for large-scale, single-cell immunoglobulin repertoire analysis
Source: Gigascience. 2021 Sep 28;10(9):giab050. doi: 10.1093/gigascience/giab050 (PMC8478610; doi:10.1093/gigascience/giab050)
Supplement: giab050_Reviewer_1_Report_Original_Submission — Rachael Bashford-Rogers -- 1/13/2021 Reviewed [file giab050_reviewer_1_report_original_submission.pdf]

Reviewer Report

Title: SCIGA: A software for large-scale, single-cell immunoglobulin repertories analysis

Version: Original Submission **Date:** 1/13/2021

Reviewer name: Rachael Bashford-Rogers

Reviewer Comments to Author:

Ye and colleagues develop and describe a software for large scale, single-cell immunoglobulin repertoires analysis.
1) Are the methods appropriate to the aims of the study, are they well described, and are necessary controls included?
No. The sample processing, post-sequencing analysis and statistics is not fully discribed.
2) Are the conclusions adequately supported by the data shown?
Partially, but there is not enough information in the manuscript, such as how the antibodies were selected.
3) Please indicate the quality of language in the manuscript. Does it require a heavy editing for language and clarity?
Reasonable. Only a few corrections (below)
4) Are you able to assess all statistics in the manuscript, including the appropriateness of statistical tests used?
No.
Major comments
* Productive BCRs are important in many situations, however, understanding the non-productive repertoire can be just as important in the context of haematological malignancies. Is this a possibility in your workflow? If not, this should be included.
* Can the user introduce a custom reference for gene calling?
* Line 95: "The chains with a certainty score less than a specified threshold are discarded" can the authors explain how the threshold is calculated?
* Line 97: "then the cells without pairing heavy- and light- chains are filtered out" Given the cost of sequencing by 10X, it is often helpful to include the analysis of cells with the capture of only one chain (such as clonality scores for rare cell subsets). Is there a possibility to include these?
* Line 100: what is the threshold used for defining clones?
* In figure 2C, sample B1 has moer cells passing filtering than CellRanger. Can the authors discuss why this sample is different from the rest?
* Most users will be comparing the clonality between multiple samples captured at differing cell depths. What have the authors done to address the issue of cell depth in the statistical analyses (such as subsampling etc), given that cell depth have a major impact on Simpson and Shannon indices? Was this point addressed in Figure 4?
* Can the authors describe what the differences are between the Simpson and Shannon indices to guide the reader for their anlayses? A discussion of the pros and cons of each would be useful.

* Could the authors provide a plot or table of the clone sizes of the 4 expressed antibodies compared the rest of the BCR repertoire for each individual? How did the authors choose these antibodies? Were there only 4 clones that >1%/>20 cells, or were these selected. If the latter, please can the authors show how representative these expressed clones are of the total expanded IgG+ B cell population?
* In line 205, they authors suggest that IGHV4-34 is associated with SARS-COV2. This cannot be deduced from the data provided given that there are no healthy controls for comparison. (It should be noted that this association has been seen in some other papers previously. However, I belive that IGHV3-33 is also associated which is not picked up here. It would be worth discussing this in the context of the current literature of BCR repertoire from COVID patients).
* Could the authors provide the SHM and IGHG information about the 4 expressed antibodies given that this is in the discussion (lines 211-213)?

Minor comments

* The language used is informal at ponits, such as line 149 "who preferred to use IGHG1", instead of "with elevated IGHG1 usage". This needs to be tightened up as the preference of the patient (mental) is unlikely related to their IGH usage! Also line 146 "pretty high usage in patient B2". Also, line 164.
* SHM rate is not measured (line 151), rather the level of SHM. This needs to be corrected. Also line 196.
* Line 213: the word "Totally" does not make sense here.

Level of Interest

Please indicate how interesting you found the manuscript: Choose an item.

Quality of Written English

Please indicate the quality of language in the manuscript: Choose an item.

Declaration of Competing Interests

Please complete a declaration of competing interests, considering the following questions:

- Have you in the past five years received reimbursements, fees, funding, or salary from an organisation that may in any way gain or lose financially from the publication of this manuscript, either now or in the future?
- Do you hold any stocks or shares in an organisation that may in any way gain or lose financially from the publication of this manuscript, either now or in the future?
- Do you hold or are you currently applying for any patents relating to the content of the manuscript?
- Have you received reimbursements, fees, funding, or salary from an organization that holds or has applied for patents relating to the content of the manuscript?
- Do you have any other financial competing interests?

- Do you have any non-financial competing interests in relation to this paper?

If you can answer no to all of the above, write 'I declare that I have no competing interests' below. If your reply is yes to any, please give details below.

I declare that I have no competing interests

I agree to the open peer review policy of the journal. I understand that my name will be included on my report to the authors and, if the manuscript is accepted for publication, my named report including any attachments I upload will be posted on the website along with the authors' responses. I agree for my report to be made available under an Open Access Creative Commons CC-BY license (http://creativecommons.org/licenses/by/4.0/). I understand that any comments which I do not wish to be included in my named report can be included as confidential comments to the editors, which will not be published.

Choose an item.

To further support our reviewers, we have joined with Publons, where you can gain additional credit to further highlight your hard work (see: https://publons.com/journal/530/gigascience). On publication of this paper, your review will be automatically added to Publons, you can then choose whether or not to claim your Publons credit. I understand this statement.

Yes Choose an item.
