# Supplementary material for: SCIGA: Software for large-scale, single-cell immunoglobulin repertoire analysis
Source: Gigascience. 2021 Sep 28;10(9):giab050. doi: 10.1093/gigascience/giab050 (PMC8478610; doi:10.1093/gigascience/giab050)
Supplement: giab050_Reviewer_1_Report_Revision_1 — Rachael Bashford-Rogers -- 4/6/2021 Reviewed [file giab050_reviewer_1_report_revision_1.pdf]

Reviewer Report

Title: SCIGA: A software for large-scale, single-cell immunoglobulin repertories analysis

Version: Revision 1 **Date:** 4/6/2021

Reviewer name: Rachael Bashford-Rogers

Reviewer Comments to Author:

The authors have made significant efforts to improve their manuscript and have addressed most of the comments well. Whilst there is limited novelty of this method, there is utility and potentially opens up the analysis of this type of data to the readership of Gigascience who are less familiar with scVDJ sequencing analysis and interpretation. The identification of potential antibodies of interest is via "largest clonal abundance" which is a well-appreciated method which is usually only applicable during the peak of the B cell response to infection, however, will likely fail in most situations of chronic infection, autoimmunity or outside of the window of peak response.
Major comment:
* "The certainty score is defined as the number of UMIs supporting the chain divided by the total number of UMIs of all heavy or light chains (Line 493-494)." Either this needs rewording or readdressing: do you mean the sum of the UMIs for the most likely heavy + light chain divided by the total UMIs for that cell?

Level of Interest

Please indicate how interesting you found the manuscript: Choose an item.

Quality of Written English

Please indicate the quality of language in the manuscript: Choose an item.

Declaration of Competing Interests

Please complete a declaration of competing interests, considering the following questions:

- Have you in the past five years received reimbursements, fees, funding, or salary from an organisation that may in any way gain or lose financially from the publication of this manuscript, either now or in the future?
- Do you hold any stocks or shares in an organisation that may in any way gain or lose financially from the publication of this manuscript, either now or in the future?
- Do you hold or are you currently applying for any patents relating to the content of the manuscript?

- Have you received reimbursements, fees, funding, or salary from an organization that holds or has applied for patents relating to the content of the manuscript?
- Do you have any other financial competing interests?
- Do you have any non-financial competing interests in relation to this paper?

If you can answer no to all of the above, write 'I declare that I have no competing interests' below. If your reply is yes to any, please give details below.

I declare that I have no competing interests

I agree to the open peer review policy of the journal. I understand that my name will be included on my report to the authors and, if the manuscript is accepted for publication, my named report including any attachments I upload will be posted on the website along with the authors' responses. I agree for my report to be made available under an Open Access Creative Commons CC-BY license (http://creativecommons.org/licenses/by/4.0/). I understand that any comments which I do not wish to be included in my named report can be included as confidential comments to the editors, which will not be published.

Choose an item.

To further support our reviewers, we have joined with Publons, where you can gain additional credit to further highlight your hard work (see: https://publons.com/journal/530/gigascience). On publication of this paper, your review will be automatically added to Publons, you can then choose whether or not to claim your Publons credit. I understand this statement.

Yes Choose an item.
