# Supplementary material for: SCIGA: Software for large-scale, single-cell immunoglobulin repertoire analysis
Source: Gigascience. 2021 Sep 28;10(9):giab050. doi: 10.1093/gigascience/giab050 (PMC8478610; doi:10.1093/gigascience/giab050)
Supplement: giab050_Reviewer_2_Report_Original_Submission — Yariv Wine -- 2/20/2021 Reviewed [file giab050_reviewer_2_report_original_submission.pdf]

Reviewer Report

Title: SCIGA: A software for large-scale, single-cell immunoglobulin repertories analysis

Version: Original Submission **Date:** 2/20/2021

Reviewer name: Yariv Wine

Reviewer Comments to Author:

The manuscript entitled: "SCIGA: A software for large-scale, single-cell immunoglobulin repertories analysis" describes a computational platform for the single B cell analysis of data derived from 10xgenomics experiments. The authors state that SCIGA is superior to the challenger software especially by providing additional data regarding the characteristics of the identified antibodies and improves the sequence calls. The authors describe how SCIGA enables the isolation of 4 SARS-CoV-2 specific mAbs that exhibit neutralisation capacity.

Indeed, the rise in the usage of single cell platforms and particularly, 10xgenomics calls for the development of computational platforms to facilitate the data interpolation. This need was addressed in the submitted manuscript however, I would not recommend publishing the paper in its current version. Beyond the comments specific below: the manuscript in not coherent and I had great difficulties reading it and at some paragraphs I completely was confused by some of the statements. The method section is lacking several key components (cloning and expression mAbs) and in general the manuscript requires a professional native English speaker, scientific editing.

Comments:

Line 79: "Since the 10x system generate a large of Gel…" - sentence not clear. All of the paragraph describing the cell call is not clear - please re-phrase paragraph.

Line 94: State specifically the criteria for clone clustering.

Line 99: What is the similarity threshold ? 100%, 80% identity - on the basis of nt or AA ?

What is the meaning of V gene usage when analyzing B cell from PBMCs. There was no enrichment to B cell sub-types, so it would be good if the authors will clarify the biological meaning of the V gene usage for such a broad population of B cells. Furthermore, it is not clear why they used only 1% of the cells in this analysis.

Line 149 - "who preferred to use…" - the patient did no prefer to use rather the response was characterised by …

In general for the section: " Profiling immunoglobulin repertoires of COVID-19 by SCIGA" - the fact that the authors did not use any sorting prior the single cell sequencing impacts the ability to generate meaningful insights regarding the repertoire of the antibodies. I am not sure that the data supports repertoire analysis. Maybe should not use the term repertoire.

Line 158-159: This is a circular logic - if both patients show high clonality - meaning that they have large clones. This sentence should be re-phrased.

Line 161: at line 158-159, the author state that patient B1 had large clones but here they state that patient B1 has even clone frequency.

Line 164: the clone do not prefer to use rather - there was a preferential usage of XYZ V family in the

clones…
Line 169-170: This paragraph is not clear at all. Please re-phrase.
Line 154: Clonal lineage grouping - it is not clear from the description what was the clonal lineage grouping serve ? insights regarding the repertoire ? a methodological strategy to identify monoclonal antibodies? Moreover, looking for shared clones in population of PBMC is not likely to yield a result - can the authors clarify how they found shared clones?
Line 175: it seems that the only criteria for identifying nt mAbs in the samples was frequency of the clones and cells. What about SHM? Was this taken inot account? What about isotype?
Line 195: It is hard to conclude any insights regarding the repertoire of B cell (or BCRs) based on the analysis of single cells from PBMC. I recommend to re-phrase the statement to emphasis that the data can inform us on the V gene usage/SHM etc. on the identified cells by single cell analysis.
Figure S4 - why to plot the CDR3 length of the heavy and light chain on the same graph. This is confusing.
Methods: the authors did not describe how they - 1) sequenced the amplicons including the sequencer platform, what reagent kit was used for the sequencing, 2) the authors did not describe how they cloned and expressed and purified the monoclonal antibodies?
General comment: I would recommend the authors to use the services of scientific editor (native speaker) as many sentences require grammar revisions.

Level of Interest

Please indicate how interesting you found the manuscript: Choose an item.

Quality of Written English

Please indicate the quality of language in the manuscript: Choose an item.

Declaration of Competing Interests

Please complete a declaration of competing interests, considering the following questions:

- Have you in the past five years received reimbursements, fees, funding, or salary from an organisation that may in any way gain or lose financially from the publication of this manuscript, either now or in the future?
- Do you hold any stocks or shares in an organisation that may in any way gain or lose financially from the publication of this manuscript, either now or in the future?
- Do you hold or are you currently applying for any patents relating to the content of the manuscript?
- Have you received reimbursements, fees, funding, or salary from an organization that holds or has applied for patents relating to the content of the manuscript?

- Do you have any other financial competing interests?
- Do you have any non-financial competing interests in relation to this paper?

If you can answer no to all of the above, write 'I declare that I have no competing interests' below. If your reply is yes to any, please give details below.

I declare that I have no competing interests

I agree to the open peer review policy of the journal. I understand that my name will be included on my report to the authors and, if the manuscript is accepted for publication, my named report including any attachments I upload will be posted on the website along with the authors' responses. I agree for my report to be made available under an Open Access Creative Commons CC-BY license (http://creativecommons.org/licenses/by/4.0/). I understand that any comments which I do not wish to be included in my named report can be included as confidential comments to the editors, which will not be published.

Choose an item.

To further support our reviewers, we have joined with Publons, where you can gain additional credit to further highlight your hard work (see: https://publons.com/journal/530/gigascience). On publication of this paper, your review will be automatically added to Publons, you can then choose whether or not to claim your Publons credit. I understand this statement.

Yes Choose an item.
